# Parsimonious Gene Correlation Network Analysis (PGCNA): a tool to define modular gene co-expression for refined molecular stratification in cancer

**DOI:** 10.1038/s41540-019-0090-7

**Published:** 2019-04-11

**Authors:** Matthew A. Care, David R. Westhead, Reuben M. Tooze

**Affiliations:** 1 Section of Experimental Haematology, Leeds Institute of Medical Research, University of Leeds, Leeds, LS9 7TF UK; 2 Bioinformatics Group, School of Molecular and Cellular Biology, University of Leeds, Leeds, LS2 9JT UK

**Keywords:** Cancer, Molecular medicine, Computational biology and bioinformatics, Biomarkers

## Abstract

Cancers converge onto shared patterns that arise from constraints placed by the biology of the originating cell lineage and microenvironment on programs driven by oncogenic events. Here we define consistent expression modules reflecting this structure in colon and breast cancer by exploiting expression data resources and a new computationally efficient approach that we validate against other comparable methods. This approach, Parsimonious Gene Correlation Network Analysis (PGCNA), allows comparison of network structures between these cancer types identifying shared modules of gene co-expression reflecting: cancer hallmarks, functional and structural gene batteries, copy number variation and biology of originating lineage. These networks along with the mapping of outcome data at gene and module level provide an interactive resource that generates context for relationships between genes within and between such modules. Assigning module expression values (MEVs) provides a tool to summarize network level gene expression in individual cases illustrating potential utility in classification and allowing analysis of linkage between module expression and mutational state. Exploiting TCGA data thus defines both recurrent patterns of association between module expression and mutation at data-set level, and exemplifies the polarization of mutation patterns with the leading edge of module expression at individual case level. We illustrate the scalable nature of the approach within immune response related modules, which in the context of breast cancer demonstrates the selective association of immune subsets, in particular mast cells, with the underlying mutational pattern. Together our analyses provide evidence for a generalizable framework to enhance molecular stratification in cancer.

## Gene expression networks: radical pruning enhances resolution

To control cell behavior in physiology and disease, genes are expressed in co-ordinated networks, but the sheer number of connections between genes challenges interpretation of this structure. Care, Westhead and Tooze—University of Leeds, UK—show how retaining only the 3 nearest neighbors of any gene improves information content of expression networks. This new approach, PGCNA, outperforms alternatives and can be used to integrate multiple sets of data. With breast and colon cancer as examples the team demonstrate how such networks can be used to derive bar-codes that summarize cancer gene expression and link to underlying gene mutation states. This has potential application in precision medicine and establishes a scalable, efficient, approach to enhance network analysis.

## Introduction

A primary driver in tumor classification is enhanced precision through molecular characterization. Such analysis provides an increasingly complex view of individual tumor biology,^1^ resulting in the concept of combinatorial characterization using multiple platforms. An extension is provided by pan-cancer classification where cases associated with key molecular features are combined potentially across the boundaries of conventional classification.^2^

Gene expression-based classifications have defined both prognostically and pathogenetically distinct cancer subtypes,^3–6^ which have preferential association with mutational and cytogenetic profiles.^7^ Use of reduced sets of genes allows recognition of subtypes in applied classifications.^8,9^ The cancer hallmark paradigm postulates that aberrantly regulated features assemble in modular fashion to promote malignancy.^10^ Thus, an integrated assessment of these features might also take a modular approach within individual cancers.

With multiple data-sets the pattern of correlation between individual pairs of genes can be used to determine intrinsic modules of gene co-expression.^11^ Exemplifying how modular patterns of co-expression can be identified within the overall profile of a tumor, gene expression allows inference of tumor infiltrating immune populations.^12,13^ Existing expression data-sets provide an extensive resource for individual types of cancer, which sit alongside multiparameter analysis across diverse cancer types in resources such as TCGA. We considered that a network analysis of cancer expression data could provide added value by generating a visual structure that contextualizes patterns of gene co-expression across large data-sets, but given the complexity of expression data this would depend on efficient reduction in the number of edges connecting genes in the network. A test for such a data-led approach lies in the ability to resolve modules of co-expressed genes associated with known meaningful biology within and between cancer types. We reasoned that by addressing these goals using an unsupervised method, without prior assumptions, we would be able to assess the resulting networks against pre-existing paradigms and as a platform for molecular stratification of cancer. In the latter context we envisaged mapping the discovered modules onto gene expression data-sets to generate module ‘fingerprints’ per sample/patient and using these derived ‘fingerprints’ to study relationships between module biology and external factors such as gene mutation state.

Providing a conceptual framework for this study, previous successful methods for network analysis have generated significant insights in model systems and clinical data.^14–17^ However, such approaches are not intrinsically designed to generate networks across multiple data-sets. Furthermore, a common challenge in extracting meaning from network-based analysis is the potentially very high density of connectivity. Although this can be successfully negotiated using approaches that focus onto modular patterns of gene expression.^18^

Here we test a conceptually simple, parsimonious approach to the problem of connectivity reduction as a means of deriving modular expression networks across the gene expression data resource for breast and colon cancer. We test the utility of the resulting networks as a platform to explore multi-parameter data such as TCGA.

## Results

### Parsimony enhances gene expression network clustering

A challenge in analyzing gene co-expression patterns is how to exploit multiple data-sets, with potentially very large numbers of genes and correlations, to derive an integrated and tractable visualization of gene co-expression patterns, while also generating modules of gene co-expression that can be used in downstream analyses of related data. We reasoned that a parsimonious approach in which only a restricted number of the most significant correlations (edges) per gene (node) are retained might provide a focusing effect in network analysis. To address this, we developed a method in which the correlation patterns of gene co-expression are integrated across multiple data-sets, by first deriving a per-data-set correlation matrix, and then combing these to generate a merged correlation matrix based on the median correlations and *p*-values across different data-sets. A particular challenge lies in the high degree of connectivity of these data, resulting in difficulty interpreting the relationships between nodes. To address this issue, we conceived of a simple approach in which for each gene only the edges reflecting the most highly correlated genes are retained (Edge Per Gene: EPG; where EPG3 = retaining 3 edges per gene). These are assembled into a matrix in which a gene may retain additional correlations if it represents a common partner of other genes in the matrix (Supplemental Fig. 1 for methods outline and Supplemental Methods for further details; both in Supplemental Materials). We applied this approach to expression data-sets for breast cancer (BRCA, *n* = 26 expression data-sets) and colorectal cancer (CRC, *n* = 11 expression data-sets). Applying the radical edge reduction thresholds of EPG3—EPG10 resulted in a linear relationship with total edges in the network, reducing edges by a factor of between 250 (EPG10) to 900-fold (EPG3) (Supplemental Table 1). The resulting parsimonious correlation matrices were tested in network generation.

**Fig. 1:**
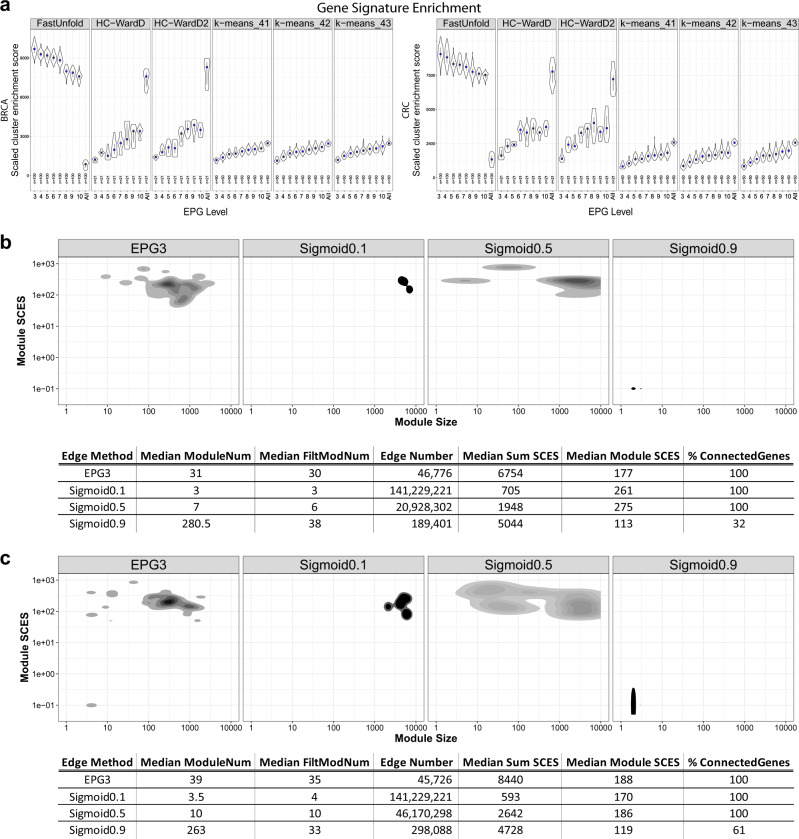
Radical edge reduction enhances the resolution of biology in gene co-expression modules. **a** Enrichment of gene ontology and signatures was assessed using a scaled cluster enrichment score (SCES) and compared between data clustering generated with FastUnfold, Hierarchical clustering or k-means clustering using either the total correlation data (All) or parsimonious matrices with edges per gene (EPG) thresholds between 3 and 10. Violin plots display the distribution along with median (blue square) and the IQR. **b**, **c** density plots of the module size (gene-number; *x*-axis) vs module SCES (Scaled Cluster Enrichment Score; see methods; *y*-axis) across the 100 best clustering for EPG3 (Edge Per Gene 3) or the WGCNA sigmoid adjacency function varying the shift (μ, 0.1, 0.5 and 0.9). **b** BRCA data and **c** CRC data. Beneath the graph the results are displayed in tabular form, showing the median module number, median modular number with gene membership >5, edge number retained after filtering (edges <0.01 removed), median sum of module SCES, median module SCES, and the percentage of connected genes (after filtering; edges <0.01 removed)

Clusters of gene co-expression were derived from correlation matrices using three approaches: hierarchical clustering, K-means clustering or a computationally efficient network tool, fast unfolding of communities in large networks (FastUnfold).^19^ In each instance clusters were generated from matrices in which genes retained all edges (edges with *p*-value >0.05 set to zero and thus removed) or parsimonious matrices (EPG3—EPG10; Supplemental Table 1). To compare the resulting partitioning of the data, the clusters of co-expression (subsequently referred to as modules, Supplemental Table 2) were then tested for the separation of known biology, based on enrichment of ontology and signature terms. This was assessed using a scaled cluster enrichment score (SCES), which takes into account the significance of enrichment of ontology and signature terms within a module, the extent to which the enrichment of signature or ontology term is specific to an individual module rather than being shared across multiple modules, and the balance of gene number associated with individual modules. Strikingly, when using EPG parsimonious matrices, the network method (FastUnfold) provided the most significant enrichment and segregation of ontology terms. This was a particular feature of the combination of FastUnfold with parsimonious matrices as applying FastUnfold to the total correlation matrix without parsimonious edge reduction resulted in poor separation of the data (Fig. 1a). For each cancer type, decreasing the retained edge per gene (minimum EPG3) led to an increasing number of resolved modules (Supplemental Fig. 2a). This corresponded to an improved segregation of biology between modules across both cancers (Fig. 1a). Indeed, there was no significant benefit to retaining more than 3 edges per gene (EPG3), while at this level of edge reduction the number of modules remained manageable for downstream analysis (Supplemental Fig. 2a). Notably the use of parsimonious matrices in conjunction with FastUnfold outperformed both hierarchical and k-means clustering of the data even where all edges were retained. While parsimonious matrices did not confer benefit to the separation of data when using these other clustering methods.

For the EPG3/FastUnfold combination robustness was tested for the best 100 clusterings, showing that for each cancer type modules retained a high proportion of the same genes across different clustering runs (Supplemental Fig. 2b, c). Network solutions generated with the combination of FastUnfold and parsimonious matrices were scale-free across all the EPG thresholds tested (Supplemental Fig. 2d). This is not the case for application of FastUnfold to the total untrimmed correlation data (filtered by hard threshold; Supplemental Fig. 2e) which also failed to generate effective resolution of biology between modules. Thus, we concluded that a combination of edge reduction with the FastUnfold approach enhanced the resulting network solution in gene correlation data.

Next to assess whether EPG edge reduction with FastUnfold provides a particular advantage we compared different edge refinement methods. This compared the EPG3 approach against iPCC,^20^ a power function or the sigmoid functions of the Weighted Gene Correlation Network Analysis package (WGCNA),^15^ as input into FastUnfold. Because WGCNA is mainly aimed at single data-set analysis we evaluated these four approaches using representative large expression data-sets for BRCA and CRC. The results were compared in terms of the retained edge number (edges <0.01 removed; providing an indication of the degree of edge reduction), the total number of modules resolved in the data, the number of modules with >5 genes per module (as a reflection of balanced module size), the median module SCES as indicator of separation of biology between modules, the median sum of SCES as an indicator of the total enrichment of biology across the network, and the percentage of genes connected in the network. Overall when compared against the other methods the combination of EPG3 with FastUnfold provided both a suitable module number and size for downstream evaluation and the greatest sum of biological signature enrichments across the network. Neither iPCC or power function produced a substantial edge reduction and when used in combination with FastUnfold this resulted in separation of the data into 2–4 modules, which provides insufficient resolution of underlying biology (Supplemental Figs 3 and 4). In contrast, the Sigmoid function at higher μ thresholds effectively reduced edge number (Fig. 1b, c, and Supplemental Figs 3 and 4) but did so at the expense of generating large numbers of very small modules with less than 5 gene members per module, as well as a progressive increase in the percentage of orphan genes unconnected to the network. The median sum SCES increased for the Sigmoid function at higher μ levels yet remained below that of the EPG3 network, with the latter also retaining 100% gene connectivity as compared to 32/61% for the highest Sigmoid μ in BRCA/CRC respectively. We therefore conclude that the combination of EPG3 matrices clustered with FastUnfold provides added value, and refer to this combination as Parsimonious Gene Correlation Network Analysis (PGCNA).

In order to provide a further assessment of the potential value of this approach we tested its relative performance against the WGCNA package as a whole, which has been shown to provide a very effective tool for clustering diverse data types,^15^ and was used for comparison for this reason. Since WGCNA is primarily intended for analyzing single sets of expression data and has not been specifically designed to handle a correlation matrix of the type generated by merging multiple data-sets as applied here, we again performed this comparative analysis using single representative expression data-sets. WGCNA uses the concept of scale-free networks to aid parameter selection. As noted above, PGCNA generated scale-free topologies across all EPG thresholds tested (Supplemental Fig. 2d). Comparing gene ontology and signature enrichment using the SCES, indicated a greater enrichment for the PGCNA solutions relative to WGCNA (Supplemental Fig. 5). Considering the stability of module gene membership across soft threshold levels employed in WGCNA and EPG levels in PGCNA, the PGCNA method demonstrated a higher degree of stability for gene membership (Supplemental Figs 6–9). Of note, in performing the technical comparison of the clustering approaches the EPG/FastUnfold combination (PGCNA) was also highly efficient in terms of computational time and memory usage (Supplemental Methods). Thus the combination of parsimonious edge reduction and FastUnfold analysis in addition to providing enhanced resolution of biology between network modules, can provide benefits in terms of computational efficiency.

### Biology of network modules and mapping to expression-based cancer classifications

The above analysis established that PGCNA provided an effective tool for analysis of gene co-expression providing advantages relative to pre-existing methods; producing tractable networks that allow contextualization of all nodes and edges used in network generation and that clusters these into a small number (<50) of biologically distinct modules. To further evaluate the utility of this approach the optimum PGCNA clustering based on SCES was taken forward for detailed downstream analysis for each cancer type.

**Fig. 2:**
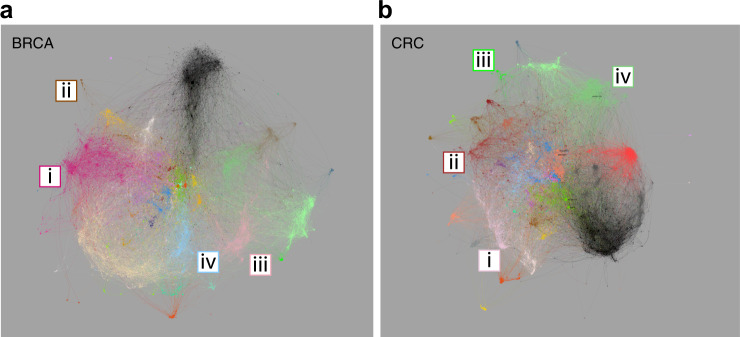
Network visualization for BRCA and CRC. **a** BRCA network with modules color-coded, modules overlapping significantly with those in CRC share a common color. Modules corresponding to intrinsic BRCA classification (i) luminal (BRCA_M6), (ii) ERBB2/HER2 (BRCA_M5), (iii) basal/normal (BRCA_M14) and (iv) cell cycle (BRCA_M7). **b** CRC network, highlighted modules correspond to consensus molecular subtypes of CRC (i) CMS2-enterocyte (CRC_M3), (ii) CMS3-metabolic/goblet (CRC_M7), (iii) CMS1-hypermutated (CRC_M32) and (iv) CMS4-mesenchymal (CRC_M8). Fully annotated versions in Supplemental Fig. 10, and http://pgcna.gets-it.net/

Initially the networks were visualized as an interactive web-based resource (Fig. 2a, b). To enhance network utility additional factors were overlaid providing inter-related visualizations of the data viewed through the networks (Supplemental Fig. 10 & http://pgcna.gets-it.net/). Indeed, one of the impacts of radical edge reduction is the generation of sparser and visually navigable networks, in which nonetheless all genes remain connected and all edges used in network generation can be visualized. This is particularly the case when viewed as an interactive resource, which provides a context for assessing the relationships of gene co-expression, for example, in relation to genes linked to existing expression-based cancer classification schemes.

Detection of BRCA intrinsic sub-classes has been refined into expression-based tools such as the PAM50 classifier.^4,5,8^ Mapping genes linked to these intrinsic classes onto the network identifies BRCA_M6 as the luminal module (Fig. 2a [i], Supplemental Fig. 10a–c, online). Genes associated with ERBB2 amplified breast cancer map on to BRC_M5 (Fig. 2a [ii]), epithelial genes defining basal breast cancer overlap with those linked to normal-like breast cancers and map onto module BRCA_M14 (Fig. 2a [iii]). While genes linked to cell proliferation which provide a shared feature of Luminal B and Basal type breast cancers map onto BRCA_M7 (Fig. 2a [iv]). An example here of the networks providing context for patterns of gene expression is the close linkage within the network between BRCA_M14 and BRCA_M7, which together encompass genes that define basal type breast in the PAM50 classification.

The CRC consensus molecular subtype classification recognizes four subtypes^6^: CMS2 containing genes linked to canonical enterocyte-like differentiation maps onto module CRC_M3 (Fig. 2b [i]); CMS3 reflects goblet-cell and metabolic differentiation and maps onto CRC_M7 (Fig. 2b [ii]); CMS1 identifies microsatellite unstable cancers through interferon response genes and maps onto CRC_M32 (Fig. 2b [iii]); and CMS4 encompassing mesenchymal dominant CRC maps onto CRC_M8 (Fig. 2b [iv]) (Supplemental Fig. 10d–f, online). Again, in this network the context of gene expression relationships between enterocyte and goblet cell differentiation is illustrated by the proximity of these modules in the network, and their distinct separation from the mesenchymal module. Therefore, the PGCNA networks successfully place current paradigms of expression-based classification in BRCA and CRC in the context of wider expression patterns for each cancer.

**Fig. 3:**
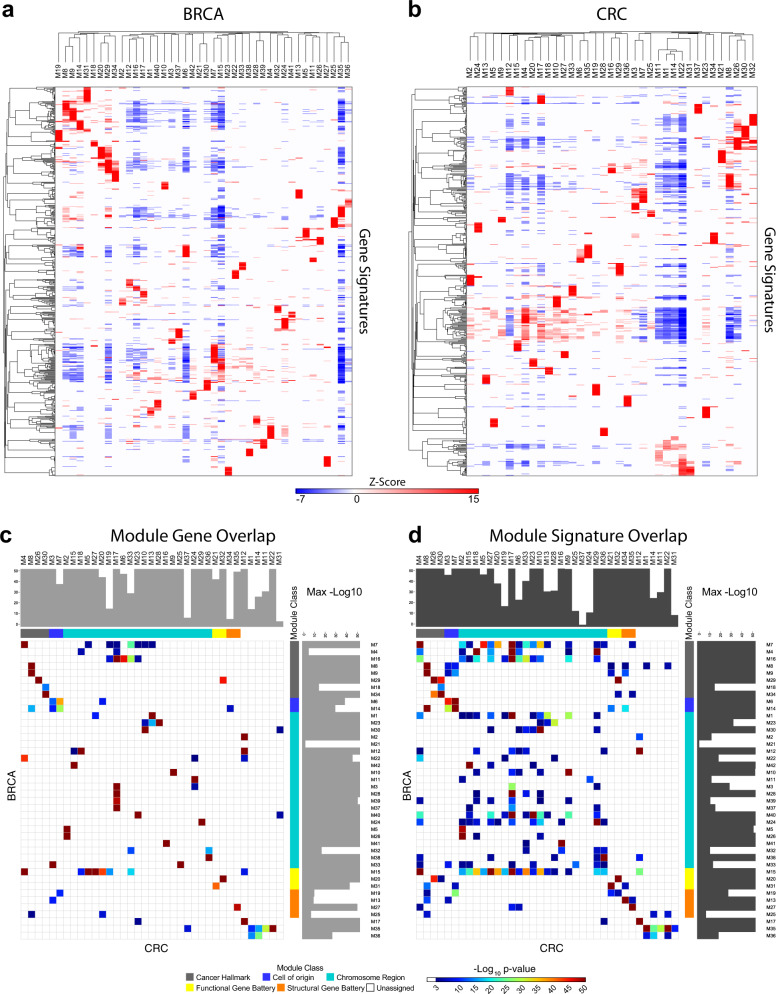
Module biology and between cancer analysis identifies principles of gene co-expression. Heatmaps of gene signature enrichment between modules **a** BRCA, **b** CRC. Significant enrichment or depletion illustrated on red/blue scale, *x*-axis (modules) and *y*-axis (signatures). Hierarchical clustering according to gene signature enrichment (using top 15 signatures per module; FDR <0.05). Scalable version in Supplemental Fig. 11a, b. **c**, **d** Module relationship between cancers analyzed using hypergeometric test displayed as pairwise comparison matrix. Significance of overlap displayed as *p*-values on indicated color scale (*p*-values <0.001); overlap by **c** module gene membership, **d** enriched gene signatures. Gray side bars illustrate maximal significance for module match. Module class Cancer-Hallmark: gray, Cell-of-origin: blue, Chromosome-Region: cyan, Functional-Gene-Battery: yellow, Structural-Gene-Battery: orange and Unassigned: white (Scalable version in Supplemental Fig. 11c, d)

Assessment of network clustering success was based on the enrichment and segregation of gene signatures between the resulting modules. These enrichments (Supplemental Table 3 (BRCA) & 4 (CRC)) were summarized to illustrate the most significantly enriched ontology and signature terms between modules using heatmaps. The purity of segregated biology was reflected in the separation of enriched signatures between individual modules (Fig. 3a, b & Supplemental Fig. 11a, b). A summary designation was assigned to each module based on a selectively enriched term.

We next tested whether recurrent features of cancer biology could be identified in the comparison of modules between the cancer types. Pairwise comparison demonstrated a high degree of similarity at the level of module gene membership (Fig. 3c, Supplemental Fig. 11c). In this gene level comparison, the majority of modules viewed from either cancer perspective had one primary corresponding module, as assessed by the maximal *z*-score for overlap of gene membership, and the majority of modules overall had at least one module with very highly significant overlap. Where a module in one cancer type such as CRC_M17 had multiple highly significantly corresponding modules in BRCA (BRCA_M3, M16, M28, M39, M37), the BRCA modules reciprocally had the CRC module, in this case CRC_M17, as their primary corresponding module. Thus, the modules in the alternate cancer types in some instance reflected a separation into more refined structure. By contrast very few modules generated in either cancer type lacked a significant corresponding module. Given that the network modules are derived in an agnostic fashion from multiple data-sets of entirely disparate cancer types, the level of overlap in gene membership generated by this approach is notable. This supports the argument that these modules of gene co-expression identify recurrent and stable clusters of gene co-expression.

We next extended the analysis to consider the overlap of biology associated with network modules comparing the enrichment of ontologies and signatures associated with each module irrespective of the genes driving that enrichment. This generated a similar but more diverse pattern than that seen at gene level (Fig. 3d, Supplemental Fig. 11d). While most modules retained one or two primary corresponding modules in the alternate cancer type, overall the extent of overlap between modules was both more dispersed and more diverse. The result in part reflects the diversity of gene members in many ontology and signature terms, but additionally points to the fact that while the network modules derived from CRC and BRCA share highly significant core gene membership, they at the same time reflect a greater diversity between the cancer types in terms of the precise patterns of co-expression of genes related to cell function and differentiation.

Considering cancer hallmarks, recurrent modules could be identified relating to pathways linked to cell cycle, immune response, EMT/stroma and angiogenesis. Additional recurrent modules were linked to co-regulated gene batteries such as the IFN-response or growth factor signaling pathways, or structural gene clusters such as Histone, HOX and immunoglobulin genes. Moreover, these modules exhibited shared enrichments for signatures of transcription factor motifs linked to gene promoters (Supplemental Table 5).^21^ In BRCA the impact of chromosomal copy number variation on gene expression in cis has been extensively analyzed.^22^ Such patterns of gene co-expression were recovered in the networks and proved highly reproducible between BRCA and CRC, with the majority of BRCA modules linked to specific chromosomal region having a direct counterpart in CRC (Fig. 3c, d, Supplemental Fig. 11c, d).

Hence, the comparison between cancer types identified principle determinants of gene co-expression patterns. These reflect the impact of cancer hallmarks, functional and structural gene batteries, and copy number variation, which are overlaid on modules linked to the specific biology of the originating cell type.

### Module neighborhoods link to epithelial differentiation pathways

**Fig. 4:**
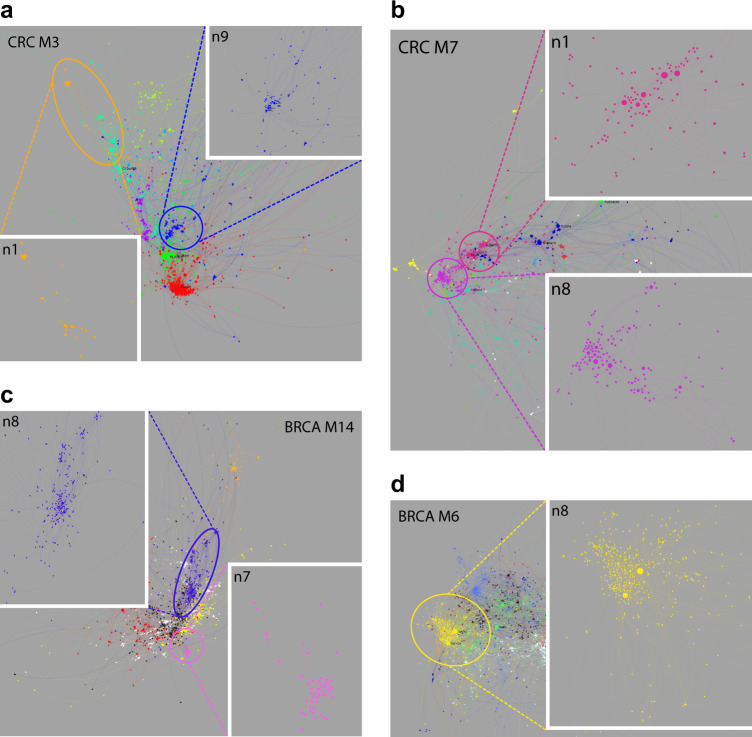
Module neighborhoods provide fine-grained resolution. Neighborhoods within modules are displayed by color code, interactive version online. **a** CRC_M3, enterocyte module, expanded: CRC_M3.n9, WNT-signaling (blue), and CRC_M3.n1, superficial enterocyte (orange). **b** CRC_M7, goblet metabolic module, expanded: CRC_M7.n8, classical goblet cell and CMS3 genes (purple), and CRC_M7.n1 putative deep secretory cell neighborhood (dark pink). **c** BRCA_M14, basal/normal module, expanded: BRCA_M14.n8 (blue), basal classifier genes, and BRCA_M14.n7 (pink), epithelial/epidermal differentiation. **d** BRCA_M6, luminal module, expanded: BRCA_M6.n8, *GATA3* and *ESR1* neighborhood (yellow). Related GSE results in Supplemental Fig. 12

Within the individual modules, the network sub-structure identifies genes with the highest degrees of correlation. To resolve whether these patterns linked to discrete cell states we re-ran the clustering and signature enrichment analysis for module genes independently. We defined the resulting sub-structure as module neighborhoods (Fig. 4, Supplemental Fig. 12, Supplemental Table 6 & online), which illustrates another valuable feature of PGCNA, the scalable nature of the approach.

In CRC features of epithelial differentiation are encompassed in CRC_M3 (enterocyte) and CRC_M7 (goblet cell). The enterocyte module encompassed neighborhoods enriched for genes linked to the WNT-signaling pathway (neighborhood 9, CRC_M3.n9), including *LGR5*,^23^ through to neighborhood CRC_M3.n1 enriched for genes characteristic of the mature enterocyte state (*CA1, CA4, CD177, MS4A12* and *SLC26A3*), recapitulating co-expression observed in single cell analysis of colonic epithelium (Fig. 4a, Supplemental Fig. 12a),^24^ and again illustrating how the application of the network can provide context in complex gene co-expression data. The goblet cell module divides into 11 neighborhoods of which 5 could be assigned to known ontology associations, for example CRC_M7.n10 linked to glycolysis and glucose metabolism and CRC_M7.n11 linked to defense responses (Fig. 4b, Supplemental Fig. 12b). Neighborhood CRC_M7.n8, lacking enrichment of established ontology terms, included the hub genes *FCGBP* and *ST6GALNAC1* as well as *SPINK4* and *MUC2*, that are characteristic goblet cell markers linked to CMS3 CRC.^6,24^ The closely linked neighborhood CRC_M7.n1 included hub genes *REG4*, *AGR2* and *AGR3* (Fig. 4b, online). Notably, REG4 has recently identified as a marker of deep crypt secretory cells.^25^ Using the resolution of the superficial enterocyte cluster in CRC_M3.n1 as reference, this might suggest that genes linked to REG4 in CRC_M7.n1 identify a deep crypt secretory cell program, as opposed to the more generic goblet cell markers in neighborhood CRC_M7.n8.

In BRCA the luminal module (BRCA_M6) divides into nine neighborhoods. Of these BRCA_M6.n8 is enriched for core ESR1 target genes and encompasses *GATA3* and *ESR1* as hub nodes (Fig. 4d, Supplemental Figure 12c, Supplemental Table 6, online).^26,27^ Genes that contribute to a basal-like classification and to epithelial biology fall in BRCA_M14. BRCA_M14.n8 includes the hub gene *SFRP1* as well as *EGFR* and *FOXC1*, PAM50 classifier genes used to define basal breast cancer (Fig. 4c, Supplemental Figure 12d, Supplemental Table 6, online). A subset of basal breast cancer classifier genes are connected to the cytokeratin gene *KRT17* in BRCA_M14.n7 encompassing genes associated with epithelial and epidermal differentiation and linked to normal-like breast cancer classification (Fig. 4c, Supplemental Table 6). Thus, the structure of gene neighborhoods in the epithelial modules reflects patterns of gene expression observed in differentiation, in both CRC and BRCA.

### Networks as multi-layered tools to explore survival associations

**Fig. 5:**
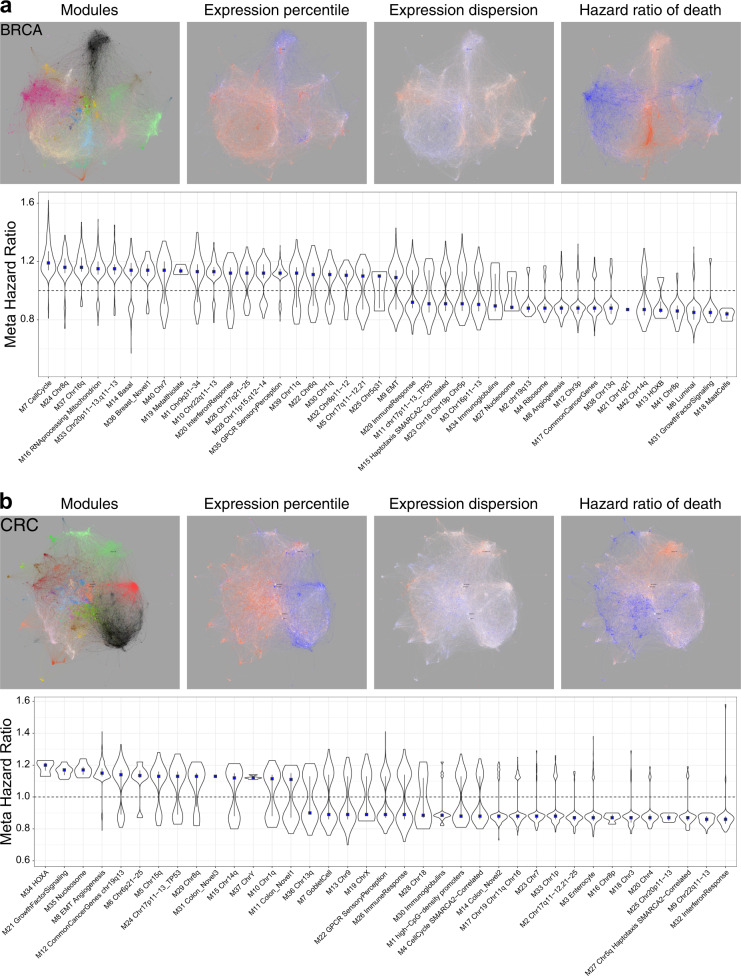
Networks as multilayered tools to explore survival association. BRCA (**a**) and CRC (**b**) meta-information overlay. Left to right: module color code, median expression percentile (relative intensity of expression) across data-sets, median expression dispersion (Quartile coefficient of dispersion, variation between samples/patients) within data-sets, and association of gene expression with meta-analysis Hazard Ratio (HR) of death. Color scales: expression dispersion and variance blue (least) to red (most); outcome blue (low HR—good outcome) to red (high HR—poor outcome). Lower panels ranked module level association with meta HR of death. Distribution of HR associations for module genes with HR *p*-value <0.05, along with median (blue square) and IQR

To provide resources that explore associations of expression with survival, we overlaid meta-information including association of gene expression with hazard ratio (HR) of death (Fig. 5, Supplemental Fig. 13, online). The integration of multiple data sources retained the ability to detect robust HR associations. In the BRCA network, considered without histological subdivision, this recovered the separation of good and adverse outcome between luminal (BRCA_M6) and basal type (BRCA_M14) gene expression (Fig. 5a, Supplemental Fig. 13a). At a module level cell cycle (BRCA_M7) showed the strongest adverse outcome association, which was also evident for modules linked to amplified chromosomal regions that cluster with the cell cycle module (such as BRCA_M24 & M37). Heterogeneity in HR association of module genes, as shown by spread in the violin plot across the neutral line, is a particular feature of the stem cell/EMT (BRCA_M9) and immune response modules (BRCA_M29).

In CRC, the enterocyte (CRC_M3) and interferon response modules (CRC_M32) were linked to good outcome, while adverse outcome associations centered on the EMT/angiogenesis module (CRC_M8) and modules linked to specific chromosomal regions (Fig. 5b, Supplemental Fig. 13b). The three modules with strongest adverse outcome association were HOXA (CRC_M34), growth factor signaling (CRC_M21) (Supplemental Fig. 13c) and nucleosome (CRC_M35). In CRC the immune response module (CRC_M26) also showed a heterogenous pattern, distinct from the near homogenous good outcome association of the IFN module. Thus, mapping of meta-hazard ratios both rediscovers outcome associations linked to the primary modules that map onto existing molecular classification and points to less well appreciated associations. In CRC this is particularly the case for the adverse outcome associations with the structural gene batteries in the HOXA and nucleosome modules, as well as the functional gene battery of growth factor signaling which encompasses genes regulated by one of the most important pathways in this cancer type.

### Network modules provide a platform for molecular stratification

Having validated PGCNA as a tool to interrogate the integrated training data-sets, we next tested the modules as a platform to explore TCGA data.^7,28^ To do this we wished to generate values that would summarize the expression level of a given network module, which could be applied across the entire data-set and on a case by case basis. We used the 25 most representative genes (nodes) of each module to generate module expression values (MEV). The genes contributing to these MEVs were selected from the genes with the highest network connectivity, which provided an indicator of the extent to which a gene was correlated and thus representative of other genes in the network, and focused on genes that are variant across patients, thus most likely to discriminate between patient subsets, but invariant across data-sets, thus least likely to be affected by biases within data-sets. Using these MEVs we first tested the pattern of module expression in the TCGA RNA-seq data by hierarchical clustering at data-set level. In both BRCA and CRC the overall pattern of module co-expression in RNA-seq data was closely related to that in array derived training data-sets (Supplemental Fig. 14). This supported the use of MEVs as a platform to summarize network level gene expression in the TCGA data.

Next applying the MEVs at case/sample level in hierarchical clustering segregated BRCA, initially without considering histological type, into branches according to expression of basal, luminal and mesenchymal related modules. In the latter this distinguished subsets of mesenchymal from mixed mesenchymal/angiogenic BRCA, with the latter including the majority of lobular breast cancers (Supplemental Fig. 15a). Within these major branches further heterogeneity was evident across other network modules, sub-dividing the primary branches according to wider patterns of modular gene expression. Such subdivision was also evident within cases first divided by histological type and then by hierarchical clustering of MEVs (Supplemental Fig. 16a). This for example illustrated a distinctive pattern of MEV expression in mucinous carcinomas with strong luminal and nucleosomal gene expression in the absence of cell cycle, EMT, angiogenesis or immune response signals.

Extending this approach to CRC the clustering divided into three main branches (Supplemental Fig. 15b). The first was characterized by low cell cycle and related chromosomal regional module expression, accompanied by mixed immune and EMT/angiogenesis module expression and subdivision into enterocyte and goblet cell branches; the second enriched for highly mutated cases was characterized by expression of the goblet cell and cell cycle and related modules; the third branch was characterized primarily by high enterocyte module expression. This corresponds broadly to the primary features of the consensus molecular subtypes. However, the expression patterns of the extended network modules illustrated how heterogeneity within these primary branches could be mapped using MEVs to summarize other characteristics of the CRC coexpression network. Such heterogeneity was also evident after separation by mutational load, notably extending to the highly mutated tumor group to identify a subset relatively deficient in immune and EMT/angiogenesis module expression (Supplemental Fig. 16b). Furthermore, differential association of left and right sided tumors with distinct patterns of gene expression was supported by the modular analysis with right sided origin linked to goblet module expression amongst both hypermutated and non-hypermutated subsets, and left sided origin linked to enterocyte module expression irrespective of associated EMT or immune module expression (Supplemental Fig. 16b).

Thus, while the clustering of BRCA and CRC samples based on MEVs reinforces the validity of key features that drive existing paradigms of classifications, the analyses also illustrates that considerable biological heterogeneity is present within such clusters. As a potential solution to assess this heterogeneity the derivation of the PGCNA network and the application of the resulting MEVs can provide a method for summarizing the overall expression state of a cancer sample taking into account key representative genes from across a full network of gene coexpression.

### Data-led network modules have distinctive mutational associations

**Fig. 6:**
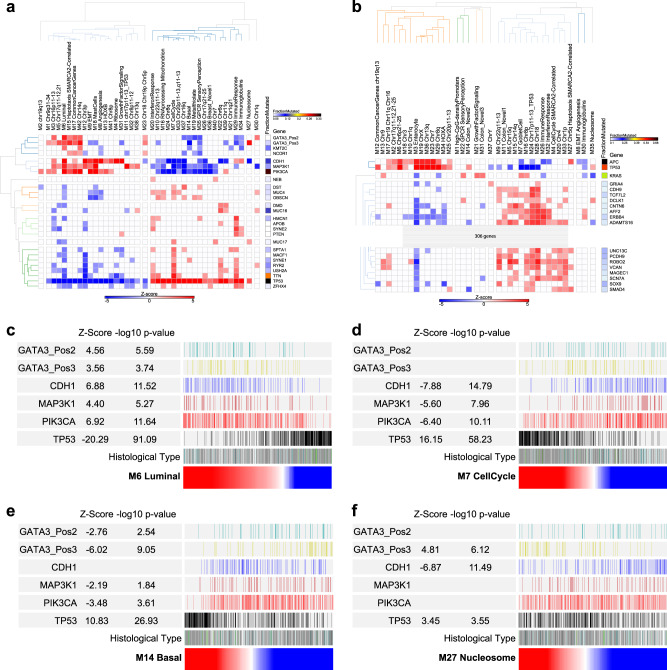
Network modules show distinctive patterns of mutational association. Correlations of MEV with mutation status of commonly mutated genes in TCGA data (**a**) BRCA & (**b**) CRC. Significance of Spearman’s Rank correlation of MEV with mutation, *z*-score on blue to red scale; fraction of mutated cases per gene, blue to black color scale. Hierarchical clustering for genes mutated in ≥5% BRCA and ≥10% CRC of TCGA samples. For CRC the heatmap is truncated for display purposes (complete version Supplemental Fig. 17c). **c** BRCA_M6 Luminal, **d** BRCA_M7 cell cycle, **e** BRCA_M14 basal and **f** BRCA_M27 nucleosome, MEVs as ranking variables (red to blue color scale) for mutation distribution, *z*-score and −log10 *p*-value for *GATA3* with division into proximal (pos2; N-terminus) and distal (pos3; C-terminus), *CDH1*, *MAP3K1*, *PIK3CA* and *TP53*. Histological: ductal (gray), lobular (white) lobular/ductal (dark blue), medullary (green), metaplastic (dark green), mucinous (black), not reported (light blue)

To integrate module expression with gene mutation we first considered BRCA as a single entity. The MEVs provided a summary of module level gene expression across the network which we correlated across the data-set with the presence or absence of mutations in the TCGA data. For BCRA as a whole this demonstrated a primary division of enrichment or anti-enrichment of *TP53* versus *CDH1*, *PIK3CA*, *GATA3*, *MAP3K1*, *KMT2C* and *NCOR1* mutation (Fig. 6a). *TP53* mutation positively correlated with the cell cycle and basal modules, and some chromosomal regional modules. In addition to the cell cycle module the immune response and IFN modules were distinguished by additional positive association with diverse mutational targets. The luminal, EMT, angiogenesis and related modules were significantly anti-correlated with *TP53* mutation and positively associated with combinations of mutations in *CDH1*, *PIK3CA*, *GATA3*, *MAP3K1*, *KMT2C* and *NCOR1*.

*GATA3* mutations can be subdivided between DNA-binding domain or carboxy-terminus, with the latter including frameshift mutations. The potential value of MEVs as a tool for assessing selective patterns of association between gene expression and mutation is illustrated by the observation that *GATA3* mutations affecting the carboxy-terminus are selectively associated with nucleosome module expression. Extending the analysis to BRCA after division by histological subtype, the general pattern observed in all BRCA irrespective of histological type was evident when considering ductal BRCA in isolation (Supplemental Fig. 17a). Lobular BRCA is more molecularly homogenous, reflected in a sparse correlation pattern, nonetheless also retaining features observed in BRCA as a whole (Supplemental Fig. 17b).

For CRC, the pattern was impacted by the high overall mutation load (Fig. 6b, Supplemental Fig. 17c). Associations divided around modules linked with both *TP53* and *APC* mutation and those that correlated with a high mutation load across a wide range of target genes and that were neutral or anti-correlated with *TP53* and *APC* mutation. This separated the enterocyte module, linked to *TP53* and *APC* mutation, and the goblet cell module linked to high mutation load and *KRAS* mutation, with *KRAS* mutation also correlating with the growth factor signaling module. In CRC the cell cycle module was not positively correlated with *TP53* mutation, but instead was linked to the broad swathe of highly mutated target genes. The modules most strongly linked to mutation load encompassed genes from the vicinity of the *TP53* on chr17p, chr18 and components of the immune response and IFN signaling. Overall this reinforces the division of CRC into the major molecular pathways of *TP53* and *APC* mutation versus hypermutational genomic instability and supports the broadly different patterns of molecular features linked to patterns of goblet cell or enterocyte module expression in CRC.

From the point of view of MEVs as a tool for summarizing gene expression across the wider network representative of CRC and BRCA, the pattern of associations observed in the TCGA data supports the validity of this approach. While identifying novel associations, which to our knowledge have not been previously reported such as the linkage between nucleosome gene expression and GATA3 mutation type in BRCA, the analysis recovers many associations that are coherent with known tumor biology.

### Tumor stereotypes with mutation patterns linked to module expression intensity

Next, we addressed the relationship between mutational profile and module expression intensity. A notable feature of this analysis particularly evident for BRCA is the statistically significant association between the intensity of MEV expression and key mutations. Thus, luminal MEV intensity showed a strong positive correlation with *CDH1*, *MAP3K1*, *GATA3* and *PIK3CA* mutations and profound anti-correlation with *TP53* mutation (Fig. 6c). This was paralleled by the opposite association of cell cycle (Fig. 6d) and basal (Fig. 6e) MEV intensity with these mutations. In this context when used as a ranking variable the selective positive correlation of the nucleosome module with *GATA3* 3′-mutations, and anti-correlation with *CDH1* mutation status, was emphasized (Fig. 6f). As part of this specific association, both nucleosome module expression and *GATA3* 3′ mutations were enriched in mucinous BRCA (*p*-value 0.0004). Thus, the use of MEVs as ranking variables illustrates the principle that extremes of module expression associate with increasingly stereotyped tumors and more distinctive patterns of associated mutations, particularly for modules linked to existing expression-based classifications. The potential co-variance of mutation detection and characteristic gene expression points both to stereotypical extremes and centrist cases that concurrently exhibiting less characteristic expression and mutation profiles.

### Selective association of immune neighborhoods with mutational profile in BRCA

**Fig. 7:**
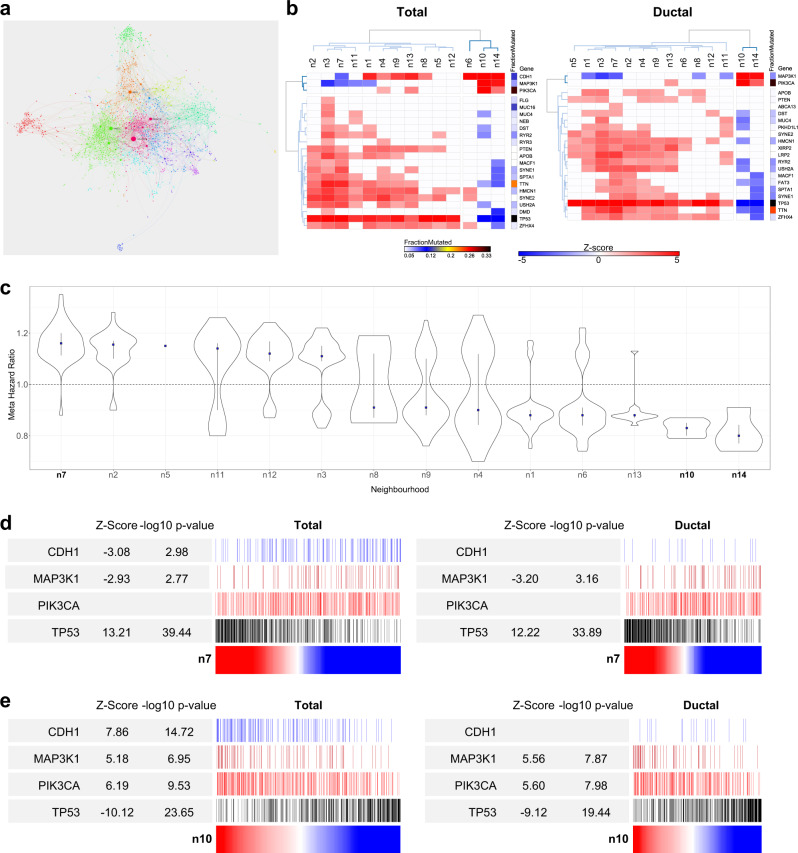
Immune neighborhood analysis. **a** Immune neighborhood analysis for BRCA (across BRCA M18, M20, M29 & M34) displayed as network summary. **b** Significance of Spearman’s Rank correlation of MEV with mutation across immune neighborhoods for all BRCA left and ductal BRCA right; *z*-score on blue to red scale; fraction of mutated cases per gene, blue to black color scale. Hierarchical clustering for genes mutated in ≥5% BRCA of TCGA samples. **c** Ranked neighborhood level association with meta Hazard Ratio (HR) of death. Distribution of HR associations for module genes with HR *p*-value <0.05, along with median (blue square) and IQR. **d**, **e** Use of immune neighborhoods Immune_n7 (**d**) and Immune_n10 (**e**) MEVs as ranking variables (red to blue color scale) for mutation distribution, *z*-score and −log10 *p*-value for *CDH1*, *MAP3K1*, *PIK3CA* and *TP53*: all BRCA left, ductal BRCA right

The interaction between host immune response and molecular subtypes in cancer is of considerable interest. A notable feature of the BRCA analysis was an apparent association of mutational pattern with a module comprised of genes characteristic of mast cells (Fig. 6a and Supplemental Fig. 17a, b). While an association of mast cell infiltration with histological subtypes have been previously described,^29,30^ to our knowledge the selective association of this immune cell subset with underlying mutational state has not been previously noted. Indeed, the discrete association of this cell type specific module appears significantly different from the patterns observed for the wider immune response module in BRCA. To further test the apparent specificity of the mast cell module, we reasoned that the mutational association should remain distinctive when considering the cancer immune response at a network neighborhood level. Focusing on BRCA we therefore integrated network modules linked to immune response features (M18, M20, M29 & M34) and performed a neighborhood analysis. The resulting immune neighborhood network defined 14 modules/neighborhoods of immune response and associated gene expression (Fig. 7a). To test dominant lineage associations in these modules we examined the relative enrichment of gene signatures used in the bioinformatic immune deconvolution approaches, Cibersort and Immunoscore^31,32^ (Supplemental Fig. 18a). This demonstrated the segregation of neighborhoods representing core T-lineage and cytotoxic cell types (Immune_n1), monocytes, macrophage and neutrophils (Immune_n2), M1 macrophages and activated DCs which overlaps with IFN-response linked to MHC class I dependent antigen presentation (Immune_n3), M0 macrophages (Immune_n7), plasma cells and B-cells (Immune_n8), and mast cells (Immune_n10). Thus, this neighborhood analysis identified components of cell lineage specific gene signatures, associated with Cibersort and Immunoscore, that are sufficiently correlated to emerge as distinct modules in BCRA expression data. The remaining network elements add to these lineage-associated neighborhoods to identify coregulated functional gene batteries (Supplemental Fig. 18b). These include the IFN-response, which is sub-divided into a module linked to MHC class-I antigen presentation (Immune_n3) which is IFNγ response biased and overlapping with signatures of M1 macrophages and activated DCs, and other classical IFN-response genes, which are more IFNα response biased (Immune_n11). Other neighborhoods linked to gene batteries are those linked to MHC class-II genes (Immune_n13), a cluster of glutathione-μ genes (Immune_n14), and a neighborhood enriched for components of signal transduction pathways and cytoskeletal reorganization (Immune_n9). Interrogating these immune neighborhoods further illustrated the selective association of immune gene expression with associated mutations, either considering BRCA as a whole or refined to consider ductal carcinoma in isolation (Fig. 7b). Indeed, this analysis confirmed the selective association of mast cell related gene expression Immune_n10 with *CDH1*, *PIK3CA* and *MAP3K1* mutations across BRCA (total data). While the association between mast cell gene expression and *CDH1* mutation was as expected linked to lobular breast cancer, *PIK3CA* and *MAP3K1* mutations were selectively associated with mast cell gene expression when considering only ductal carcinoma. Within this subset mast cell gene expression was significantly anti-correlated with *TP53* mutation. This pattern of associations was shared only with the small set of glutathione-μ genes encompassed in Immune_n14. Other immune gene neighborhoods generally showed positive correlations with *TP53* mutation, and in the case of neighborhoods linked to monocytes and macrophages (n3, n7) as well as MHC class-I dependent antigen presentation (n3), additionally showed anti-correlation with *MAP3K1* mutation state.

Consistent with these mutational and histological associations mast cell and glutathione μ gene expression provided predictors of good outcome in meta-hazard ratio analysis, while adverse outcome was linked to monocyte and macrophage associated modules (Fig. 7c). Finally using mast cell, and macrophage related neighborhood modules as ranking variables emphasized the trend toward stereotyped associations of expression and mutation particularly for *TP53* and *MAP3K1* (Fig. 7d, e). With the intensity of the macrophage related neighborhood Immune_n7 positively correlated with *TP53* and anti-correlated with *MAP3K1*, while the intensity of mast cell related gene expression encompassed in Immune_n10 was strongly positively correlated with *MAP3K1* and PIK3CA and anti-correlated with *TP53*. Thus, this analysis confirms the selective pattern of association that mast cell related gene expression shows with underlying mutational state in BRCA relative to other immune cell type related gene expression profiles. Furthermore, the analysis illustrates how neighborhood analysis using PGCNA can be used to home into consistent patterns of immune gene expression in cancer tissue in a fashion that complements approaches based on pre-defined immune subset signatures derived from purified cell subsets.

## Discussion

We set out to test whether the modular nature of gene co-expression could be used to derive expression codes summarizing diverse features of cancer biology, and whether this could be generated from a network level view of gene co-expression using BRCA and CRC as exemplars. These two common cancers were selected as representative of well-defined pre-existing molecular classifications and extensive expression resources. Amongst the features we considered desirable to achieve, were that the networks should generate a structure that contextualizes patterns of gene co-expression across all genes used in module generation, and that the resolved modules of genes should identify meaningful biology within and between cancer types. Furthermore, we reasoned that by using an unsupervised approach we would be able to test the recovery of pre-existing paradigms, and that the resulting modules should provide the potential basis for summarizing gene expression across the network in individual samples.

A major challenge to achieving this aim lay in finding an approach for edge reduction that would allow the highly complex gene co-expression data to be reduced in complexity while retaining information that allowed effective clustering. A striking finding of the analysis was that the radical pruning of edges in expression correlation matrices, retaining only the genes most significantly correlated with any individual index gene, prior to network analysis with the approach know as Fast Unfolding Communities in Large Networks (referred to in this work as FastUnfold), allowed remarkably efficient recovery of biology. This result was not intuitive, indeed a striking feature of the methodological development is that such edge reduction greatly enhances the performances of the FastUnfold algorithm, which when working on the total data-set performs significantly less well. Indeed, the EPG3 approach gave superior results to other data filtering methods tested—iPCC, power function or the sigmoid function of weighted gene correlation network analysis (WGCNA)—as assessed by a combination of module number and gene distribution, biological enrichment, and percentage of genes connected in the network. Interestingly, across the range of edge reduction thresholds tested, below ten edges per gene, the resultant parsimonious matrices yielded topologically scale-free networks. Furthermore, in comparison with WGCNA, the combination of parsimonious correlation matrices and FastUnfold produced a greater consistency of module composition and a greater resolution of gene signatures and ontology terms. We note that WGCNA is widely used and a highly effective tool for analysis of expression data-sets but is not optimized to deal with correlation matrices derived from multiple merged expression data-sets. We would note in this context that PGCNA can also be used to effectively explore smaller sets of experimental data, and we have provided an example of this in a companion paper applying the method described here in experimental time course data related to cellular differentiation.^33^

A further feature of the PGCNA approach is the computational efficiency and scalable nature of the method. Firstly, this allows effective network analysis with generally available computers without dependence on a high-performance computing cluster. Secondly the method can be scaled, both up to allow analysis of very large data-sets, and down to provide a focused analysis of subsets of genes within a wider network. The latter we refer to as a neighborhood analysis and allows resolution of a substructure such as patterns of gene co-expression within the luminal module of CRC, including resolution of gene co-expression clusters seen previously in single-cell analysis of cellular sub-populations,^24^ or on the other hand in the resolution of reproducible co-expressed gene clusters of cancer associated immune responses.^12,13,31,32^ Thus, the network and its modular structure may be used at different levels to separate or coalesce cellular features.

CRC and BRCA show a remarkable communality in gene co-expression patterns consistent with a core set of principles that underpin patterns of co-expression in cancer. These can be summarized as (1) genes linked to cancer hallmark features; (2) functional gene batteries linked to either specific pathways such as the IFN-response or growth factor receptor signaling or to structural clusters of co-regulated genes; and (3) to co-expression related to copy number variation. In each case these shared drivers are associated in the networks with modules derived from the selective biology of the originating lineage. While this pattern is consistent with prevailing paradigms, both of the nature of cancer hallmarks and of the impact of cis-acting modules derived from structural genomic abnormalities,^22^ an important feature provided by PGCNA is the fact that such gene expression patterns are placed in context. One of the features we aimed for in the network analysis was that the process of edge reduction should both generate more visually accessible networks, and at the same time allow all elements of the expression data used in the module generation to be visualized. The importance of this is that the resulting networks provide context for how genes within and between modules are linked. The networks as an interactive resource summarize the co-expression patterns of genes and modules of genes as a searchable map rather than as disconnected lists or heatmap representations. While many genes retain only few edges in these networks, due to the nature of the edge reduction technique, all the data for each gene is provided as a fully accessible resource providing the correlation data for all gene pairs across the entire sets of expression data used for network generation.

As a platform from which to enrich molecular stratification, the networks recover modules that map closely onto existing classifications for both BRCA and CRC, and place these in a wider context. Using hub genes to generate MEVs allowed the integration of expression with mutation profiles in the TCGA resource at data-set and case-by-case level, in effect exploring the TCGA data from the perspective of the deep expression data available for BRCA and CRC. Together these provide evidence that molecular classification may be enriched by using MEVs as a gene expression barcode. The clustering of samples based on MEVs is consistent with the existing paradigms of expression-based classifications for these cancers. However, the analyses illustrate heterogeneity within clusters of cases that map onto these classifications, when considering wider patterns of gene expression. In the context of molecular classification and precision oncology there are competing goals of assigning patients to discrete classes versus characterizing bar codes of features that capture the wider genomic and expression features of an individual cancer. As a potential solution to this issue the derivation of the PGCNA network and the application of the resulting MEVs could provide a method for summarizing the overall expression state of a cancer sample. These MEVs take into account key representative genes from across the entire network of gene expression for that cancer type. Moreover, these are derived in a data-led agnostic fashion from across multiple openly accessible expression resources and allow comparison of informative genes between cancer types. The MEVs while utilizing many more genes than are currently encompassed in most expression-based classifiers, still remain within the range that could be encompassed within focused gene expression platforms that meet regulatory standards.

In comparison to previous studies of TCGA consortium, and of the TCGA data by others, the primary difference lies in the application of PGCNA and the resulting network modules. In this context the rediscovery of particular associations between mutation state and expression as assessed with MEVs provides part of the validation of their utility. The analysis of TCGA data with MEVs illustrates how the summary of network level expression patterns at individual sample level can highlight heterogeneity of expression within clusters such as the expression variation within hypermutated CRC. Furthermore, the use of MEVs as ranking variables illustrates the occurrence of stereotypical associations of mutation and expression, this also illustrates the ability to identify previously unanticipated linkages, at least to our knowledge, such as that of nucleosome module expression with *GATA3* mutation type in BRCA. Mast cell related gene expression in the immune neighborhood analysis provides another example, the association particularly in the ductal subset with *MAP3K1* and *PIK3CA* mutation and absence of *TP53* mutation is distinctive relative to other immune cell types. This raises interesting questions as to why this particular immunological cell type should have such distinct associations with underlying molecular pathology.

The approach we describe here has both disease-specific and general relevance. It provides an approach for extracting useful networks that can be applied effectively to diverse clinical and experimental data-sets, while also generating a mineable resource, and illustrates how resulting network modules might be used to sit alongside existing expression-based classifications to enhance molecular stratification.

## Methods

### Method details

See Supplemental Fig. 1 for outline, will refer to numbers in this figure in the sections below. For a more detailed method discussion, along with the motivations for developing PGCNA, see the Supplemental Methods document (Supplemental Materials page 19 onwards), for details of software versions and data-sets see Table 1.

**Table 1:** Key resources table

Resource	Source	Identifier
*Deposited data*		
BRCA expression data 1	GEO	GSE12276
BRCA expression data 2	GEO	GSE16201
BRCA expression data 3	GEO	GSE20194
BRCA expression data 4	GEO	GSE20271
BRCA expression data 5	GEO	GSE20685
BRCA expression data 6	GEO	GSE21653
BRCA expression data 7	GEO	GSE22093
BRCA expression data 8	GEO	GSE22219
BRCA expression data 9	GEO	GSE22820
BRCA expression data 10	GEO	GSE24450
BRCA expression data 11	GEO	GSE25066
BRCA expression data 12	GEO	GSE25307
BRCA expression data 13	GEO	GSE26639
BRCA expression data 14	GEO	GSE2990
BRCA expression data 15	GEO	GSE30682
BRCA expression data 16	GEO	GSE31448
BRCA expression data 17	GEO	GSE3494
BRCA expression data 18	GEO	GSE36774
BRCA expression data 19	GEO	GSE45725
BRCA expression data 20	GEO	GSE4611
BRCA expression data 21	GEO	GSE48091
BRCA expression data 22	GEO	GSE4922
BRCA expression data 23	GEO	GSE54002
CRC expression data 1	GEO	GSE17536
CRC expression data 2	GEO	GSE17537
CRC expression data 3	GEO	GSE26682
CRC expression data 4	GEO	GSE26682
CRC expression data 5	GEO	GSE28722
CRC expression data 6	GEO	GSE37892
CRC expression data 7	GEO	GSE38832
CRC expression data 8	GEO	GSE39582
CRC expression data 9	GEO	GSE42284
CRC expression data 10	GEO	GSE44076
CRC expression data 11	GEO	GSE5206
CRC expression data 12	GEO	GSE68468
BRCA TCGA RNA-seq	TCGA Consortium	https://portal.gdc.cancer.gov/projects/TCGA-BRCA
BRCA TCGA Mutation	TCGA Consortium	https://portal.gdc.cancer.gov/projects/TCGA-BRCA
CRC TCGA RNA-seq	TCGA Consortium	https://portal.gdc.cancer.gov/projects/TCGA-COAD
CRC TCGA Mutation	TCGA Consortium	https://portal.gdc.cancer.gov/projects/TCGA-COAD
Software and Algorithms		
Fast unfolding of communities in large networks (v0.3)	Blondel et al.^19^	https://sourceforge.net/projects/louvain/?source=navbar
GENE-E (v 3.0.21)	N/A	https://software.broadinstitute.org/GENE-E/
Gephi (v0.82)	Bastian et al.^69^	https://gephi.org/
Gephi sigma.js library	N/A	https://github.com/oxfordinternetinstitute/gephi-plugins/tree/sigmaexporter-plugin
MyGene.info	Xin et al.^66^	http://mygene.info
Python 2.7.1	N/A	https://www.anaconda.com/distribution/
R fastcluster package (v1.1.24)	Müllner^68^	https://cran.r-project.org/web/packages/fastcluster/index.html
R Limma package (v3.34.9)	Ritchie et al.^67^	http://bioconductor.org/packages/release/bioc/html/limma.html
R metafor package (v2.0.0)	Viechtbauer^71^	https://cran.r-project.org/web/packages/metafor/index.html
WGCNA (v1.66)	Langfelder and Horvath^15^	https://horvath.genetics.ucla.edu/html/CoexpressionNetwork/Rpackages/WGCNA/

### Expression data-sets

See Supplemental Fig. 1 part 1

For the generation of the gene correlation networks 23 breast cancer (BRCA) and 12 colorectal cancer (CRC) gene expression data-sets were downloaded from the Gene Expression Omnibus (BRCA, 7464 cases; 26 arrays)^34–55^ and (CRC, 2399 cases; 11 arrays after merging of 2).^56–65^ Three of the BRCA data-sets were on two different expression platforms (GSE3494, GSE36774 and GSE4922), these were analyzed independently, giving a total of 26 BRCA expression data-sets. In the case of CRC two related data-sets were merged (GSE17536), giving a total of 11 CRC data-sets.

#### TCGA data-sets

For independent assessment of the network modules two RNA-seq data-sets were downloaded from The Cancer Genome Atlas (BRCA/CRC data-sets were downloaded on 2017.11.15 from http://cancergenome.nih.gov/) along with the corresponding simple nucleotide variation data (MuTect2 pipeline). The overlapping expression/mutation samples were used for downstream analyses.

#### Normalization and re-annotation of data

For each data-set the probes were re-annotated using the MyGene.info (http://mygene.info) API using all available references (e.g. NCBI Entrez, Ensembl etc.) and any ambiguous mappings manually assigned.^66^

Each data-set was quantile normalized using the R Limma package and the probes for each gene merged by taking the median value for probe sets with a Pearson correlation ≥0.2 and the maximum value for those with a correlation <0.2.^67^

### Network analysis

This discusses how the Parsimonious Gene Correlation Network Analysis (PGCNA) approach was developed.

#### Gene correlation calculation

See Supplemental Fig. 1 part 2 and 3

For each expression data-set the 80% most variant genes were used to calculate Spearman’s rank correlations for all gene pairs using the Python scipy.stats package. The resultant *p*-values and correlations matrices were merged across all data-sets for a given cancer by taking the median values (across the sets in which the gene pairs were contained) to give a final median correlation matrix and its corresponding *p*-value matrix. Genes present in <9 data-sets for BRCA and <4 data-sets for CRC were removed from respective matrices. This gave a final matrix size of 17,805 and 18,896 for BRCA and CRC, respectively. Finally, all correlations with a *p*-value >0.05 were set to 0 to reduce noise.

#### Edge reduction

See Supplemental Fig. 1 part 4

We tested a simple but aggressive edge reduction strategy as a way to improve module discovery and network visualization. For each gene (row) in a correlation matrix only the *N* most correlated Edges Per Gene (EPG) were retained, with *N* ranging from 3 to 10 (<3 gives orphan modules). The resulting matrix *M*, with entries written as *M* = (*m*_*ij*_) was made symmetrical by setting *m*_*ij*_ = *m*_*ji*_ for all indices *i* and *j* so that *M* = M^T^ (its transpose). For EPG3 this reduced the nodes in BRCA from 43,231,589 to 49,199 and CRC from 42,142,502 to 52,257, in both cases >800-fold reduction (Supplemental Table 1).

#### Data clustering

See Supplemental Fig. 1 part 5

The matrices from the edge reduction step alongside the Total matrices were clustered using three different approaches: hierarchical clustering using the R package fastcluster, k-means clustering using the R package kmeans and a network level clustering using the Fast unfolding of communities in large networks algorithm (version 0.3) referred to subsequently herein as FastUnfold.^19,68^ FastUnfold was run 10,000 times at each EPG level and the 100 best (judged by the modularity score) were used for downstream analysis. The FastUnfold algorithm automatically converges on a module number and therefore does not require a user defined module number.

For the k-means clustering k was set to ±1 around the module number from the best FastUnfold solution (see Cluster selection) and for each k and EPG 50 iterations were run.

For hierarchical clustering eight different linkage methods (average, centroid, complete, Mcquitty, Median, Single, WardD and WardD2) were used and the resultant dendrograms cut at ±10 around the module number from the best FastUnfold solution giving 21 results for every input matrix (note: only the 2 best linkage methods, WardD/WardD2, are shown in Fig. 1a).

#### Comparison of edge refinement approach

As well as edge-per-gene (EPG), three other edge refinement approaches were tested: iterative Pearson’s Correlation coefficient (iPCC) and the two WGCNA adjacency approaches power/sigmoid, for a detailed discussion of this see the Supplemental Methods document.

#### Comparison of edge refinement and with WGCNA

In addition to comparing the PGCNA method against other clustering methods (see Data clustering) we validated it against the popular WGCNA gene network analysis tool. For a detailed discussion of this see the Supplemental Methods document.

#### Cluster selection

See Supplemental Fig. 1 part 6

The success of the clustering approaches was assessed by looking at the level of biological enrichment of each module while rewarding purity (biological enrichment in single modules) and similar (even) module sizes (i.e. to avoid skewing to a few modules that contain many genes/functions).

Gene signature analysis was carried out for each module, from each clustering of the data. Then to generate a total enrichment score for a given clustering:

Signatures were filtered to retain only those with ≥5 and ≤1000 genes with an FDR (Benjamini Hochberg) of <0.05.

For each module within a clustering, the enriched signatures were ranked by FDR and the top 15 added to a global list of signatures for that clustering.

A matrix was generated that contained all the *z*-scores for every signature (rows) in the global list across all the modules (columns).

For each signature a fractional contribution was calculated as the row-max-zScore/row-sum-zScores (where 1=enrichment of signature in only 1 module). Across all signatures a median factional contribution (MFC) was calculated.

The sum of the maximum *z*-score per signature (row) was calculated (ZScoreMS).

Module size skewing was assessed by calculating the normalized Shannon entropy:

$${\boldsymbol{H}}_{\boldsymbol{n}}\left( {\boldsymbol{p}} \right) = - \mathop {\sum }\limits_{\boldsymbol{i}} \frac{{{\boldsymbol{p}}_{\boldsymbol{i}}\,\log _{\boldsymbol{b}}{\boldsymbol{p}}_{\boldsymbol{i}}}}{{\log _{\boldsymbol{b}}{\boldsymbol{n}}}}$$

of the module sizes. This gave a score that ranged from 1 (even module sizes) towards 0 with increasing skewing.

A Scaled cluster enrichment score (SCES)=ZScoreMS⋅MFC⋅normalizedEntropy. This allowed the selection of the best FastUnfold clustering (Fig. 1a; Gene Signature Enrichment: FastUnfold). This was then used to set the module number range in the k-means/hierarchical approaches. The FastUnfold method outperformed the k-means/hierarchical clustering methods across all EPG, with only the Ward-linkage hierarchical clustering approaching a similar enrichment when using the Total data. With increasing EPG there was a corresponding decrease in module number with no trade-off of increased biological enrichment (Supplemental Fig. 2a and Fig. 1a). Thus, for all downstream analysis we chose the optimal FastUnfold EPG3 result for both cancers. However, it should be noted that most of the recovered modules were broadly retained across the 100 FastUnfold clustering results (see Module Stability and Supplemental Fig. 2b, c). The combination of FastUnfold and EPG3 we term a Parsimonious Gene Correlation Network Analysis (PGCNA).

Figure 3a, b and Supplemental Fig. 11a, b show visualizations of the optimal BRCA/CRC gene signature results. As before these show the top 15 signatures per module (with ≥5 and ≤1000 genes) but are filtered with the more lenient *p*-value <0.01.

#### Module stability

The stability of modules was assessed to see how recurrent the modules were across different clustering runs (Supplemental Fig. 2b, c). Using the optimal clustering as a reference, for each of the 100 FastUnfold clustering, per reference module:

Find the maximum overlapping module.

Store the number of overlapping genes along with significance (*p*-value) of the overlap and increment sums for the overlapping genes.

The stability percentage per gene is simply the overlap sum (i.e. across 100 clustering runs what percentage of maximum overlapping modules is the gene found in). The stability values per reference module were calculated as median overlap across the 100 clustering runs.

#### Network visualization

See Supplemental Fig. 1 part 7

The optimal EPG3 matrix from BRCA/CRC was converted into a list of edges and nodes and uploaded into the Gephi package (version 0.82).^69^ Modules were colored so that where possible significantly overlapping modules between BRCA and CRC shared colors. Degree and Betweenness Centrality were calculated and the latter used to adjust node sizes. The network layout was generated using the ForceAtlas2 approach,^70^ and interactive HTML5 web visualizations exported using the sigma.js library (https://github.com/oxfordinternetinstitute/gephi-plugins/tree/sigmaexporter-plugin).

#### Network meta-data

See Supplemental Fig. 1 part 8

A number of additional features were calculated for the network genes across the data-sets used to generate the correlation network. For each gene the median percentile expression was calculated across all data-sets, its dispersion across data-sets calculated as the median absolute deviation (MAD) and its dispersion within data-sets (i.e. across patients) calculated as the median quantile coefficient of dispersion (QCOD).

The Survival library for R was used to analyze right-censored survival data for the data-sets where this was available (*n* = 8 for BRCA, *n* = 4 for CRC). Within each data-set the expression of each gene (as *z*-score) was used as a continuous variable in a Cox Proportional Hazards model. Across data-sets a meta-analysis was conducted by fitting a fixed-effect model (R metafor package) to the hazard ratios, weighted by data-set size.

rma(yi=lnHazardRatio,sei=standardErr,weights=dataSetSize,weighted=TRUE,method=“FE”).^71^

#### Module overlaps

See Supplemental Fig. 1 part 9

The overlap of the modules between the cancers at the gene and signature level was assessed using a hypergeometric test and the overlap visualized as a python matplotlib heatmap of −log_10_ *p-*values (Fig. 3c, d), and with the overlap number and module size displayed (Supplemental Fig. 11c, d). The signatures were pre-filtered to *p*-value <0.001 and ≥ 5 and ≤ 1000 genes.

### Application to TCGA data

The modules derived from the GEO ‘training data’ were used to analyze unseen expression and mutation data from The Cancer Genome Atlas (TCGA).

#### Module Expression Values

See Supplemental Fig. 1 part 10

To assign module enrichment/depletion at the patient level a summary score was created for each module for each patient.

Within each data-set, which vary in available genes, the first step was to select the 25 most representative genes per module:

For every gene a connectivity score was calculated by summing its correlations within its module. This was then weighted using expression and dispersion information

$${\mathrm{ModCon}} = {\mathrm{connectivity}}^2 \cdot {\mathrm{percentileExpression}} \cdot {\mathrm{VarWithin}} \cdot \left( {100-{\mathrm{VarAcross}}} \right){\mathrm{/}}100$$

Where VarWithin is the dispersion of a gene expression within data-sets measured as the median quantile coefficient of dispersion (max range 0–1), VarAcross is the dispersion of gene expression across data-sets measured as the median absolute deviation of percentile expression (max range 0–100). This rewards genes that have high connectivity and are variant across patients but invariant across data-sets.

Genes were ranked by ModCon and the top 25 selected. These 25 genes were then converted to a Module Expression Value (MEV): Per gene, standardize (*z*-score) the quantile normalized log_2_ expression data. Per sample (patient) sum the 25 *z*-scores to give a MEV.

#### Heatmap visualizations

See Supplemental Fig. 1 part 11

The MEV were used to create heatmap visualizations of each module at the patient level within the BRCA and CRC TCGA data-sets. Using the Broad GENE-E package (https://software.broadinstitute.org/GENE-E/) the MEV were hierarchically clustered (Pearson correlations and average linkage) and displayed along with available meta data (Supplemental Figs 15 and 16).

#### Mutation correlation analysis

See Supplemental Fig. 1 part 12

The relationship of mutations and modules was calculated using the MuTect2 simple nucleotide variation (SNV) mutation data and the MEV. The SNV data was filtered to retain mutations present in >5 or >10% of patients in BRCA and CRC respectively. Spearman’s rank correlations were calculated between all pairs of mutated gene and module. These were converted to *z*-scores to convey the ±correlation along with its significance. A matrix was output containing the *z*-scores for all gene/modules ≥1 positive significant (*p*-value <0.05) correlation (i.e. a gene need only be significant in one module to be included). This matrix was then hierarchically clustered (Pearson correlations and average linkage) using GENE-E (Figs 6 and 7 and Supplemental Fig. 17).

For BRCA the 140 GATA3 mutations were split into three groups based on mutation position: GATA3_Pos1 (Chr10: 8058419–8064131; *n* = 10), GATA3_Pos2 (Chr10: 8069470–8069596; *n* = 57) and GATA3_Pos3 (Chr10: 8073734–8074229; *n* = 73).

### Quantification and statistical analysis

#### Gene signature data and enrichment analysis

A data-set of 17,211 gene signatures was created by merging signatures downloaded from http://lymphochip.nih.gov/signaturedb/ (SignatureDB), http://www.broadinstitute.org/gsea/msigdb/index.jsp MSigDB V6.1 (MSigDB C1–C7 and H; excluding C5. With MIPS signatures from version 3.1 and PID signatures from version 4 added back), http://compbio.dfci.harvard.edu/genesigdb/ Gene Signature Database V4 (GeneSigDB), UniProt keywords (parsed XML from http://www.uniprot.org/downloads), and locally curated lists. A gene ontology gene set was created using an in-house python script. This parses a gene association file (http://geneontology.org/page/download-go-annotations) to link genes with ontology terms and then uses the ontology structure (.obo file; http://purl.obolibrary.org/obo/go.obo) to propagate these terms up to the root. The resultant gene set contained 22,271 terms. The gene-ontology and gene-signatures sets were merged to give a final signature set of 39,482 terms.

Enrichment of gene lists for signatures was assessed using a hypergeometric test, in which the draw is the gene list genes, the successes are the signature genes, and the population is the genes present on the platform.

#### Correlation of modules

The relationship of the modules was analyzed by calculating the Spearman’s rank correlation for all module (as MEV) pairs within each data-set. These were then merged across data-sets by calculating the median correlation and *p*-values. A final matrix generated by setting all correlations with a *p*-value >0.05 to 0. Within GENE-E the ‘training data’ was hierarchically clustered (Pearson correlations and average linkage) and the TCGA data displayed in the same order without hierarchical clustering (Supplemental Fig. 14).

### Data and software availability

Interactive networks and all meta-data are available at http://pgcna.gets-it.net/. PGCNA python scripts are available at https://bitbucket.org/mcare/pythonscripts-pgcna/src. The PGCNA python program has been implemented to store data using the HDF5 data format. This allows a very small memory footprint (<1GB) even when analyzing very large data-sets. For example, running a 20,000 gene data-set (3GB correlation data-file) with 1000 clusterings takes ~20 min, however, even an 180,000 node data-set (240 GB correlation data-file) can be run in <1 GB or RAM, taking ~13 h to run (including 1000 FastUnfold clusterings). This large data-set is reduced from 16 billion edges to 540,000 (43MB), which is possible to render using Gephi V0.9.2.

### Reporting Summary

Further information on experimental design is available in the Nature Research Reporting Summary linked to this article.

## Supplementary information

Reporting Summary

Supplemental Materials

Supplemental Table 1

Supplemental Table 2

Supplemental Table 3

Supplemental Table 4

Supplemental Table 5

Supplemental Table 6

## Supplementary information

**Supplementary Information** accompanies the paper on the *npj Systems Biology and Applications* website (10.1038/s41540-019-0090-7).

## References

[1] Kan Z (2010). Diverse somatic mutation patterns and pathway alterations in human cancers. Nature.

[2] Hoadley KA (2018). Cell-of-origin patterns dominate the molecular classification of 10,000 tumors from 33 types of cancer. Cell.

[3] Alizadeh AA (2000). Distinct types of diffuse large B-cell lymphoma identified by gene expression profiling. Nature.

[4] Perou CM (2000). Molecular portraits of human breast tumours. Nature.

[5] Sorlie T (2001). Gene expression patterns of breast carcinomas distinguish tumor subclasses with clinical implications. Proc. Natl Acad. Sci. USA.

[6] Guinney J (2015). The consensus molecular subtypes of colorectal cancer. Nat. Med..

[7] Ciriello G (2015). Comprehensive molecular portraits of invasive lobular breast cancer. Cell.

[8] Parker JS (2009). Supervised risk predictor of breast cancer based on intrinsic subtypes. J. Clin. Oncol..

[9] Scott DW (2014). Determining cell-of-origin subtypes of diffuse large B-cell lymphoma using gene expression in formalin-fixed paraffin-embedded tissue. Blood.

[10] Hanahan D, Weinberg RA (2011). Hallmarks of cancer: the next generation. Cell.

[11] Stuart JM, Segal E, Koller D, Kim SK (2003). A gene-coexpression network for global discovery of conserved genetic modules. Science.

[12] Galon J (2006). Type, density, and location of immune cells within human colorectal tumors predict clinical outcome. Science.

[13] Gentles AJ (2015). The prognostic landscape of genes and infiltrating immune cells across human cancers. Nat. Med..

[14] Margolin AA (2006). ARACNE: an algorithm for the reconstruction of gene regulatory networks in a mammalian cellular context. BMC Bioinform..

[15] Langfelder P, Horvath S (2008). WGCNA: an R package for weighted correlation network analysis. BMC Bioinform..

[16] Zhang X (2013). NARROMI: a noise and redundancy reduction technique improves accuracy of gene regulatory network inference. Bioinformatics.

[17] Roy S (2013). Integrated module and gene-specific regulatory inference implicates upstream signaling networks. PLoS Comput. Biol..

[18] Horvath S (2006). Analysis of oncogenic signaling networks in glioblastoma identifies ASPM as a molecular target. Proc. Natl Acad. Sci. USA.

[19] Blondel, V. D., Guillaume, J. L., Lambiotte, R. & Lefebvre, E. Fast unfolding of communities in large networks. *J. Stat. Mech. Theory Exp*. Artn P10008 10.1088/1742-5468/2008/10/P10008 (2008).

[20] Ren X, Wang Y, Zhang XS, Jin Q (2013). iPcc: a novel feature extraction method for accurate disease class discovery and prediction. Nucleic Acids Res..

[21] Xie X (2007). Systematic discovery of regulatory motifs in conserved regions of the human genome, including thousands of CTCF insulator sites. Proc. Natl Acad. Sci. USA.

[22] Curtis C (2012). The genomic and transcriptomic architecture of 2,000 breast tumours reveals novel subgroups. Nature.

[23] Barker N (2007). Identification of stem cells in small intestine and colon by marker gene Lgr5. Nature.

[24] Dalerba P (2011). Single-cell dissection of transcriptional heterogeneity in human colon tumors. Nat. Biotechnol..

[25] Sasaki N (2016). Reg4+ deep crypt secretory cells function as epithelial niche for Lgr5+ stem cells in colon. Proc. Natl Acad. Sci. USA.

[26] Theodorou V, Stark R, Menon S, Carroll JS (2013). GATA3 acts upstream of FOXA1 in mediating ESR1 binding by shaping enhancer accessibility. Genome Res..

[27] Kouros-Mehr H, Slorach EM, Sternlicht MD, Werb Z (2006). GATA-3 maintains the differentiation of the luminal cell fate in the mammary gland. Cell.

[28] Atlas N (2012). Comprehensive molecular characterization of human colon and rectal cancer. Nature.

[29] Du T (2018). Invasive lobular and ductal breast carcinoma differ in immune response, protein translation efficiency and metabolism. Sci. Rep..

[30] Aponte-Lopez A, Fuentes-Panana EM, Cortes-Munoz D, Munoz-Cruz S (2018). Mast cell, the neglected member of the tumor microenvironment: role in breast cancer. J. Immunol. Res..

[31] Newman AM (2015). Robust enumeration of cell subsets from tissue expression profiles. Nat. Methods.

[32] Bindea G (2013). Spatiotemporal dynamics of intratumoral immune cells reveal the immune landscape in human cancer. Immunity.

[33] Stephenson S (2019). Growth factor-like gene regulation is separable from survival and maturation in antibody-secreting cells. J. Immunol..

[34] Bos PD (2009). Genes that mediate breast cancer metastasis to the brain. Nature.

[35] Roepman P (2009). A gene expression profile for detection of sufficient tumour cells in breast tumour tissue: microarray diagnosis eligibility. BMC Med. Genomics.

[36] Popovici V (2010). Effect of training-sample size and classification difficulty on the accuracy of genomic predictors. Breast Cancer Res..

[37] Tabchy A (2010). Evaluation of a 30-gene paclitaxel, fluorouracil, doxorubicin, and cyclophosphamide chemotherapy response predictor in a multicenter randomized trial in breast cancer. Clin. Cancer Res..

[38] Kao KJ, Chang KM, Hsu HC, Huang AT (2011). Correlation of microarray-based breast cancer molecular subtypes and clinical outcomes: implications for treatment optimization. BMC Cancer.

[39] Sabatier R (2011). A gene expression signature identifies two prognostic subgroups of basal breast cancer. Breast Cancer Res. Treat..

[40] Iwamoto T (2011). Gene pathways associated with prognosis and chemotherapy sensitivity in molecular subtypes of breast cancer. J. Natl Cancer Inst..

[41] Buffa FM (2011). microRNA-associated progression pathways and potential therapeutic targets identified by integrated mRNA and microRNA expression profiling in breast cancer. Cancer Res..

[42] Cunha SI (2015). Endothelial ALK1 is a therapeutic target to block metastatic dissemination of breast cancer. Cancer Res..

[43] Wang DY, Done SJ, Mc Cready DR, Leong WL (2014). Validation of the prognostic gene portfolio, ClinicoMolecular Triad Classification, using an independent prospective breast cancer cohort and external patient populations. Breast Cancer Res..

[44] Servant N (2012). Search for a gene expression signature of breast cancer local recurrence in young women. Clin. Cancer Res..

[45] Jonsson G (2012). The retinoblastoma gene undergoes rearrangements in BRCA1-deficient basal-like breast cancer. Cancer Res..

[46] Sabatier R (2011). Down-regulation of ECRG4, a candidate tumor suppressor gene, in human breast cancer. PLoS ONE.

[47] Liu RZ (2011). Association of FABP5 expression with poor survival in triple-negative breast cancer: implication for retinoic acid therapy. Am. J. Pathol..

[48] Heikkinen T (2011). Variants on the promoter region of PTEN affect breast cancer progression and patient survival. Breast Cancer Res..

[49] Hatzis C (2011). A genomic predictor of response and survival following taxane-anthracycline chemotherapy for invasive breast cancer. J. Am. Med. Assoc..

[50] de Cremoux P (2011). Importance of pre-analytical steps for transcriptome and RT-qPCR analyses in the context of the phase II randomised multicentre trial REMAGUS02 of neoadjuvant chemotherapy in breast cancer patients. BMC Cancer.

[51] Sotiriou C (2006). Gene expression profiling in breast cancer: understanding the molecular basis of histologic grade to improve prognosis. J. Natl Cancer Inst..

[52] Ivshina AV (2006). Genetic reclassification of histologic grade delineates new clinical subtypes of breast cancer. Cancer Res..

[53] Miller LD (2005). An expression signature for p53 status in human breast cancer predicts mutation status, transcriptional effects, and patient survival. Proc. Natl Acad. Sci. USA.

[54] Karn T (2010). Data-driven derivation of cutoffs from a pool of 3,030 Affymetrix arrays to stratify distinct clinical types of breast cancer. Breast Cancer Res. Treat..

[55] Tan TZ (2014). Epithelial-mesenchymal transition spectrum quantification and its efficacy in deciphering survival and drug responses of cancer patients. EMBO Mol. Med..

[56] Smith JJ (2010). Experimentally derived metastasis gene expression profile predicts recurrence and death in patients with colon cancer. Gastroenterology.

[57] Vilar E (2011). MRE11 deficiency increases sensitivity to poly(ADP-ribose) polymerase inhibition in microsatellite unstable colorectal cancers. Cancer Res..

[58] Loboda A (2011). EMT is the dominant program in human colon cancer. BMC Med. Genomics.

[59] Laibe S (2012). A seven-gene signature aggregates a subgroup of stage II colon cancers with stage III. Omics.

[60] Tripathi MK (2014). Nuclear factor of activated T-cell activity is associated with metastatic capacity in colon cancer. Cancer Res..

[61] Marisa L (2013). Gene expression classification of colon cancer into molecular subtypes: characterization, validation, and prognostic value. PLoS Med..

[62] Roepman P (2014). Colorectal cancer intrinsic subtypes predict chemotherapy benefit, deficient mismatch repair and epithelial-to-mesenchymal transition. Int. J. Cancer.

[63] Sole X (2014). Discovery and validation of new potential biomarkers for early detection of colon cancer. PLoS ONE.

[64] Kaiser S (2007). Transcriptional recapitulation and subversion of embryonic colon development by mouse colon tumor models and human colon cancer. Genome Biol..

[65] Sheffer M (2009). Association of survival and disease progression with chromosomal instability: a genomic exploration of colorectal cancer. Proc. Natl Acad. Sci. USA.

[66] Xin J (2016). High-performance web services for querying gene and variant annotation. Genome Biol..

[67] Ritchie ME (2015). limma powers differential expression analyses for RNA-sequencing and microarray studies. Nucleic Acids Res..

[68] Mullner D (2013). fastcluster: fast hierarchical, agglomerative clustering routines for R and Python. J. Stat. Softw..

[69] Bastian M, Heyman M, Jacomy M (2009). Gephi: an open source software for exploring and manipulating networks. Int. AAAI Conf. Weblogs Social Media.

[70] Jacomy, M., Venturini, T., Heymann, S. & Bastian, M. ForceAtlas2, a Continuous Graph Layout Algorithm for Handy Network Visualization Designed for the Gephi Software. *PLoS ONE* **9**, ARTN e98679 10.1371/journal.pone.0098679 (2014). 10.1371/journal.pone.0098679 PMC4051631 24914678

[71] Viechtbauer W (2010). Conducting Meta-Analyses in R with the metafor Package. J. Stat. Softw..

