# Supplementary material for: Parsimonious Gene Correlation Network Analysis (PGCNA): a tool to define modular gene co-expression for refined molecular stratification in cancer
Source: NPJ Syst Biol Appl. 2019 Apr 11;5:13. doi: 10.1038/s41540-019-0090-7 (PMC6459838; doi:10.1038/s41540-019-0090-7)
Supplement: Supplementary file 2 — Supplemental Materials [file 41540_2019_90_MOESM2_ESM.pdf]

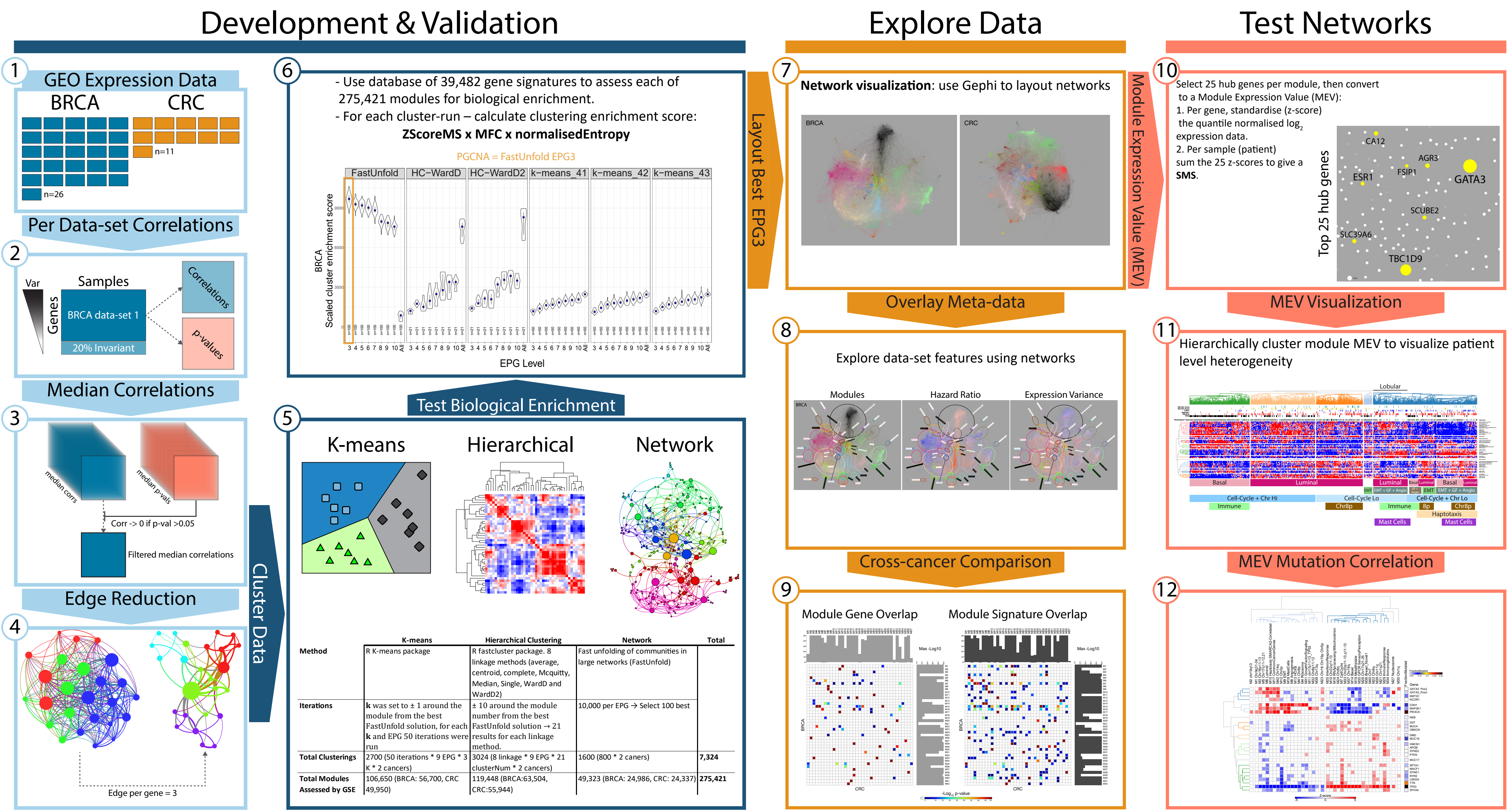

	K-means	Hierarchical Clustering	Network	Total
**Method**	R K-means package	R fastcluster package. 8 linkage methods (average, centroid, complete, Mcquitty, Median, Single, WardD and WardD2)	Fast unfolding of communities in large networks (FastUnfold)	
**Iterations**	**k** was set to ± 1 around the module from the best FastUnfold solution, for each **k** and EPG 50 iterations were run	± 10 around the module number from the best FastUnfold solution → 21 results for each linkage method.	10,000 per EPG → Select 100 best	
**Total Clusterings**	2700 (50 iterations * 9 EPG * 3 K * 2 cancers)	3024 (8 linkage * 9 EPG * 21 clusterNum * 2 cancers)	1600 (800 * 2 caners)	**7,324**
**Total Modules Assessed by GSE**	106,650 (BRCA: 56,700, CRC 49,950)	119,448 (BRCA:63,504, CRC:55,944)	49,323 (BRCA: 24,986, CRC: 24,337)	**275,421**

Supp. Fig.1. Process diagram. Accompanies Figure 1.
Process diagram with numbering of the process linked to Supplemental Methods sections. The diagram is divided into the broad categories of Development & Validation, Data Exploration and Network Testing.

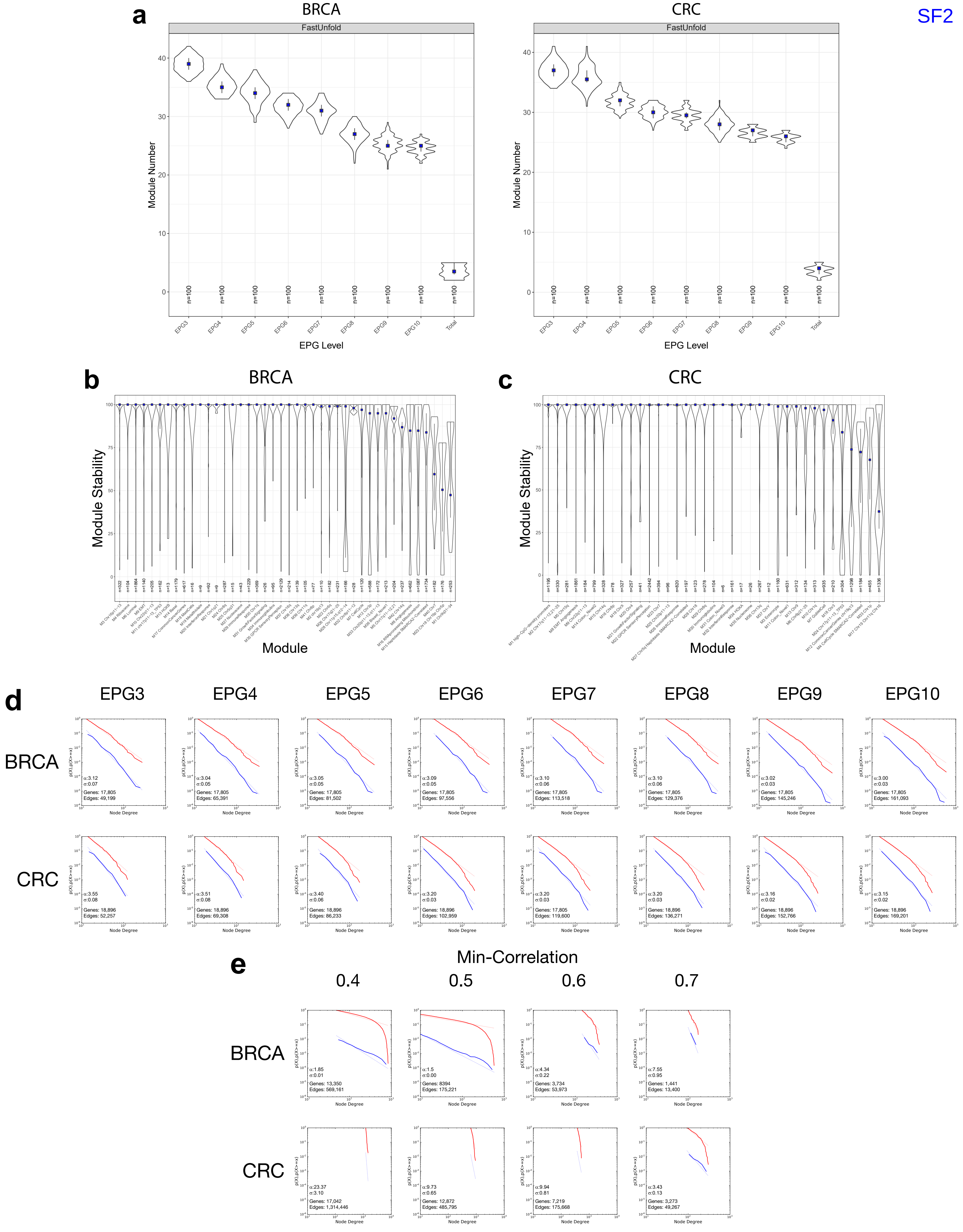

Supp. Fig.2. Edge per gene (EPG) FastUnfold characteristics. Accompanies Figure 1.
(**a**) Violin plots of the distribution module numbers from FastUnfold clustering of matrices with different levels of edge reduction (edges per gene EPG). Upper panel BRCA, lower panel CRC. (**b**) BRCA and (**c**) CRC, violin plots of the stability of module membership generated in the 100 networks evaluated for EPG3 using fast unfolding networks. The module membership of the optimal clustering was set as reference. The degree of overlap of module gene membership with these reference modules is shown as percentage stability across the other 99 network clusterings. Violin plots display the distribution along with median (blue square) and the IQR, ordered by module median stability. (**d/e**) Probability density function (PDF; p(X), blue) and complementary cumulative distribution function (CCDF; p(X<=x), red) of node (gene) degree for (**d**) EPG reduced data (from EPG3—EPG10) (**e**) Hard threshold reduced data with minimum correlation ≥ that indicated (0.4—0.7). Calculated using the python power-law package. Each plot shows the fitted (solid lines) vs PDF/CCDF distributions (dotted lines) along with the fitted α, σ (standard error) and the number of genes/edges for EPG/MinCorrelation.

a

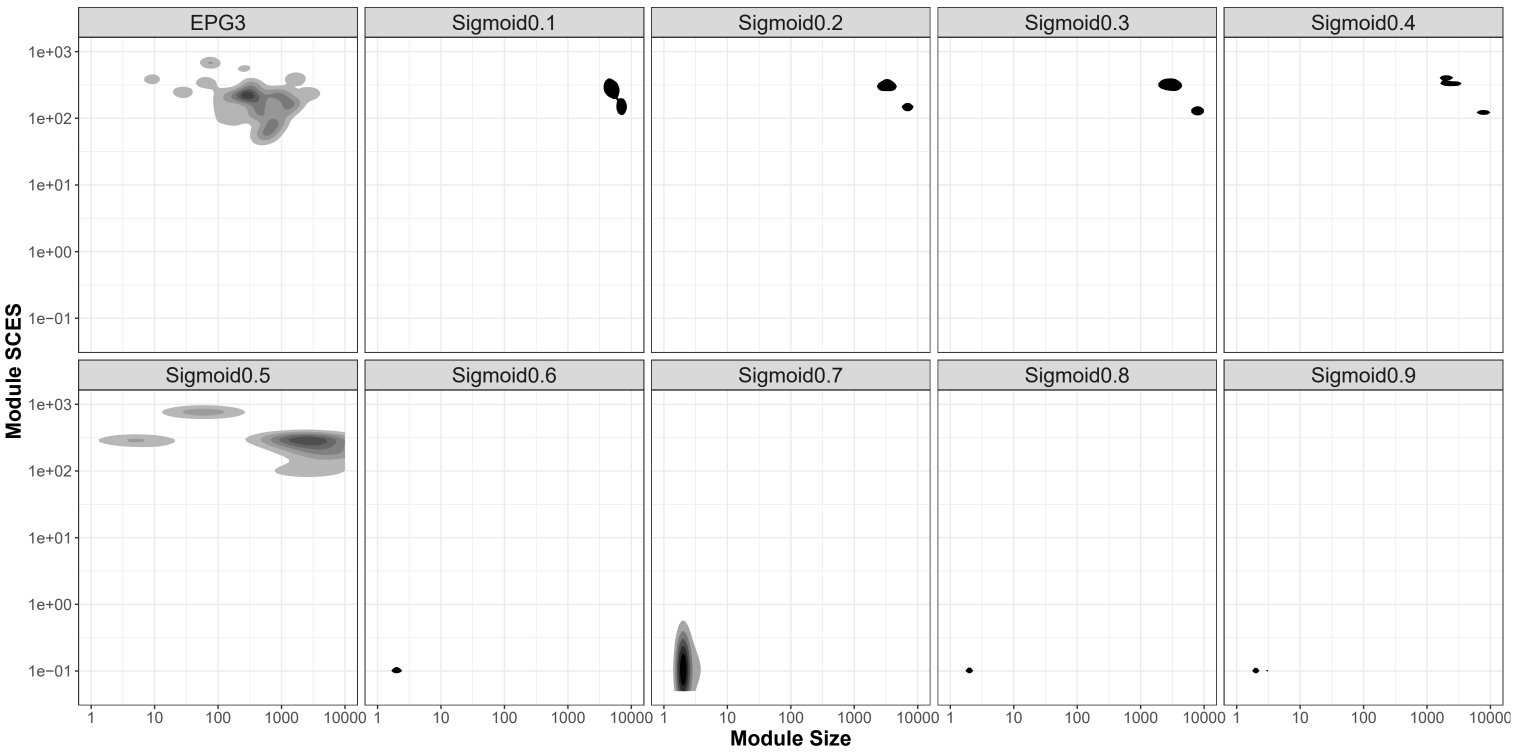

b

Edge Method	Median ModuleNum	Median FiltModNum	Edge Number	Median Sum SCES	Median Module SCES	% ConnectedGenes
EPG3	31	30	46,776	6754	177	100
iPCC	2	2	70,626,665	570	280	100
PowerST2	3	3	141,229,221	832	263	100
PowerST3	3	3	141,229,221	834	254	100
PowerST4	3	3	141,229,072	826	254	100
PowerST5	4	4	141,205,065	1145	272	100
PowerST6	4	4	140,808,064	1144	268	100
PowerST7	4	4	138,582,824	1140	270	100
PowerST8	4	4	131,281,947	1140	273	100
PowerST9	4	4	109,909,640	1142	272	100
PowerST10	4	4	68,513,631	1147	267	100
Sigmoid0.1	3	3	141,229,221	705	261	100
Sigmoid0.2	4	4	141,229,221	1069	298	100
Sigmoid0.3	4	4	141,229,221	1085	314	100
Sigmoid0.4	5	5	77,340,036	1527	322	100
Sigmoid0.5	7	6	20,928,302	1948	275	100
Sigmoid0.6	58	9	6,246,726	1376	150	84
Sigmoid0.7	94.5	20	1,819,581	2818	125	66
Sigmoid0.8	207	26	548,889	3832	140	50
Sigmoid0.9	280.5	38	189,401	5044	113	32

Supp. Fig.3 BRCA edge reduction. Accompanies Figure 1.
Analysis of different edge reduction approaches clustered using the FastUnfold method for the single data-set GSE20685. Each edge reduction method was clustered 10,000 times by FastUnfold and the 100 best clusterings (judged by the modularity score) analysed for gene signature enrichment. (**a**) shows density plots of the module size vs module SCES (Scaled Cluster Enrichment Score; see supplemental methods) across the 100 best clusterings for EPG3 (Edge Per Gene 3) or the WGCNA sigmoid adjacency function varying the shift (μ, 0.1—0.9). (**b**) Table showing results for the different edge reduction strategies for modules with > 5 genes: EPG3 (Edge Per Gene 3), iPCC (iterative Pearson Correlation Coefficient), PowerST (WGCNA Soft Theshold, via adjacency function with power 2—10), Sigmoid (WGCNA sigmoid adjacency function varying the shift (μ, 0.1—0.9)). EdgeMethod: edge reduction strategy, MedianModuleNum: median number of modules per clustering, MedianFiltModNum: median number of modules per clustering with > 5 genes, EdgeNumber: retained edges, MedianSumSCES: median total SCES per clustering, MedianModuleSCES: median SCES per module of clustering, %ConnectedGenes: percentage of genes with at least one edge > 0.01

a

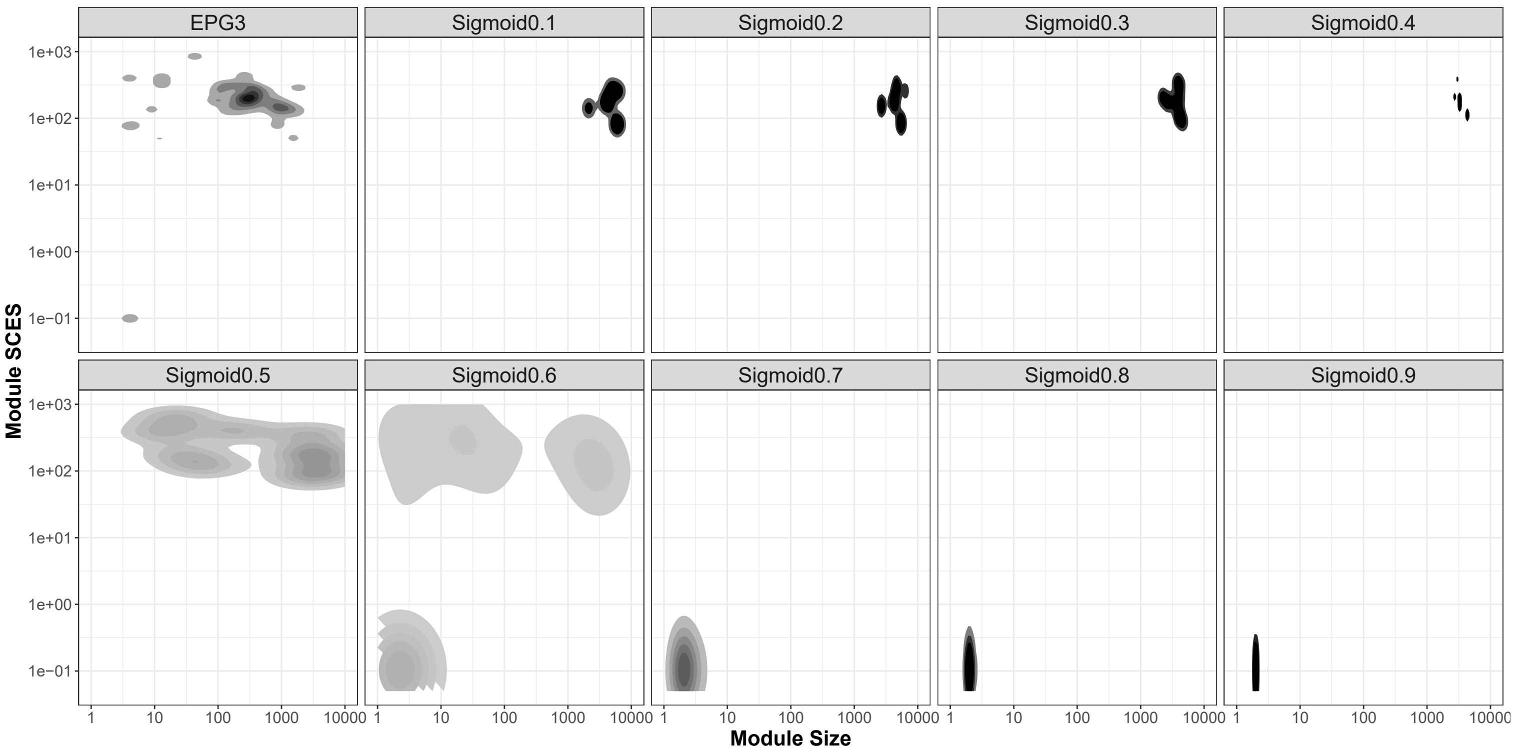

b

Edge Method	Median ModuleNum	Median FiltModNum	Edge Number	Median Sum SCES	Median Module SCES	% ConnectedGenes
EPG3	39	35	45,726	8440	188	100
iPCC	2	2	72,317,350	545	271	100
PowerST2	3	3	141,229,221	819	286	100
PowerST3	3	3	141,229,219	819	286	100
PowerST4	3	3	141,229,167	819	284	100
PowerST5	3	3	141,198,009	820	282	100
PowerST6	3	3	140,242,600	819	279	100
PowerST7	4	4	134,485,398	1078	259	100
PowerST8	4	4	119,689,653	1078	256	100
PowerST9	4	4	96,215,114	1085	255	100
PowerST10	4	4	69,574,677	1090	255	100
Sigmoid0.1	3.5	4	141,229,221	593	170	100
Sigmoid0.2	4	4	141,229,221	714	168	100
Sigmoid0.3	5	5	141,229,221	1040	173	100
Sigmoid0.4	5	5	101,836,373	1156	198	100
Sigmoid0.5	10	10	46,170,298	2642	186	100
Sigmoid0.6	28	13	16,544,570	2795	152	100
Sigmoid0.7	70	21	4,741,206	2922	139	97
Sigmoid0.8	149	29	1,184,292	3817	94	86
Sigmoid0.9	263	33	298,088	4728	119	61

Supp. Fig.4. CRC edge reduction. Accompanies Figure 1.
Analysis of different edge reduction approaches clustered using the FastUnfold method for the single data-set GSE39582. Each edge reduction method was clustered 10,000 times by FastUnfold and the 100 best clusterings (judged by the modularity score) analysed for gene signature enrichment. (**a**) shows density plots of the module size vs module SCES (Scaled Cluster Enrichment Score; see supplemental methods) across the 100 best clusterings for EPG3 (Edge Per Gene 3) or the WGCNA sigmoid adjacency function varying the shift (μ, 0.1—0.9). (**b**) Table showing results for the different edge reduction strategies for modules with > 5 genes: EPG3 (Edge Per Gene 3), iPCC (iterative Pearson Correlation Coefficient), PowerST (WGCNA Soft Theshold, via adjacency function with power 2—10), Sigmoid (WGCNA sigmoid adjacency function varying the shift (μ, 0.1—0.9)). EdgeMethod: edge reduction strategy, MedianModuleNum: median number of modules per clustering, MedianFiltModNum: median number of modules per clustering with > 5 genes, EdgeNumber: retained edges, MedianSumSCES: median total SCES per clustering, MedianModuleSCES: median SCES per module of clustering, %ConnectedGenes: percentage of genes with at least one edge > 0.01

a

Dataset	Clustering	ZScoreMaxSum	MedianFractionContribution	NormalisedEntropy	ZscoreMS*MFC*NormalisedEntropy(SCES)	Type
BRCA GSE20685	Clust27	9128.30	1.00	0.84	7700.58	PGCNA
	Clust59	8882.30	1.00	0.85	7583.62	PGCNA
	Clust2	8720.21	1.00	0.85	7426.62	PGCNA
	Clust31	8499.63	1.00	0.87	7411.34	PGCNA
	Clust7	8621.51	1.00	0.84	7267.66	PGCNA
	Clust16	9510.86	0.92	0.83	7239.08	PGCNA
	SoftT4	5224.27	0.71	0.62	2276.45	WGCNA
	SoftT3	4703.81	0.73	0.60	2086.32	WGCNA
	SoftT2	4966.44	0.61	0.62	1906.29	WGCNA
	SoftT6	4467.93	0.73	0.57	1857.92	WGCNA
	SoftT5	4506.22	0.70	0.58	1834.98	WGCNA
	SoftT7	5227.30	0.68	0.52	1827.57	WGCNA
CRC GSE39582	Clust97	13262.79	1.00	0.84	11113.76	PGCNA
	Clust4	12338.79	1.00	0.85	10524.08	PGCNA
	Clust3	12862.40	1.00	0.82	10516.06	PGCNA
	Clust38	12040.60	1.00	0.84	10120.11	PGCNA
	Clust100	12192.89	1.00	0.83	10093.07	PGCNA
	Clust20	12067.99	1.00	0.83	10071.56	PGCNA
	SoftT7	8568.38	0.64	0.71	3907.94	WGCNA
	SoftT6	7284.81	0.67	0.75	3658.82	WGCNA
	SoftT5	5794.15	0.62	0.78	2814.72	WGCNA
	SoftT4	4553.88	0.65	0.78	2299.54	WGCNA
	SoftT3	4688.52	0.60	0.79	2226.79	WGCNA
	SoftT2	4571.92	0.62	0.76	2127.88	WGCNA

b

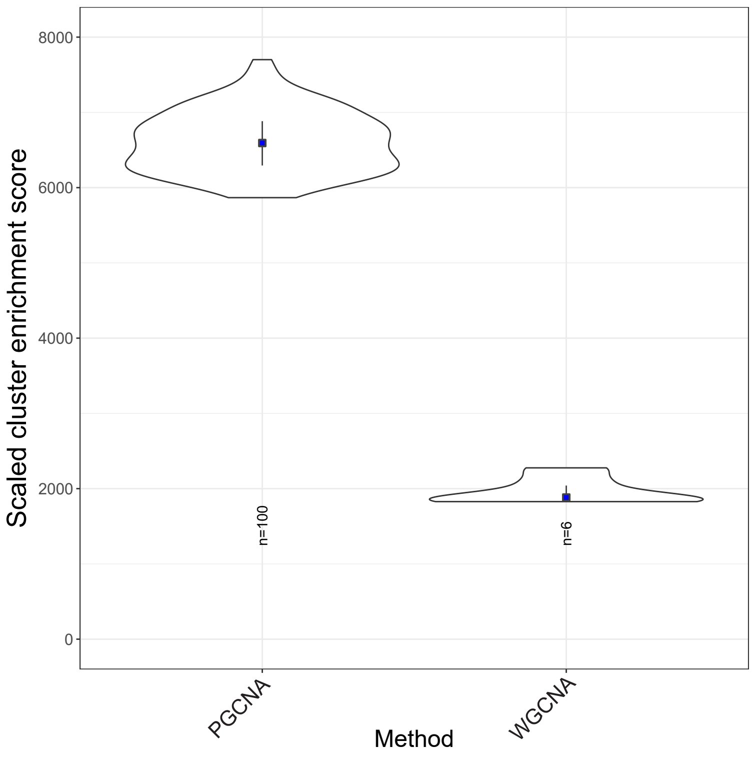

c

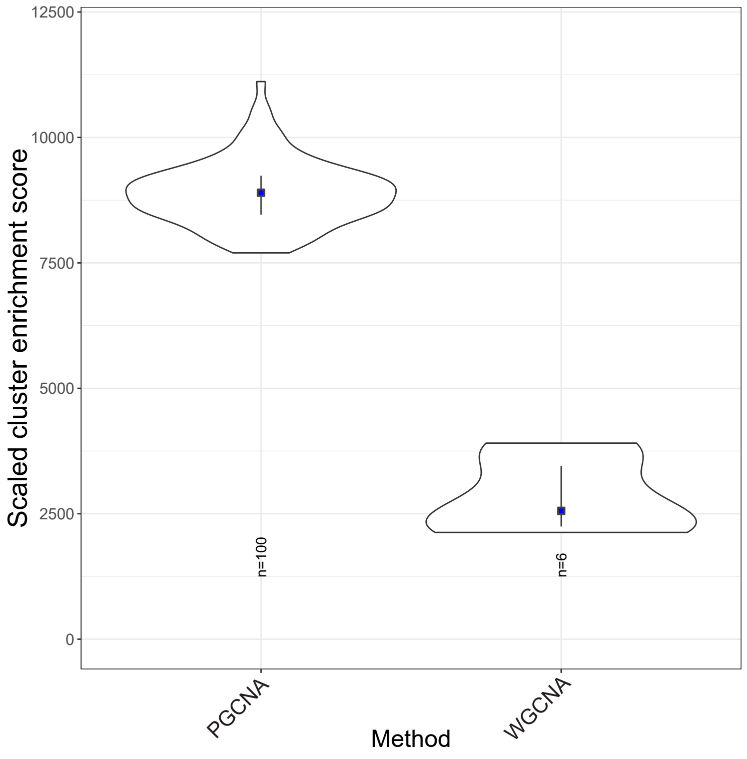

Supp. Fig.5. Gene signature enrichment of PGCNA/WGCNA
(**a**) table of the top/bottom 6 clusterings of the data (ranked by scaled cluster enrichment score; **SCES**) for BRCA (top panel) and CRC (bottom panel). Showing the Clustering (out of 100 FastUnfold tested, or soft-threshold/softT level), ZScoreMaxSum, MedianFractionContribution, NormalisedEntropy and ZScoreMS·MFC·NormalisedEntropy (**SCES**; used in violin plots). (**b/c**) Violin plots showing range of SCES across the 100 retained FastUnfold and 6 WGCNA (soft-threshold 2—7) clusterings. (**b**) BRCA, (**c**) CRC. Violin plots show distribution with median (blue square) and IQR (black line). See **Cluster selection** in supplemental methods for details on scores.

SF6

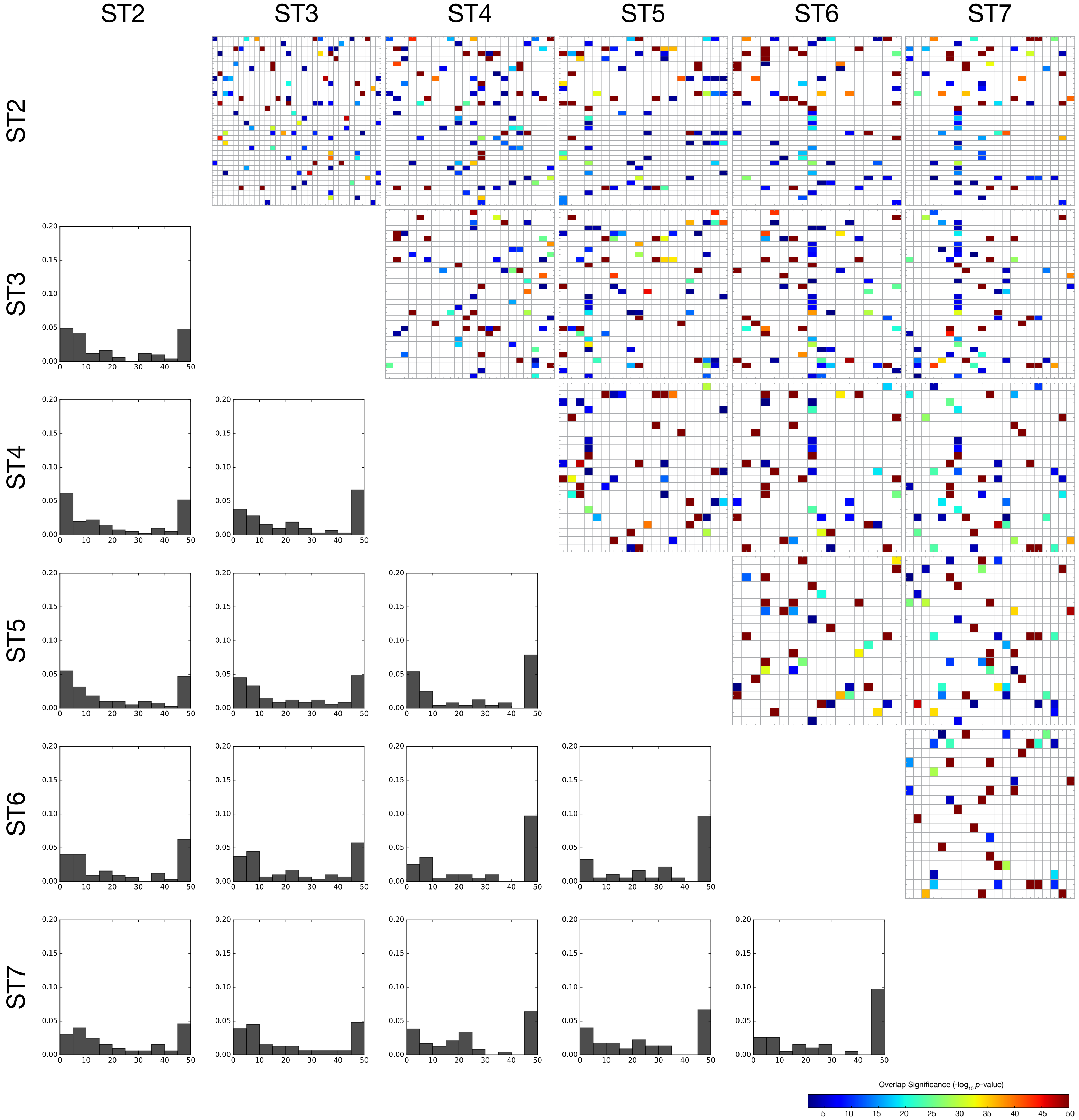

Supp. Fig.6. WGCNA BRCA module similarity across soft-threshold range. **Top-right triangle**: pairwise (soft-thresholds) module gene overlap, showing -$\log_{10}$ *p*-values (hypergeometric test), **bottom-left triangle**: corresponding pairwise histograms showing -$\log_{10}$ *p*-values (x-axis) vs density (y-axis) for the values in the gene overlap plots. -$\log_{10}$ *p*-values were prefiltered to remove all < 2 (*p*-value > 0.01) before images drawn. Bottom right shows -$\log_{10}$ *p*-value colour scale for top-right overlap figures.

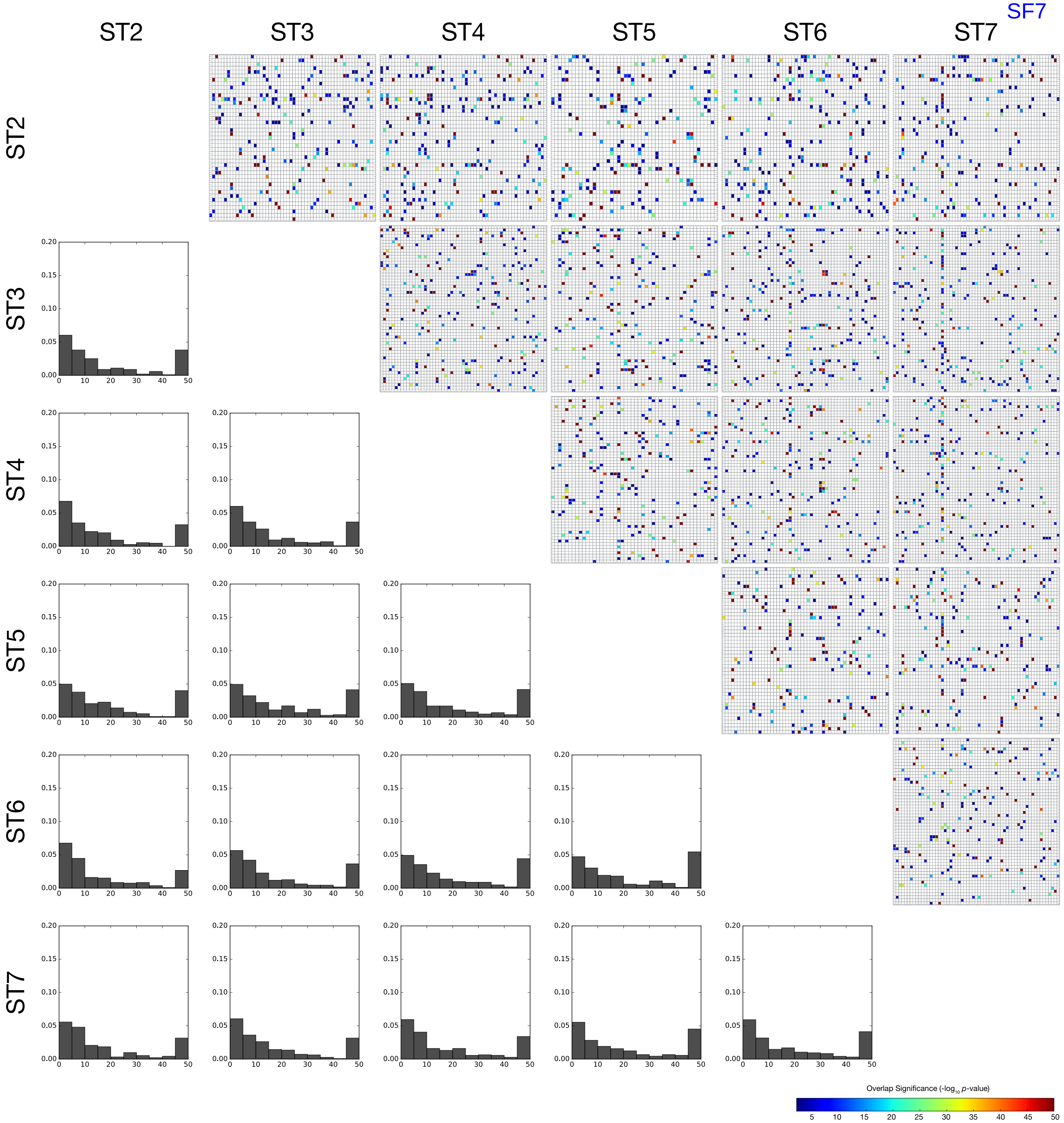

Supp. Fig.7. WGCNA CRC module similarity across soft-threshold range. **Top-right triangle**: pairwise (soft-thresholds) module gene overlap, showing -$\log_{10}$ *p*-values (hypergeometric test), **bottom-left triangle**: corresponding pairwise histograms showing -$\log_{10}$ *p*-values (x-axis) vs density (y-axis) for the values in the gene overlap plots. -$\log_{10}$ *p*-values were prefiltered to remove all < 2 (*p*-value > 0.01) before images drawn. Bottom right shows -$\log_{10}$ *p*-value colour scale for top-right overlap figures.

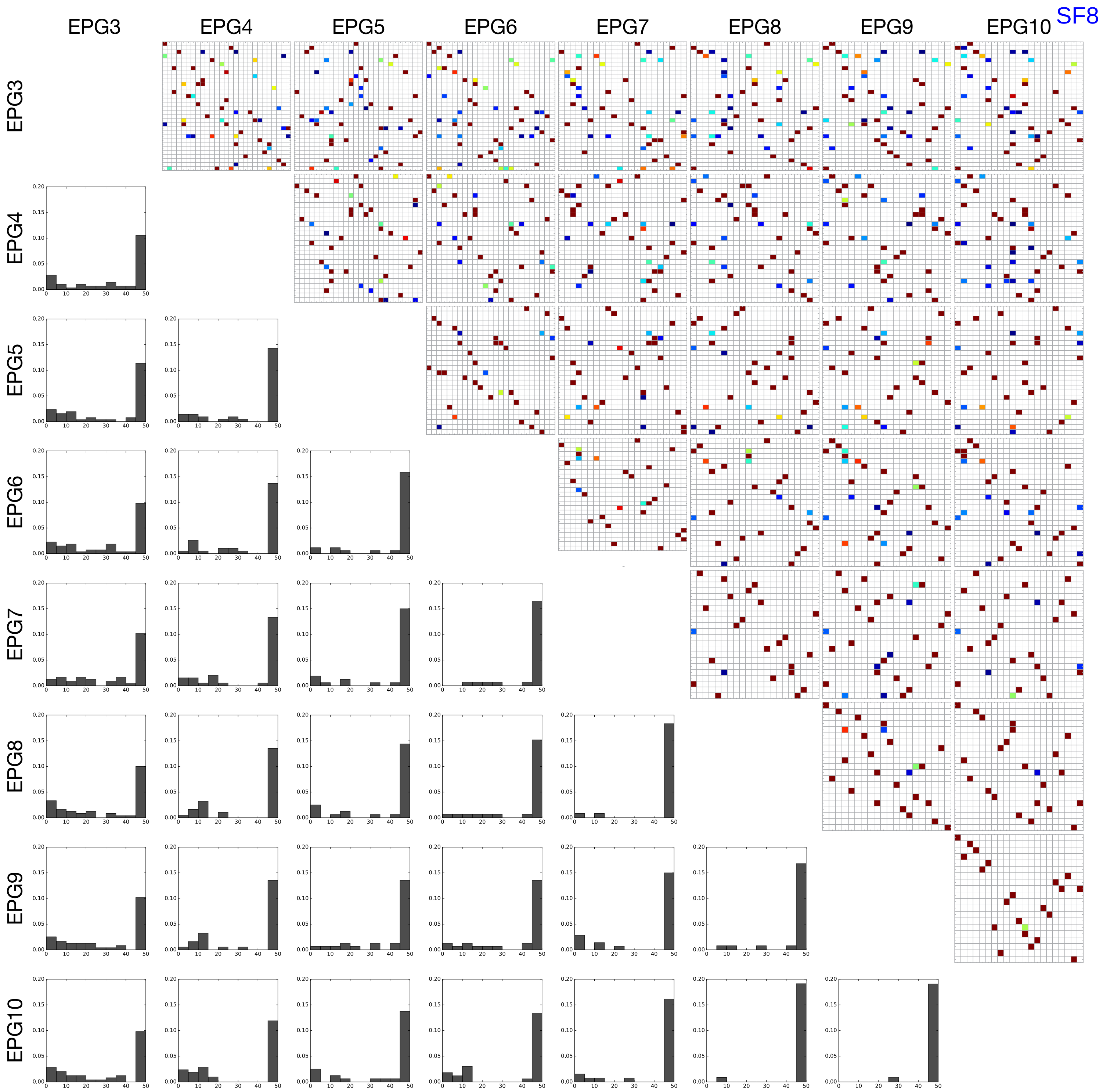

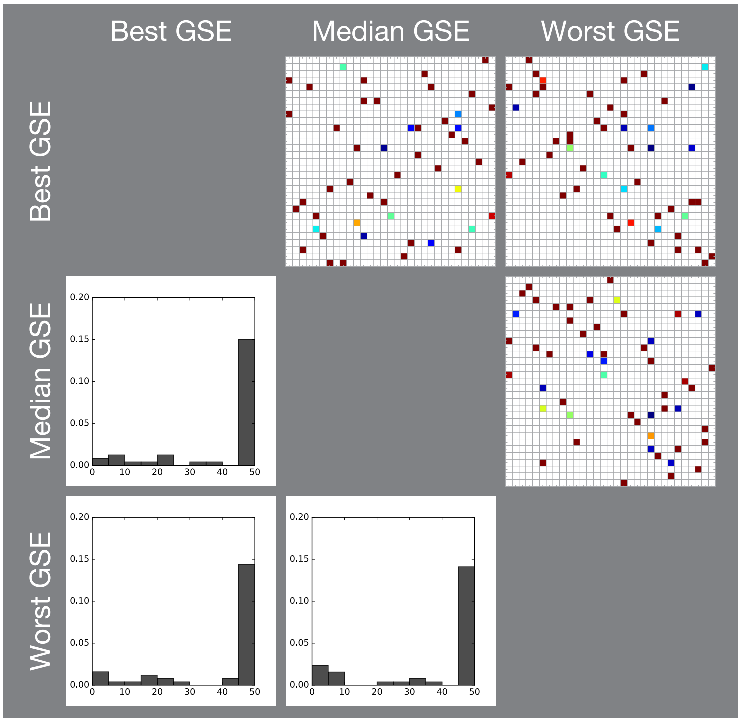

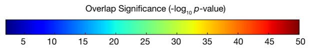

Supp. Fig.8. PGCNA BRCA module similarity across EPG range. Top-right triangle: pairwise (EPG) module gene overlap, showing -log10 p-values (hypergeometric test), **bottom-left triangle**: corresponding pairwise histograms showing $-\log_{10}$ *p*-values (x-axis) vs density (y-axis) for the values in the gene overlap plots. These figures are using the first of the 100 retained FastUnfold clusterings, so are not selected by scaled cluster enrichment score (**SCES**). **Grey box: PGCNA BRCA module similarity across SCES enrichment range for EPG3**. An equivalent set of figures comparing the best, median and worst EPG3 clustering (from 100 retained, see Supp. Methods Fig.4 b/c) as judged by scaled cluster enrichment score (SCES). $-\log_{10}$ *p*-values were prefiltered to remove all < 2 (*p*-value > 0.01) before images drawn. Bottom right shows $-\log_{10}$ *p*-value colour scale for overlap figures.

SF9

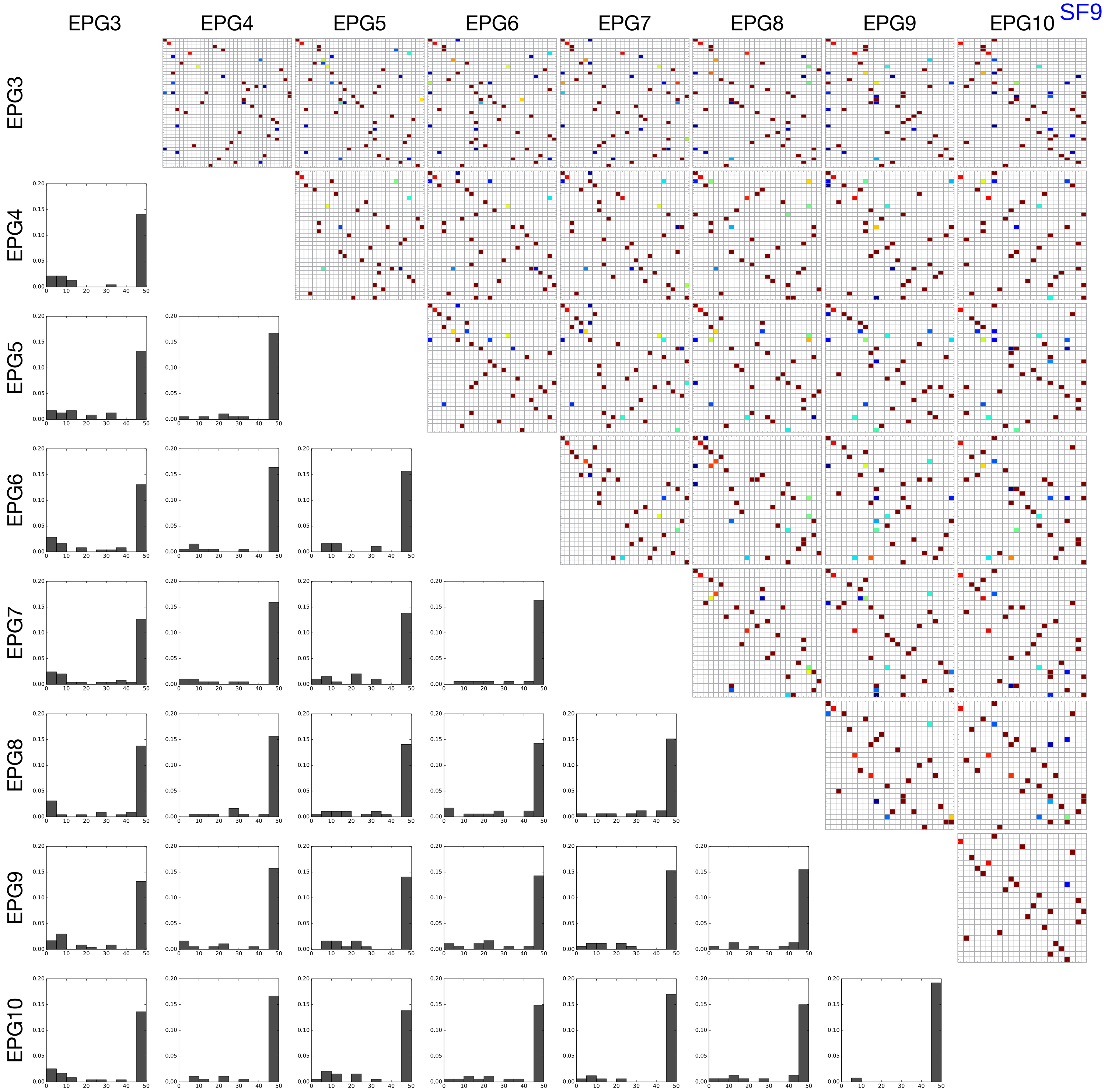

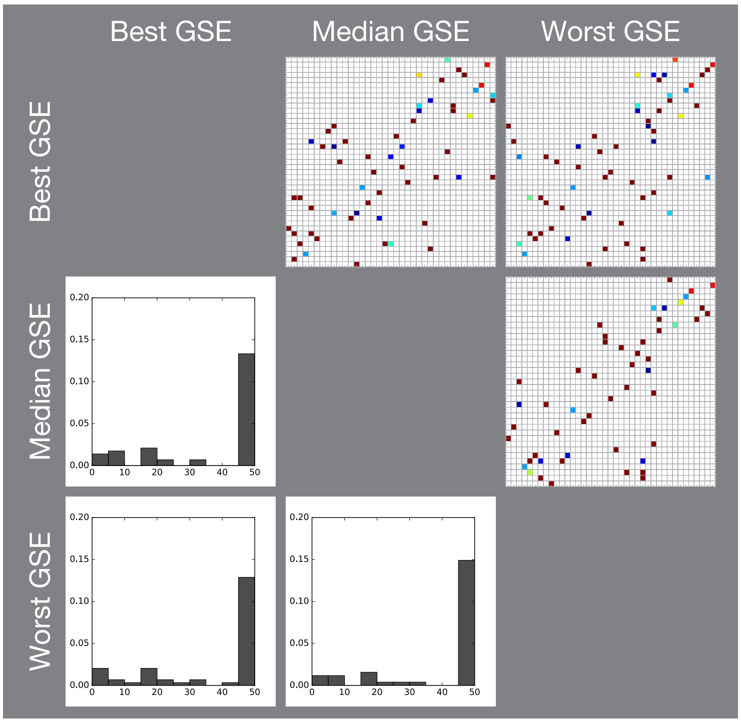

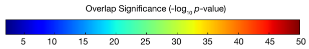

Supp. Fig.9. PGCNA CRC module similarity across EPG range. Top-right triangle: pairwise (EPG) module gene overlap, showing -log10 p-values (hypergeometric test), **bottom-left triangle**: corresponding pairwise histograms showing $-\log_{10}$ *p*-values (x-axis) vs density (y-axis) for the values in the gene overlap plots. These figures are using the first of the 100 retained FastUnfold clusterings, so are not selected by scaled cluster enrichment score (**SCES**). **Grey box: PGCNA CRC module similarity across SCES enrichment range for EPG3**. An equivalent set of figures comparing the best, median and worst EPG3 clustering (from 100 retained, see Supp. Methods Fig.4 b/c) as judged by scaled cluster enrichment score (SCES). $-\log_{10}$ *p*-values were prefiltered to remove all < 2 (*p*-value > 0.01) before images drawn. Bottom right shows $-\log_{10}$ *p*-value colour scale for overlap figures.

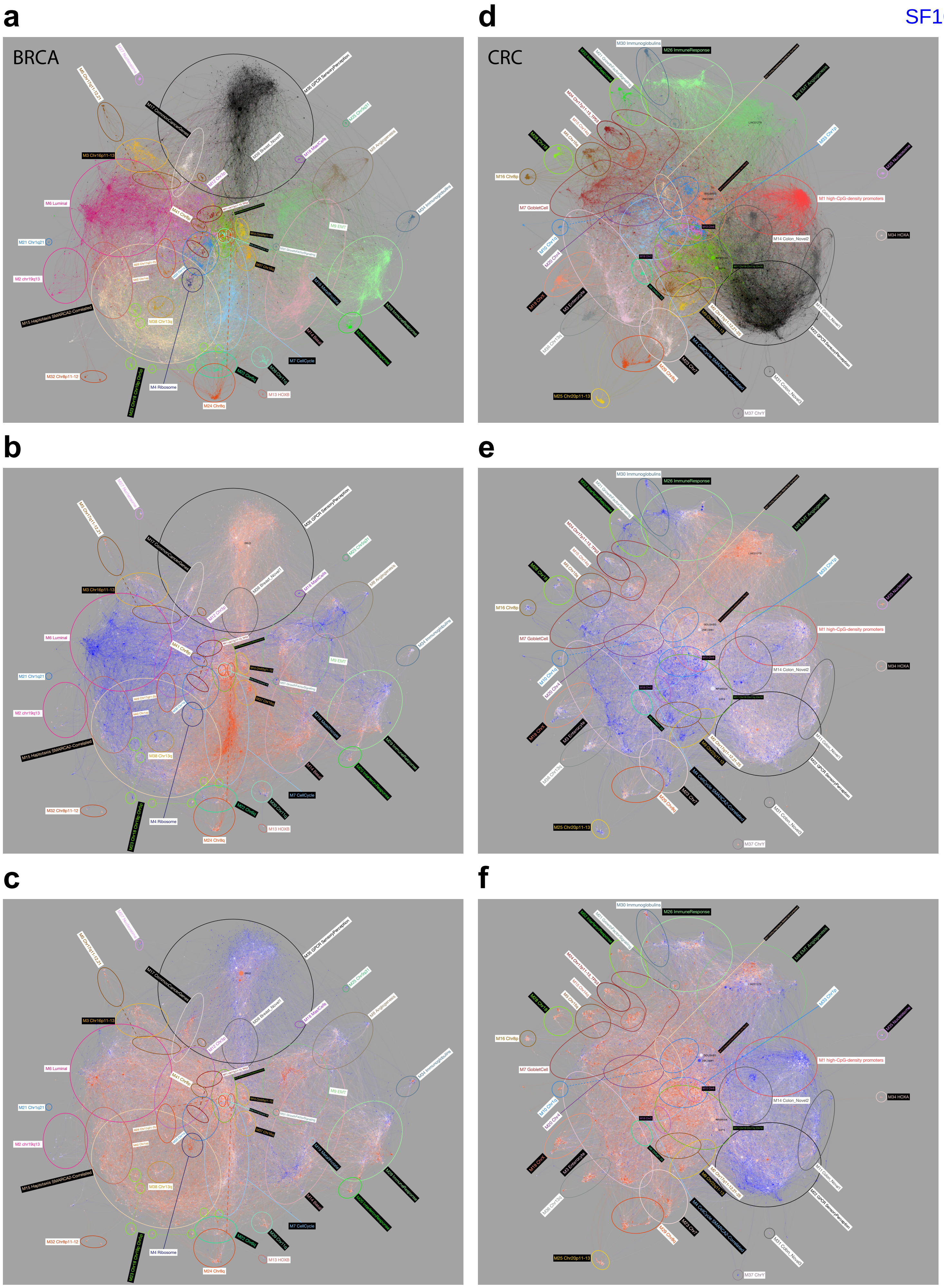

Supp. Fig.10. Network visualization. Accompanies, Figure 2 and Figure 5.
Detailed annotation of the network generated by PGCNA for BRCA displaying the optimal clustering as shown in Fig. 2, with all modules annotated and labelled with their respective summary designation. (**a**) displays primary module color coding for BRCA, with shared coding where applicable to the closest matching module for CRC in (d), (**b**) shows module annotations in the context of the network meta hazard ratio overlay, and (**c**) annotation in the context of expression percentile. (**d**) Annotated primary module color coded version of CRC network, (**e**) annotation in relation to meta hazard ratio, and (**f**) annotation in the context of expression percentile. Color scales: expression percentile blue (least) to red (most); outcome blue (low HR - good outcome) to red (high HR - poor outcome). Networks and linked resources are available as fully searchable interactive tools at http://pgcna.gets-it.net/

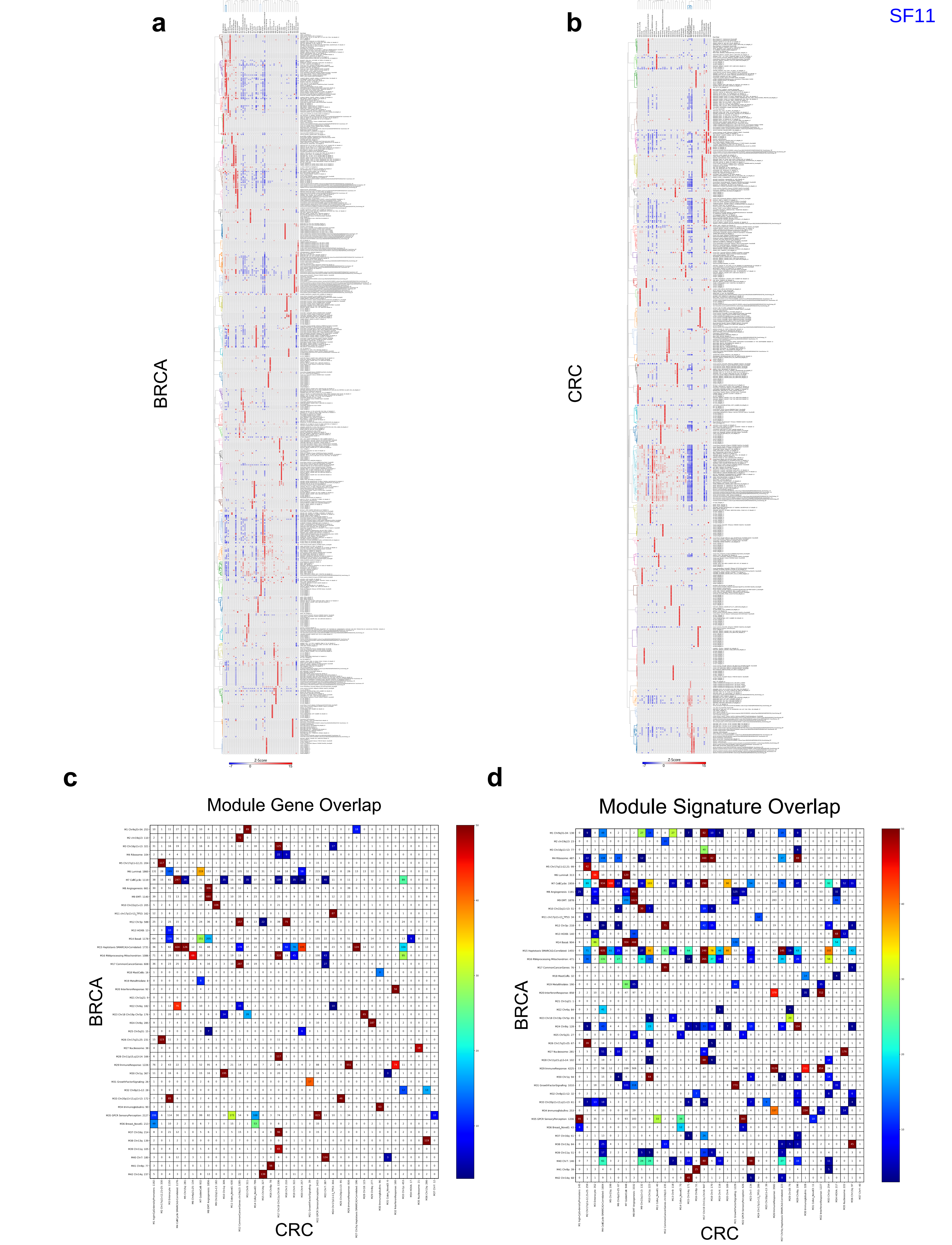

Supp. Fig.11. Gene signature and ontology enrichments and overlap of module gene membership between BRCA and CRC. Accompanies Figure 3.

High-resolution version of heatmaps of gene signature and ontology term enrichments for the network modules of (**a**) BRCA and (**b**) CRC. Module numbers and designations are listed on the x-axis, and signature/ontology terms on the y-axis, modules were clustered according to gene signature enrichment using hierarchical clustering (using top 15 signatures per module; FDR <0.05). Gene signature enrichments are illustrated as a red/blue color code reflecting significance (z-score) of enrichment, complete lists of all signature enrichment results including the contributing genes are provided in Supp.Table 3&4 and online resources. (**c**) Heatmap of module gene membership overlap and (**d**) module gene signature enrichment overlap in the pairwise comparison of BRCA (y-axis) and CRC (x-axis) modules. In each instance the number of genes included in the module is show along with the module number and summary term. Within the pairwise comparison the significance of overlap is illustrated in the indicated color scale of -$\log_{10}$ p-value. For each pairwise comparison the number of overlapping genes is indicated in the relevant square.

SF12

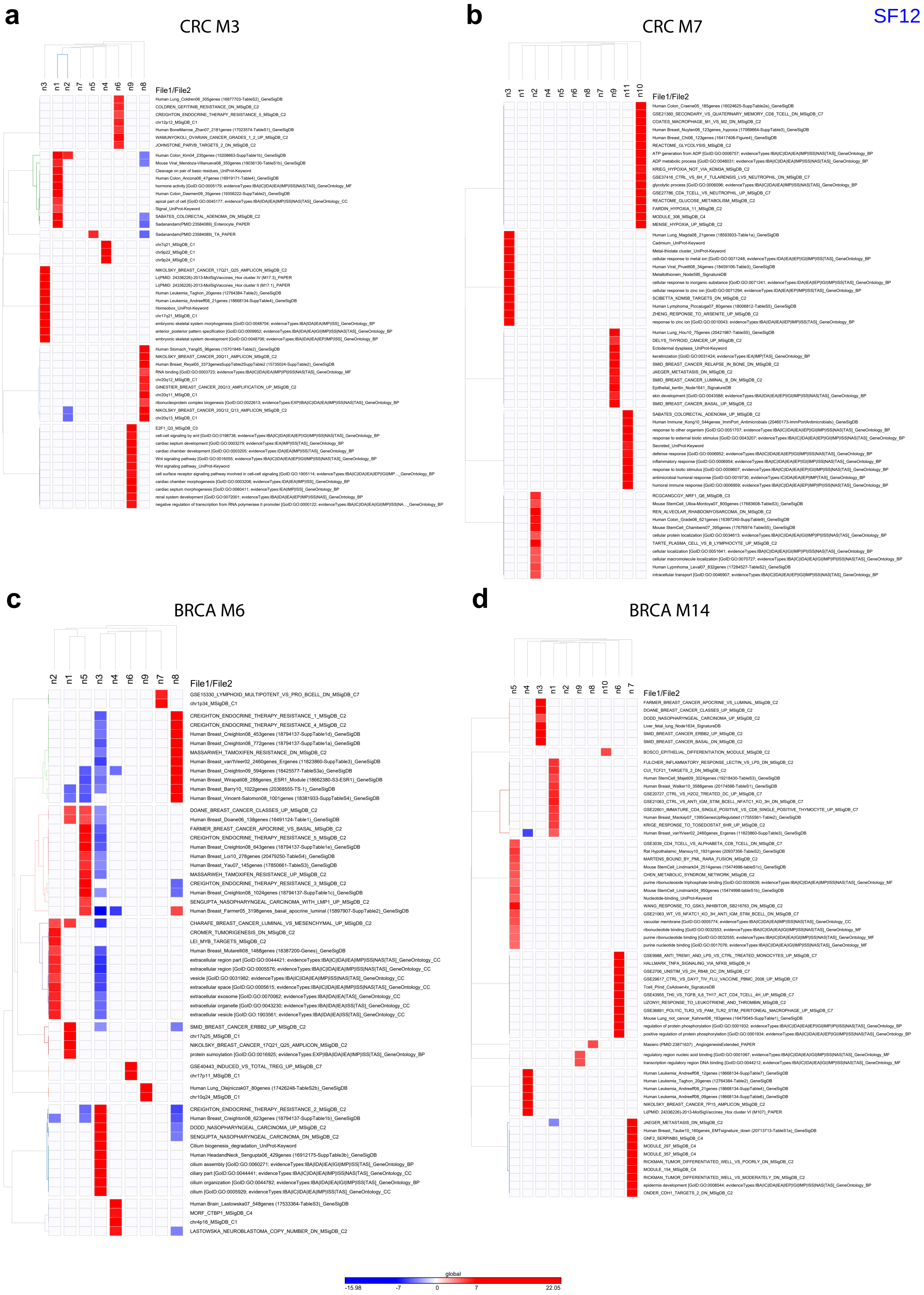

Supp. Fig.12. Gene signature and ontology enrichments in epithelial differentiation module neighborhoods. Accompanies Figure 4.
These heatmaps illustrate the results of gene signature and ontology term enrichment analysis for the module neighborhood analysis shown in Figure 4, (**a**) CRC_M3 enterocyte, (**b**) CRC_M7 goblet cell, (**c**) BRCA_M6 luminal, (**d**) BRCA_M14 basal. Neighborhood numbers and designations are listed on the x-axis, and signature/ontology terms on the y-axis, modules were clustered according to gene signature enrichment using hierarchical clustering (using top 15 signatures per module; FDR <0.05). Gene signature enrichments are illustrated as a red/blue color code reflecting significance (z-score) of enrichment, complete lists of all signature enrichment results including the contributing genes are provided in Supp. Table 6 and online resources.

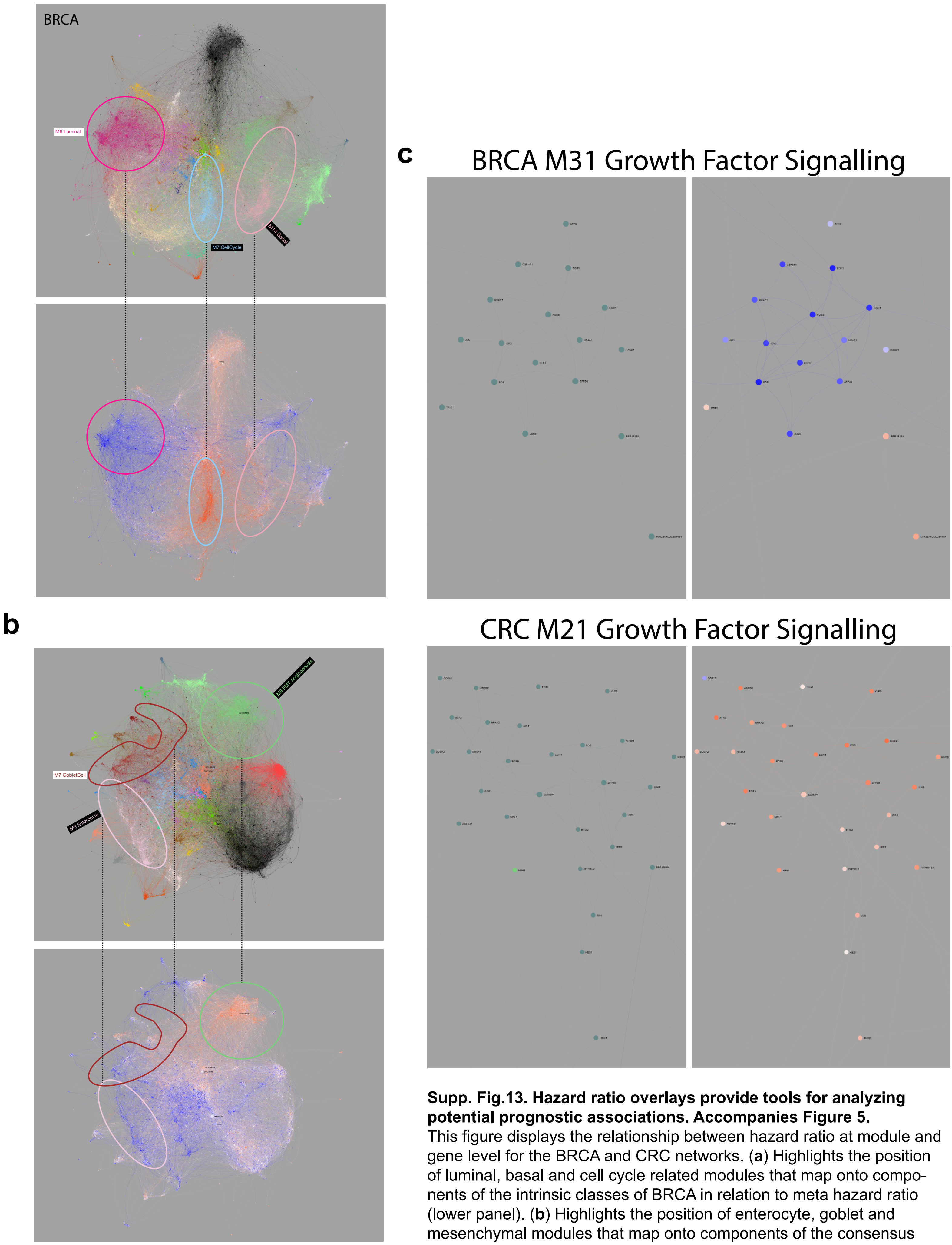

Supp. Fig.13. Hazard ratio overlays provide tools for analyzing potential prognostic associations. Accompanies Figure 5.
This figure displays the relationship between hazard ratio at module and gene level for the BRCA and CRC networks. (**a**) Highlights the position of luminal, basal and cell cycle related modules that map onto components of the intrinsic classes of BRCA in relation to meta hazard ratio (lower panel). (**b**) Highlights the position of enterocyte, goblet and mesenchymal modules that map onto components of the consensus molecular subtype classes of CRC in relation to hazard ratio (lower panel). (**c**) Illustrates the core components of the growth factor signaling modules of BRCA and CRC with the juxtaposition of the hazard ratio

SF14

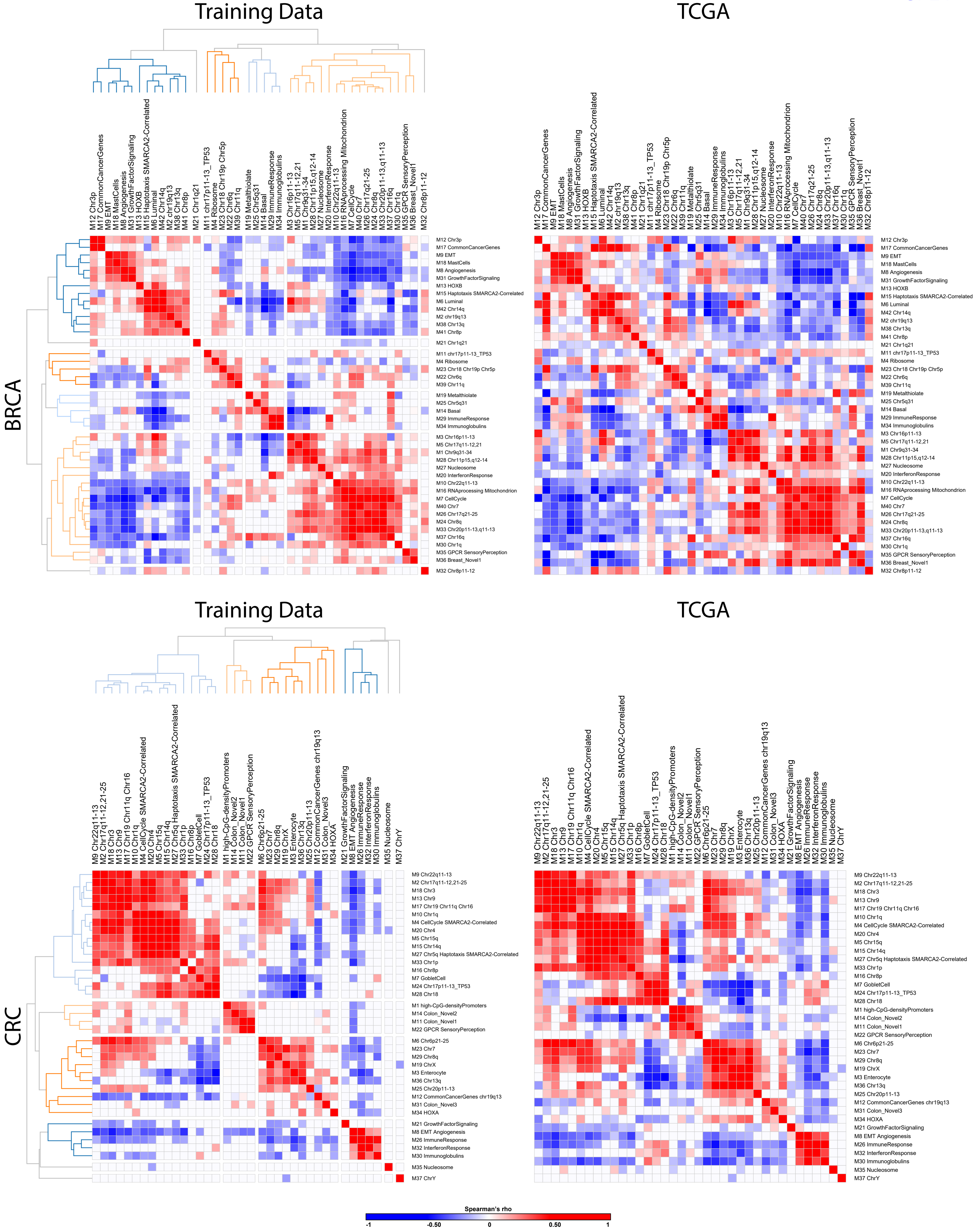

Supp. Fig.14. Assessment of network module co-occurrence across all samples in training array data sets and TCGA RNA-seq data. Accompanies Figure 6. Correlation heatmaps of the co-occurrence of network modules in array training data (left panels) and TCGA RNAseq data (right panels) for BRCA (upper panels) and CRC (lower panels). Module expression values (MEV) were generated for all samples from the 25 (or less for smaller modules) hub genes of each module (see Online methods). The relationship of the modules was analyzed by calculating the Spearman's rank correlation for all module (as MEV) pairs within each data set. These were then merged across data sets by calculating the median correlation and p-values. A final matrix generated by setting all correlations with a p-value > 0.05 to 0. Within GENE-E the 'training data' was hierarchically clustered (Pearson correlations and average linkage) and the TCGA data displayed in the same order without hierarchical clustering.

SF15

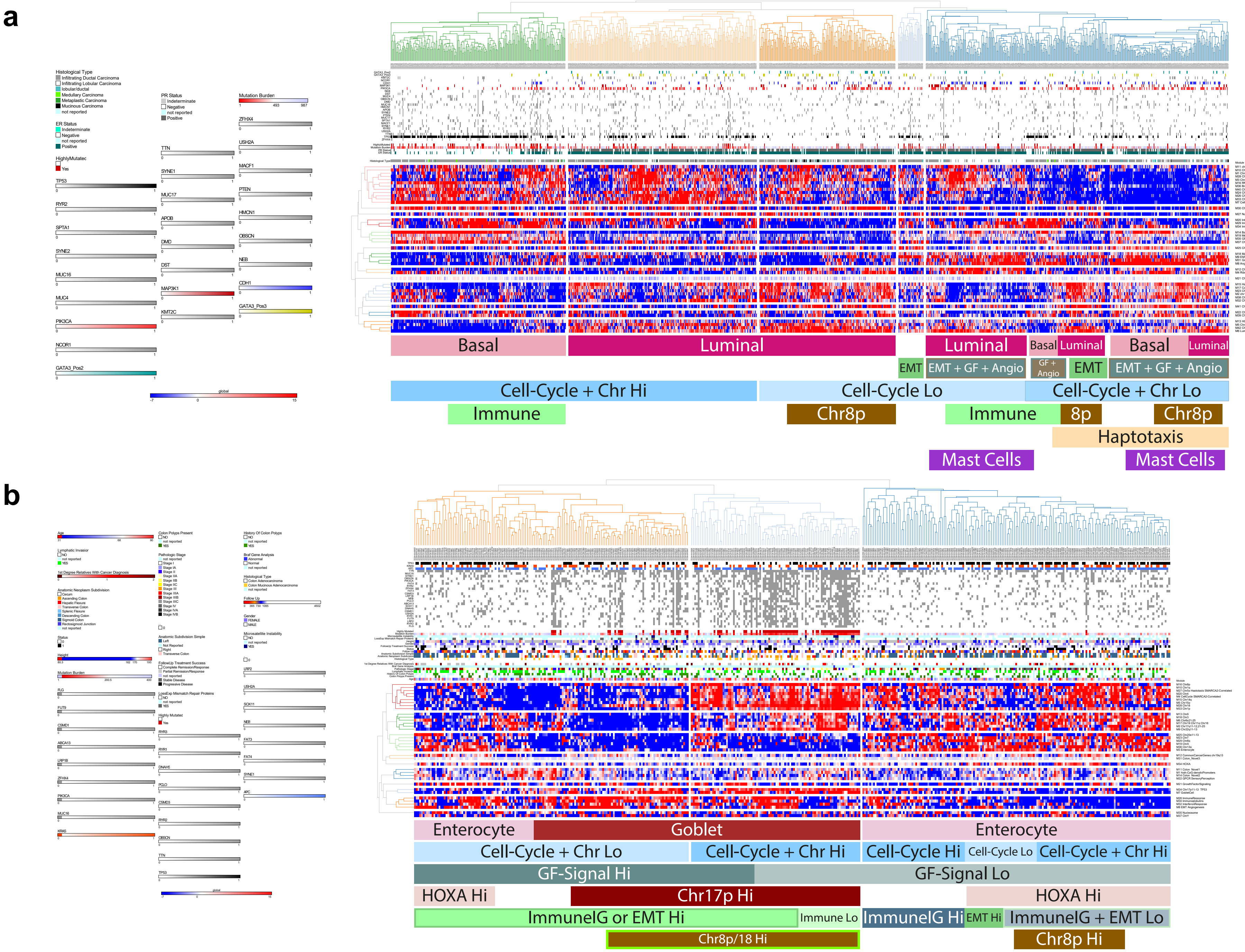

Supp. Fig.15. Module expression values as a platform for a bar-code of gene expression

Use of module expression values in hierarchical clustering of TGCA data (**a**) BRCA. Illustrated are the distribution of mutations in common target genes above the expression values as colored bars GATA3_Pos2 (green), GATA3_Pos3 (yellow), CDH1 (blue), MAP3K1 (dark red), PIK3CA (red), TP53 (black) as well as a wider range of less frequently mutated target genes (grey). Beneath this is illustrated assessment of highly mutated status and mutation burden, ER and PR status, and histological type. Beneath the heatmap indicative examples of the modules linked to heterogeneity in the principle branches of the tree are identified, as indicated in the figure key to the left of the heatmap. (**b**) CRC. Illustrated above the heatmap are the distribution of mutations in common target genes as colored bars, TP53 (black), KRAS (red) and APC (blue), beneath these mutation events in a wider set of representative genes (grey). The distribution of dichotomized highly mutated cases is shown in red, beneath this refined assessment of mutation burden is provided (blue to red color scale). A range of other meta-data is indicated in the figure key to the left of the heat-map and includes histological type, anatomical subdivision, pathological stage, colon polyps, BRAF gene analysis, micro-satellite instability. Beneath the heatmap indicative examples of the modules linked to heterogeneity in the principle branches of the tree are identified.

SF16

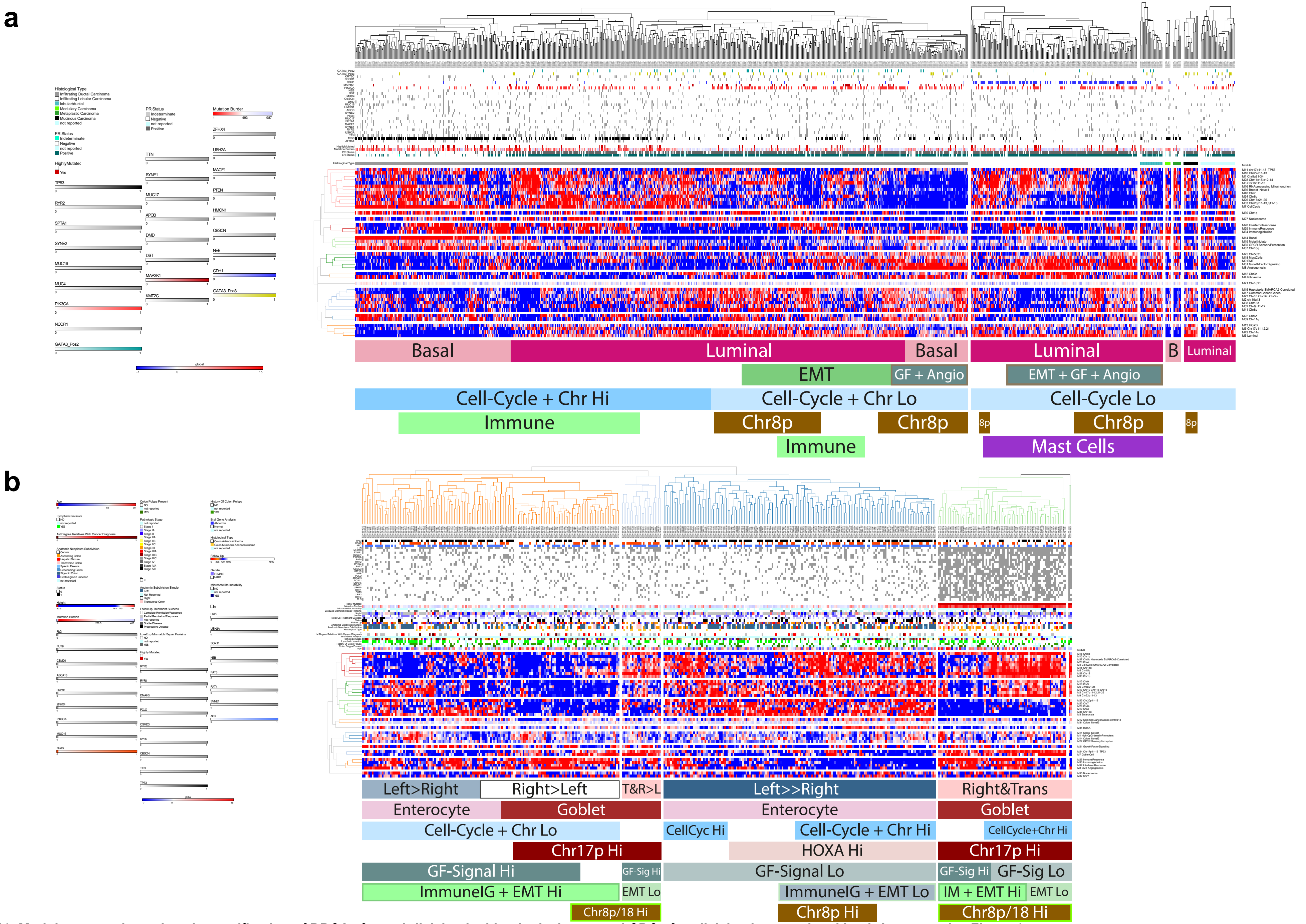

Supp. Fig.16. Module expression values in stratification of BRCA after subdivision by histological type, and CRC after division by mutational load. Accompanies Figure 6.
(**a**) Illustrates the results of applying hierarchical clustering using MEVSs for BRCA cases after separating by histological type. Illustrated are the distribution of mutations in common target genes above the expression values as colored bars GATA3_Pos2 (green), GATA3_Pos3 (yellow), CDH1 (blue), MAP3K1 (dark red), PIK3CA (red), TP53 (black) as well as a wider range of less frequently mutated target genes (grey). Beneath this is illustrated assessment of highly mutated status and mutation burden, ER and PR status, and histological type. Beneath the heatmap indicative examples of the modules linked to heterogeneity in the principle branches of the tree are identified, as indicated in the figure key to the left of the heatmap. (**b**) Illustrates the results of hierarchical clustering of CRC cases using MEVs after subdivision by hypermutation status. Illustrated above the heatmap are the distribution of mutations in common target genes as colored bars, TP53 (black), KRAS (red) and APC (blue), beneath these mutation events in a wider set of representative genes (grey). The distribution of dichotomized highly mutated cases is shown in red, beneath this refined assessment of mutation burden is provided (blue to red color scale). A range of other meta-data is indicated in the figure key to the left of the heat-map and includes histological type, anatomical subdivision, pathological stage, colon polyps, BRAF gene analysis, micro-satellite instability. Beneath the heatmap indicative examples of the modules linked to heterogeneity in the principle branches of the tree are identified.

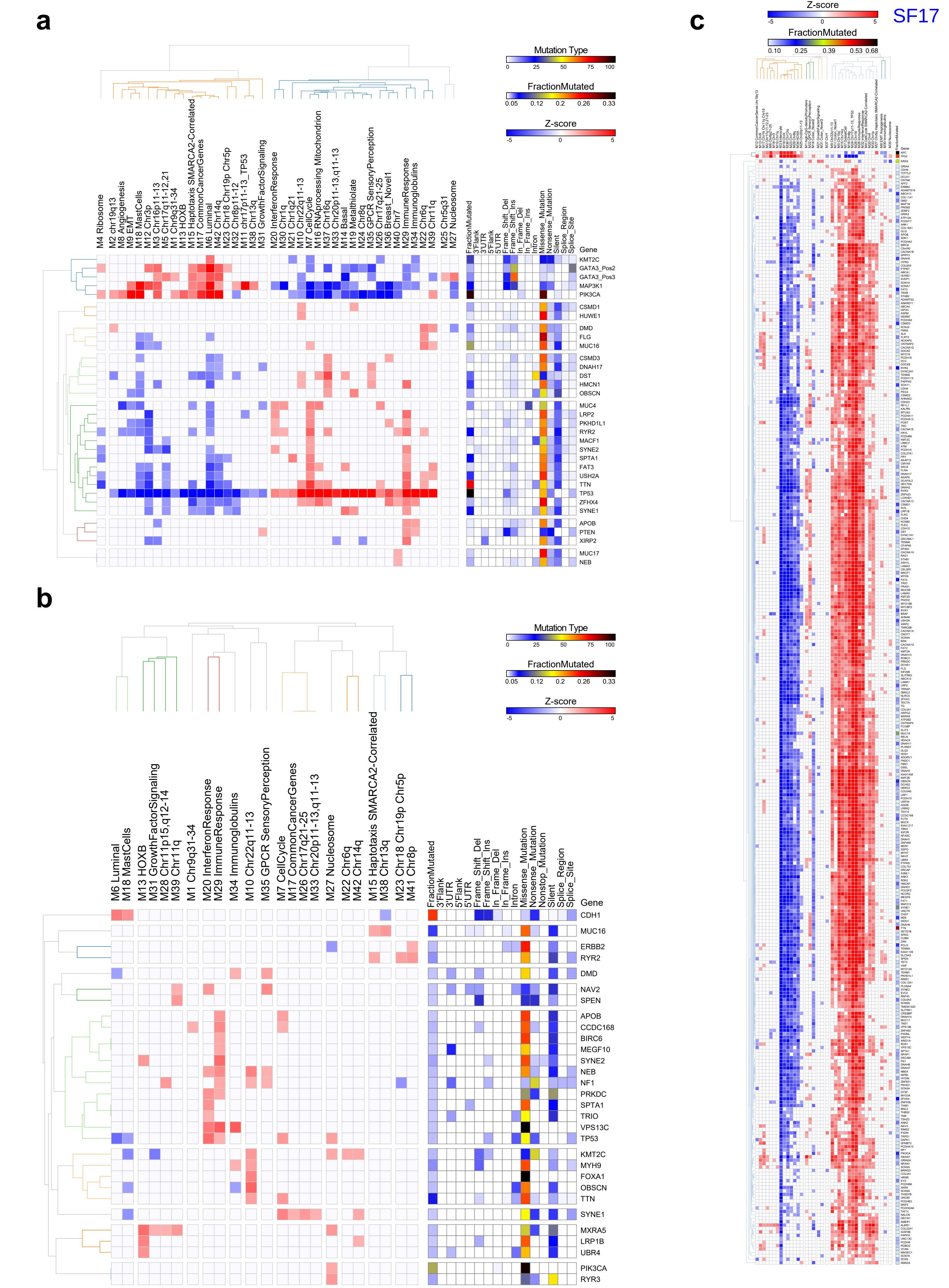

Supp. Fig.17. Network module and mutational association for BRCA divided by histological type and CRC. Accompanies Figure 6.
This Supp. Fig. Shows the results of analyzing the correlation between module expression values (MEV) and mutations in BRCA/CRC after separating cases according to histological type for (**a**) infiltrating ductal BRCA, (**b**) infiltrating lobular BRCA and (**c**) complete version of the correlations between module expression values (MEV) and mutation status for CRC, as shown in a truncated format in Fig. 6b. Significance of the Spearman's Rank correlation of MEV with mutation is illustrated as a z-score with the indicated blue to red scale, while for each gene the fraction of mutated cases and mutation type are illustrated with blue to black color code along the side of the heatmap. Heatmap shows hierarchical clustering for genes mutated in ≥ 5% BRCA & ≥ 10% CRC of TCGA patients.

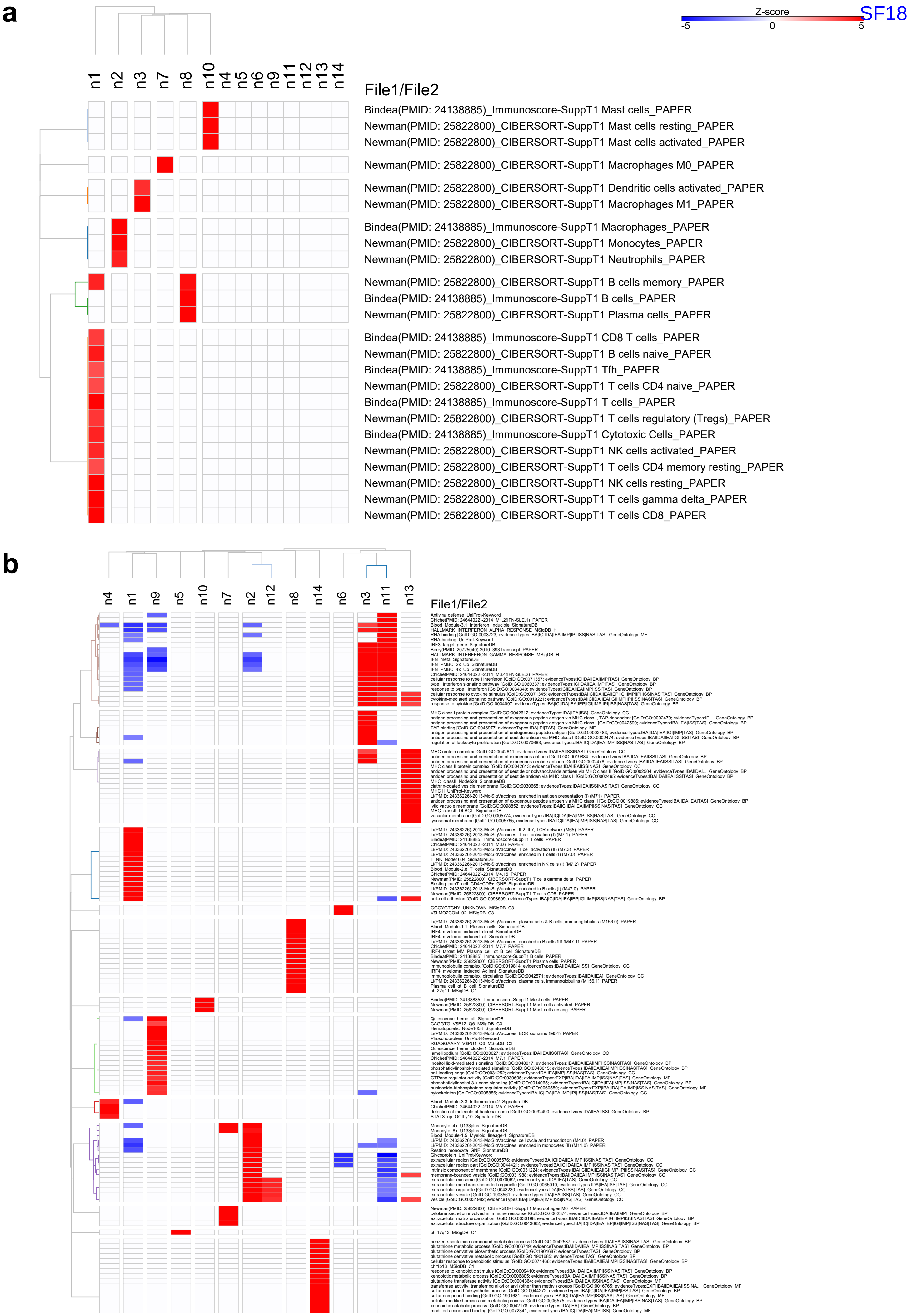

Supp. Fig.18. Gene signature and ontology enrichments in immune related neighborhoods in BRCA. Accompanies Figure 7.
These heatmaps illustrate the results of gene signature and ontology term enrichment analysis for the module neighborhood analysis shown in Figure 7 of the reclustering of BRCA modules M18, M20, M29 and M34. (**a**) CIBERSORT/Immunoscore signatures (**b**) select signatures: excluding MsigDB (C2,C4,C6 &C7) and GeneSigDB. Neighborhood numbers and designations are listed on the x-axis, and signature/ontology terms on the y-axis, modules were clustered according to gene signature enrichment using hierarchical clustering (using top 15 signatures per module; FDR <0.1). Gene signature enrichments are illustrated as a red/blue color code reflecting significance (z-score) of enrichment.

Contents

Motivation for method
Method development
- Generation of robust correlations
- Edge reduction
- Clustering methods
- Clustering selection
- Comparison of edge refinement methods
- Comparison with WGCNA

Additional Method Details
- Normalization and re-annotation of data
- Expression data sets
  - TCGA data sets
- Module stability
- Network visualization
- Network meta-data
- Module overlaps
- Application to TCGA data
  - Module Expression Values
  - Heatmap visualizations
  - Mutation correlation analysis

Statistical analyses
- Gene signature data and enrichment analysis
- Correlation of modules

Data processing
Data and software availability
References

Motivation for method

Our overall motivation was based on the following requirements for the method:

- Visualize/contextualize the biology contained in large gene expression data-sets.
- Generate modules (also referred to as clusters) that have distinct and meaningful biology using an unsupervised approach (no prior assumptions).
- Be able to map the discovered modules onto gene expression data-sets to generate module 'fingerprints' per sample/patient.

- Use these derived ‘fingerprints’ to study relationships between module biology and external factors (not expression based; e.g. mutations).
- Use module gene/signature membership to study recurrent features between cancers.

However, we wanted to achieve these goals whilst only introducing complexity where it was proven to be required. This was a core motivation for the development of the Parsimonious Gene Correlation Network Analysis (PGCNA) technique. There were several challenges that we needed to overcome during the implementation of PGCNA:

- Generation of robust correlations
- Edge reduction
- Clustering methods
- Clustering selection (ranking success)

These will be addressed in Method development and further specific method details given in Additional method details.

Method development

This section gives an overview of the development path of PGCNA.

Generation of robust correlations

See Supp. Fig.1 part 2 and 3

(For data-sets and data preparation see Normalization and re-annotation of data and Expression data sets)

In order to generate networks we required the correlations of all gene pairs within breast-cancer (BRCA) and colorectal-cancer (CRC). Sample biases may exist within a single gene expression data-set; in order to minimize such effects we decided to harness as many expression data-sets as possible. Merging the data-sets, given the diversity of array types and the resulting normalization issues would likely introduce noise. The simplest way to harness multiple data-sets was to initially analyze them independently and then merge the correlations. Per data-set the 80% most variant genes were retained, and Spearman’s rank correlations calculated (Python scipy.stats package). Given that each data-set was analyzed independently (different ranking of gene variance) and that the data-sets spanned array platforms (different gene content) the resulting correlation matrices whilst largely overlapping in terms of gene content, additionally included variable subsets of genes. The resultant *p*-values and correlations matrices were merged across all data sets for a given cancer by taking the median values (across the sets in which the gene pairs were contained) to give a final median correlation matrix and its corresponding *p*-value matrix. Genes present in < 9 and <4 data sets were removed from the BRCA and CRC matrices respectively. This gave a final matrix size of 17,805 and 18,896 for BRCA and CRC respectively. Finally, all correlations with a *p*-value > 0.05 were set to 0 to reduce noise.

Edge reduction

See Supp. Fig.1 part 4

One of the biggest challenges with gene network analysis is edge reduction (where edges are the gene pair correlations); the removal of less-informative correlations. As the (number of edges) = $(\text{number of genes})^2$ it quickly becomes intractable for both visualization and memory footprint (see Table 1). Using a hard-threshold, where correlations below a

cutoff are removed, can help reduce the total number of edges but at the cost of generating many orphan genes (disconnected from any other gene) thus failing our requirement to visualize/contextualize overall patterns of gene co-expression. Furthermore, in order to generate visually interpretable networks the generation of modules (finding modules within the correlation matrix) is sometimes separated from the visualization step, utilizing all edges for clustering while only visualizing a small subset (e.g. WGCNA; again accompanied by the problem of orphans)[1]. We aimed if possible to visualize all the data used in module discovery.

We decided to test the simplest possible edge reduction technique – For each gene (row) in a correlation matrix only the N most correlated Edges Per Gene (EPG; EPG3 means retaining 3 edges per gene) were retained, with N ranging from 3 to 10 (<3 gives orphan modules). The resulting matrix M, with entries written as $M = (m_{ij})$ was made symmetrical by setting $m_{ij} = m_{ji}$ for all indices i and j so that $M = M^T$ (its transpose). The idea being that related genes would join to form communities, in a fashion analogous to the assembly of a daisy-chain flower-garland.

We wanted to contrast the EPG approach with other edge refinement approaches. One such approach is applying an iterative Pearson's correlation coefficient (iPCC) where correlations are iteratively calculated using the previous iteration as input (1st – n^{th} order) until these converge on a correlation matrix that contains only -1/1 values[2]. In addition, we compared with two methods utilized by the WGCNA package: a power based (WGCNA *adjacency* function; referred here as PowerST) and sigmoid based (WGCNA *sigmoidAdjacencyFunction;* referred here as Sigmoid) function.

These different edge refinement approaches have different characteristics. While the iPCC method has been shown to aid module discovery it removes all substructure within modules and removes the opportunity for edge reduction (correlations all -1/1). While this may generate useful modules, by retaining such a large number of edges, and by removing information that may guide substructure within the modules, it fails our visualize/contextualize requirement. However, this was included in our analysis to test if clustering, at the cost of no visualization, would yield more informative modules.

The two WGCNA approaches (PowerST/Sigmoid) have user defined parameters, selection of these is guided by generating networks over a range of parameter values and quantifying how well each resultant network satisfies a scale-free topology. We analyzed PowerST/Sigmoid across the scale-free range to see how sensitive the results are to input parameters.

The EPG approach has two interesting properties – firstly even with just EPG3 no gene is an orphan and rarely is any set of genes disconnected from the remainder. By contrast at EPG2 orphan modules are often generated. Secondly, as some genes are common partners of many other genes the total number of edges < #genes x EPG, giving a degree (#edges per gene) that follows the power-law and is a scale-free network (see Supp. Fig. 2d, unlike hard-thresholding Supp. Fig. 2e)[3]. Furthermore, this edge reduction approach limits the final edge number to a maximum of #genes x EPG meaning that an 18,896 gene correlation matrix (2.7GB), that contains 178,519,960 non-redundant edges, can be reduced to 52,257 edges described by a 2.5MB file (see Table 1 for examples of edge reduction based on EPG3).

genes (nodes) vs matrix size relationship (Edge Per Gene=3)				
Genes	Total Nodes	Non Redundant Nodes	Total Size (GB)	Reduced Edge Estimate
1,000	1,000,000	499,500	0.01	3,000
10,000	100,000,000	49,995,000	0.75	30,000
20,000	400,000,000	199,990,000	2.99	60,000
30,000	900,000,000	449,985,000	6.73	90,000
40,000	1,600,000,000	799,980,000	11.96	120,000
50,000	2,500,000,000	1,249,975,000	18.69	150,000
60,000	3,600,000,000	1,799,970,000	26.92	180,000
70,000	4,900,000,000	2,449,965,000	36.64	210,000
80,000	6,400,000,000	3,199,960,000	47.85	240,000
90,000	8,100,000,000	4,049,955,000	60.56	270,000
100,000	10,000,000,000	4,999,950,000	74.77	300,000

Table 1: relationship between number of genes, edges and memory required to store original correlation matrix. Reduced edge estimate if retaining the top 3 edge per gene (EPG3).

Clustering methods

See Supp. Fig.1 part 5

The EPG edge reduction approach lends itself to network module discovery methods. We chose the 'Fast unfolding of communities in large networks'/Louvain modularity method (herein referred to as FastUnfold; version 0.3) as it scales to very large networks and runs fast enough to allow many iterations[4]. However, we wanted to make sure that our drastic edge reduction technique was not throwing away useful information. To this end we compared FastUnfold to two standard clustering approaches: hierarchical clustering and k-means clustering (R packages fastcluster and kmeans respectively), comparing these for EPG3 – EPG10 and the total unpruned correlation matrices. FastUnfold was run 10,000 times at each EPG level and the 100 best (judged by the FastUnfold modularity score) were used for downstream analysis. The FastUnfold algorithm automatically converges on a module number and therefore does not require a user defined module number. Interestingly, the median number of modules generated by FastUnfold decreased with EPG level (Supp. Fig.2a), converging on 3-5 modules for the unpruned total correlation matrix. Thus, applying FastUnfold was not particularly effective when considering the unpruned data. By contrast the generation of discrete modules by FastUnfold, which penalizes network solutions that retain connectivity between modules, is favored by the EPG edge reduction technique. The latter intrinsically selects for hub nodes and favors separation of gene clusters by ensuring that common partner genes at the given EPG level retain more edges. This could be seen as effectively pre-focusing the input correlation matrix prior to application of the network module discovery method.

For the k-means clustering k was set to ± 1 around the module number from the best FastUnfold solution (see Cluster selection) and for each k and EPG 50 iterations were run.

For hierarchical clustering 8 different linkage methods (average, centroid, complete, Mcquitty, Median, Single, WardD and WardD2) were used and the resultant dendrograms cut at ± 10 around the module number from the best FastUnfold solution, giving 21 results for every input matrix (note: only the 2 best linkage methods, WardD/WardD2, are shown in Fig. 1a).

Clustering selection

See Supp. Fig.1 part 6

To judge the success of the clustering approaches we wanted a metric that assessed 3 things:

- Biological enrichment in each module.
- Purity of biological enrichment. Rewarding clustering approaches that separate genes into modules with distinct biological function, and in turn punishing those that have functional redundancy across modules.
- Skewing of modules sizes. While we don't expect/require all modules to be the same size we want to avoid skewing as much as possible (i.e. a few modules that contain many genes/functions).

Gene signature analysis was carried out for each module, from each clustering of the data. Then to generate a total enrichment score for a given clustering:

1. Signatures were filtered to retain only those with ≥ 5 and ≤ 1000 genes with a false discovery rate (FDR; Benjamini Hochberg) of < 0.05.
2. For each module within a clustering, the enriched signatures were ranked by FDR and the top 15 added to a global list of signatures for that clustering.
3. A matrix was generated that contained all the z-scores for every signature (rows) in the global list across all the modules (columns).
4. For each signature a fractional contribution was calculated as the row-max-zScore/row-sum-zScores (where 1 = enrichment of signature in only 1 module). Across all signatures a median factional contribution (MFC) was calculated.
5. The sum of the maximum z-score per signature (row) was calculated (ZScoreMS).
6. Module size skewing was assessed by calculating the normalized Shannon entropy:

$$H_n(p) = -\sum_i \frac{p_i \log_b p_i}{\log_b n}$$

   of the module sizes. This gave a score that ranged from 1 (even module sizes) towards 0 with increasing skewing.

Since both MFC and normalizedEntropy have a range of 0—1 the simplest way to scale the ZScoreMS was the product of the three values, thus:

Scaled cluster enrichment score (SCES) = ZScoreMS · MFC · normalizedEntropy.

This allowed the selection of the best FastUnfold clustering (Fig.1a; Gene Signature Enrichment: FastUnfold). This was then used to set the module number range in the k-means/hierarchical approaches. The FastUnfold method outperformed the k-means/hierarchical clustering methods across all EPG, with only the Ward-linkage hierarchical clustering approaching a similar enrichment when using the Total data. With increasing EPG there was a corresponding decrease in module number with no trade-off of increased biological enrichment (Supp Fig.2a and Fig. 1a). Thus, for all downstream analysis we chose the optimal FastUnfold EPG3 result for both cancers. However, it should be noted that most of the recovered modules were broadly retained across the 100 FastUnfold clustering results (see Module Stability and Supp. Fig. 2b/c). The combination of FastUnfold and EPG3 we term a Parsimonious Gene Correlation Network Analysis (PGCNA).

Fig. 3a/b and Supp. Fig. 11a/b show visualizations of the optimal BRCA/CRC gene signature results. As before these show the top 15 signatures per module (with ≥ 5 and ≤ 1000 genes) but are filtered with the more lenient p-value < 0.01.

Comparison of edge refinement methods

The EPG edge reduction strategy combined with clustering with FastUnfold (termed PGCNA) generated modules with high Scaled cluster enrichment scores (SCES). Given the simplicity of the EPG approach it might be surprising that it generates such good results. We therefore used 3 other edge refinement approaches (iPCC, PowerST and Sigmoid; see Edge Reduction) in combination with FastUnfold as a comparison.

As the PowerST and Sigmoid function utilized WGCNA, and we wanted to also compare against the whole WGCNA package (Comparison with WGCNA) we could not use the median correlation matrices for this analysis. This is because WGCNA is mostly aimed at analyzing individual data-sets starting from gene expression arrays. While the median correlation matrix we used for the PGCNA analysis could be imported into WGCNA this then precluded many of the downstream features. Thus, we selected single representative data-sets for BRCA/CRC to analyze, selecting two data-sets that were on the Affymetrix Human Genome U133 Plus 2.0 platform and had been used successfully to generate classifications of BRCA/CRC (GSE20686/GSE39582 containing 327 and 566 samples respectively).

For both data-sets, probes were reannotated and merged (see Normalization and re-annotation of data) and the 80% most variant genes retained, giving data-sets containing 16,807 genes.

For both the BRCA and CRC data-sets the edge reduction strategies:

- EPG3 (Edge Per Gene 3)
- iPCC (iterative Pearson's correlation coefficient): converged to -1/1 (12th order BRCA, 11th order CRC)
- PowerST: power 2—10; covering the scale-free range (see Supp. Methods Fig.1. a/b)
- Sigmoid: μ 0.1—0.9; covering the scale-free range (not shown)

Were clustered by FastUnfold 10,000 and the best 100 (judged by FastUnfold modularity score) were retained for downstream analysis.

The different methods were clustered into varying numbers of modules across a range of SCES (Fig. 1. b/c and Supp. Fig. 3 and 4). The iPCC, retaining > 70 million edges, always clustered into 2 modules, with the lowest level of SCES. PowerST generated between 2—4 modules, with marginally more SCES than iPCC. The Sigmoid function generated 3—280.5 and 3.5—263 median modules for BRCA and CRC respectively. As can be seen in Supp. Fig. 2/3 as the sigmoid μ increased a larger portion of very small modules was produced, so that by 0.9 only 38 of the 280.5 and 33 of the 263 modules had > 5 genes for BRCA/CRC respectively. In addition, if the resulting correlation matrices were cut at a hard threshold of 0.01 then only 32/61 % of genes were connected in the Sigmoid0.9 networks for BRCA/CRC respectively.

The EPG3 method generated networks that had the highest SCES and lowest edge numbers whilst maintaining 100% gene connectivity. For example, BRCA EPG3 generated a median of 31 modules with an SCES of 6,754 with only 46,776 edges, in contrast

a

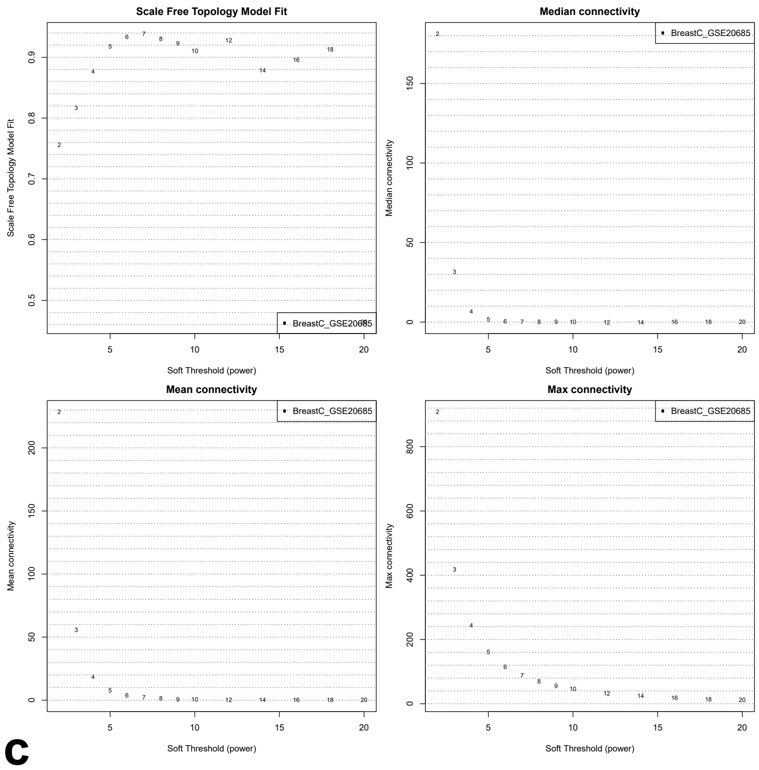

b

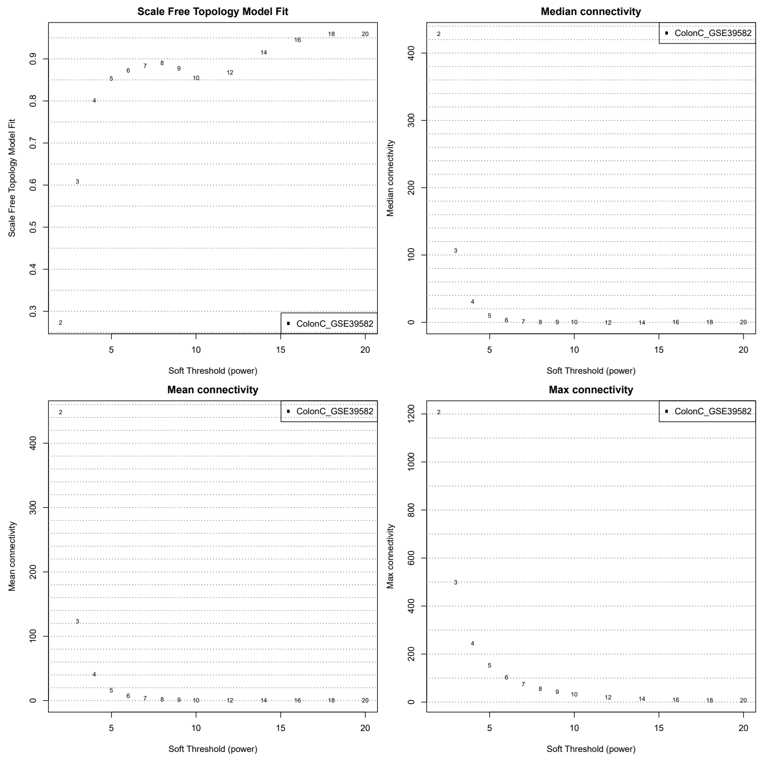

c

	SoftThreshold	MemoryUsage (GB)	RunTime	TotalModules	NumberInModule0
GSE20685	2	20.340G	00:13:29	34	6523
	3	23.902G	00:21:10	32	6765
	4	23.918G	00:20:41	22	7197
	5	19.850G	00:15:30	20	8199
	6	19.588G	00:13:12	18	8974
	7	19.657G	00:14:03	21	9842
GSE39582	2	29.471G	00:19:46	43	1323
	3	24.727G	00:13:29	56	814
	4	24.161G	00:13:50	57	910
	5	20.363G	00:12:38	48	1476
	6	20.751G	00:13:26	55	2534
	7	20.457G	00:12:45	59	3532

Supp. Methods Fig.1. **WGCNA run information**. Analysis of network topology for various soft-thresholding powers for (**a**) BRCA and (**b**) CRC. In each panel the (x-axis) shows the soft-threshold power as a function of scale free topology model fit (top-left panel), median-connectivity (top-right panel), mean-connectivity (bottom-left panel) and max-connectivity (bottom-right panel). (**c**) table showing various run statistics for the different soft-thresholds used, MemoryUsage: maximum GB of RAM used, RunTime: total time taken to run WGCNA, TotalClusters: final number of modules generated, NumberInModule0: number of genes in grey/0 (unassigned) module.

Sigmoid0.9 generated a median of 280.5 modules (38 with >5 genes) with an SCES of 5,044 with 189,401 edges, but with only 32% of genes connected.

Thus, the EPG edge reduction approach seems to be particularly suited to clustering by FastUnfold.

Comparison with WGCNA

In order to assess any potential added value of the PGCNA approach we considered its relative merits in relation to the popular gene network analysis approach, the Weighted Gene Correlation Network Analysis (WGCNA) tool[1]. It tackles the edge reduction problem in two ways – firstly the correlation matrix is raised to a power (soft-thresholding; default approach), with the power chosen to try and maximize the scale-free nature of the network, this has the effect of reducing low correlations to near zero. Secondly, after soft-thresholding the correlation matrix is converted to a topological overlap measure matrix (TOM). This ranges from 1 (direct link between two genes with one set of direct neighbors being a subset of the other) to 0 (no direct link between two genes). TOM has been shown to aid module discovery and help with spurious/missing correlations between genes. The TOM is used for module discovery using a hierarchical clustering approach followed by merging of similar modules (via clustering of eigengenes) to end up with a representative set of modules.

Where possible we used the default settings for WGCNA:

- Network-type: signed
- Cut-method: tree (cutreeDynamic)
- Soft-Power: 2—7 (covering the scale free range for both BRCA/CRC; see Supp. Methods Fig. 1 a/b)
- Min module size: 20 (default for cutreeDynamic)
- Deep split : True
- Merge cutHeight: 0.2 (default for mergeCloseModules)

For the PGCNA analysis the same expression data-sets were used to generate Spearman correlation matrices. These were pruned by EPG 3—10 and clustered 10,000 times with FastUnfold, the best 100 (judged by FastUnfold modularity score) were retained for downstream analysis (see Supp. Methods Fig. 2).

Comparing SCES levels across the 100 PGCNA EPG3 clusterings and 6 WGCNA soft-threshold levels (Supp. Fig. 5) showed that the PGCNA approach generated better overall biological enrichment, which is less redundant across modules and had modules that were more even in size (Supp. Fig. 5. a). There was a clear separation between the distribution of enrichments when comparing these methods, such that even the worst of the 100 iterations of the FastUnfold clusterings outperformed the best WGCNA derived clustering.

Comparing modules obtained across the different run parameters addresses to what extent gene membership of modules is a recurrent feature. For these comparisons a hypergeometric test was used to compare the overlap of module gene membership generated by the indicated networking solution (WGCNA and PGCNA). For WGCNA the comparisons shown are between different soft threshold levels (ST1-ST7) (Supp. Fig. 6/7), while for PGCNA either different FastUnfold solutions based on the input EPG level are compared (EPG3-EPG10) or for EPG3 the network solutions with Best, Median and Worst resolution of gene ontologies as judged by the scaled cluster enrichment score (SCES) are displayed (Supp. Fig. 8/9). WGCNA has a greater disparity between soft-threshold levels

Merged Data-set analysis (median correlation matrices)										
**Data-set**	**Samples**	**Genes**	**Total Edges**	**Non Redundant Edges**	**Total Size (GB)**	**Reduced Edge Estimate**	**Actual Reduced Edges**	**Time Taken**	**RAM used**	**FastUnfold**
BRCA	NA	17,805	317,018,025	158,500,110	2.37	53,415	49,199	NA	NA	10,000 pick 100
CRC	NA	18,896	357,058,816	178,519,960	2.67	56,688	52,257	NA	NA	10,000 pick 100
**Single Data-set analysis**										
GSE39582 (CRC) EPG3						50,421	45,726	00:19:31	833.742MB	1,000 pick 5
GSE39582 (CRC) EPG4						67,228	60,709	00:20:32	833.629MB	1,000 pick 5
GSE39582 (CRC) EPG5						84,035	75,544	00:20:14	833.629MB	1,000 pick 5
GSE39582 (CRC) EPG6	566					100,842	90,387	00:20:57	832.957MB	1,000 pick 5
GSE39582 (CRC) EPG7						117,649	105,149	00:20:30	832.957MB	1,000 pick 5
GSE39582 (CRC) EPG8						134,456	119,867	00:21:15	833.000MB	1,000 pick 5
GSE39582 (CRC) EPG9						151,263	134,601	00:23:03	832.898MB	1,000 pick 5
GSE39582 (CRC) EPG10		16,807	282,475,249	141,229,221	2.11	168,070	149,213	00:22:22	832.961MB	1,000 pick 5
GSE20685 (BRCA) EPG3						50,421	46,776	00:17:38	581.160MB	1,000 pick 5
GSE20685 (BRCA) EPG4						67,228	62,267	00:21:39	583.145MB	1,000 pick 5
GSE20685 (BRCA) EPG5						84,035	77,675	00:21:23	581.699MB	1,000 pick 5
GSE20685 (BRCA) EPG6	327					100,842	93,067	00:21:41	579.301MB	1,000 pick 5
GSE20685 (BRCA) EPG7						117,649	108,428	00:21:13	581.066MB	1,000 pick 5
GSE20685 (BRCA) EPG8						134,456	123,790	00:21:53	579.293MB	1,000 pick 5
GSE20685 (BRCA) EPG9						151,263	139,145	00:21:36	579.387MB	1,000 pick 5
GSE20685 (BRCA) EPG10						168,070	154,448	00:22:14	579.355MB	1,000 pick 5

Supp. Methods Fig.2. **PGCNA run information**. Top table shows results for the merged data-set analyses, the bottom table the results for the single data-set analyses over a range of EPG. For the single data-set analyses to make the results more comparable with WGCNA the Time-Taken is for performing 1,000 FastUnfold clusterings, while 10,000 were used in the final analysis (~ 2h run time). In addition, the Time-Take and RAM-used are not shown for the Merged data-sets (top table) as these were generated before the final PGCNA python script was produced (see software for details on python script).

than PGCNA does for either EPG or GSE levels, which is shown by the WGCNA histograms having a skewing to low level $-\log_{10}$ *p*-values (high *p*-values) and PGCNA skewing to high $-\log_{10}$ *p*-values. PGCNA generates very similar sets of modules across EPG levels, with higher EPG modules being supersets of those generated with lower EPG. In addition, the difference between the best/worst PGCNA clustering (judged by SCES; see grey boxes in Supp. Fig. 8/9) is less than seen between WGCNA soft-threshold levels, and again reflects modules being split/joined rather than completely distinct modules being identified.

This analysis suggests that for the data-sets tested PGCNA outperforms the popular WGCNA method. It is likely that the WGCNA approach could be improved with fine tuning of parameters (e.g. different cut/merge heights), selection of a more robust clustering method (biweight mid-correlation) etc. However, we note that a strength of the PGCNA approach is again provided by simplicity, which by minimizing user options removes the onus to fine-tune parameters, while still providing efficient network solutions. PGCNA users may choose to retain more edges as one variable input parameter, we note that increasing EPG level of the parsimonious correlation matrix primarily has the effect of merging modules that separate at more stringent EPG levels, rather than leading to the identification of entirely new gene clustering.

As an extension of this concept FastUnfold resolves substructure within network modules. Such substructure can be further explored within individual modules or across merged combinations of modules, or indeed by focusing around a single index gene by limiting the content of the input correlation matrix. This is illustrated in the main manuscript in what we refer to as a neighborhood analysis. Here the computational efficiency of the EPG and FastUnfold combination again proves valuable.

Thus, overall, the EPG method of edge reduction, while simple, when combined with FastUnfold yields surprisingly robust results that:

- Outperform analyzing all edges with existing clustering methods (hierarchical-clustering/k-means; see Fig.1 a).
- Outperform other edge refinement methods combined with FastUnfold (see Fig. 1. b/c and Supp. Fig. 3/4).
- Outperform more computationally expensive networking approaches (e.g. WGCNA, see Supp. Fig. 5-9).
- Allows visualization/storage of very large networks by reducing to a minimal edge set.
- Allows visualization of the same data that was used for clustering. To visualize WGCNA networks there is often a requirement to carry out additional hard thresholding (removing edges < correlation-cutoff) to reduce to a tractable edge number, thus the clustered/visualized data are different.
- Generates networks with scale-free properties, with hub nodes that seem to be recurrent between cancers (e.g. TNFSF13B, ARHGAP9) and stable across analysis of individual data-sets.

Additional Method Details

This section gives specific details on the upstream processing of data that was used in PGCNA generation and the downstream analysis of the output from the PGCNA networks.

Normalization and re-annotation of data

For each data set the probes were re-annotated using the MyGene.info (http://mygene.info) API using all available references (e.g. NCBI Entrez, Ensembl etc.) and any ambiguous mappings manually assigned [5].

Each data set was quantile normalized using the R Limma package and the probes for each gene merged by taking the median value for probe sets with a Pearson correlation ≥0.2 and the maximum value for those with a correlation <0.2 (as used by Monti *et al*) [6,7].

Expression data sets

See Supp. Fig.1 part 1

For the generation of the gene correlation networks 23 breast cancer (BRCA) and 12 colorectal cancer (CRC) gene expression data sets were downloaded from the Gene Expression Omnibus[8] (BRCA, 7464 cases; 26 arrays) [9–30] (CRC, 2399 cases; 11 arrays after merging 2) [31–40]. Three of the BRCA data sets were on two different expression platforms (GSE3494, GSE36774 and GSE4922), these were analyzed independently. In the case of CRC two related data sets were merged (GSE17536), giving a total of 11 CRC data sets covering 2,399 samples (Table 2).

Type	GEO Code	ChipNumber	Size
BRCA Network-Generation	GSE12276	570	204
	GSE16201	8583	403
	GSE20194	96	278
	GSE20271	96	178
	GSE20685	570	327
	GSE21653	570	266
	GSE22093	96	103
	GSE22219	6098	216
	GSE22820	6480	176
	GSE24450	6947	183
	GSE25066	69	508
	GSE25307	5345	577
	GSE26639	570	226
	GSE2990	96	189
	GSE30682	6884	343
	GSE31448	570	353
	GSE3494	96	251
	GSE3494	97	251
	GSE36774	96	149
	GSE36774	570	107
	GSE45725	6883	340

Type	GEO Code	ChipNumber	Size
CRC Network-Generation	GSE17536	570	177
	GSE17537	570	55
	GSE26682	96	331
	GSE26682	570	331
	GSE28722	13425	125
	GSE37892	570	130
	GSE38832	570	122
	GSE39582	570	566
	GSE42284	16280	171
	GSE44076	13667	98
	GSE5206	570	105
	GSE68468	96	188
		Total	2399

GSE4611	96	218
GSE48091	10379	623
GSE4922	96	289
GSE4922	97	289
GSE54002	570	417
	Total	7464

Table 2: Data-sets used for network generation. For BRCA the data-sets highlighted in bold were available on two different platforms, for CRC the two data-sets highlighted in bold were merged.

TCGA data sets

For independent assessment of the network modules two RNA-seq data sets were downloaded from The Cancer Genome Atlas (BRCA/CRC data sets were downloaded on 2017.11.15 from http://cancergenome.nih.gov/) along with the corresponding simple nucleotide variation data (MuTect2 pipeline). The individual level 3 RNA-seq data were merged, quantile normalized and re-annotated using the MyGene.info (http://mygene.info) API using all available references (e.g. NCBI Entrez, Ensembl etc.) and any ambiguous mappings manually assigned [5]. The overlapping expression/mutation samples were used for downstream analyses.

Network-Assessment (TCGA data)

Data-set	RNA-seq #	Mutation #	Shared
BRCA (Female)	1079	1033	1027
COAD(CRC)	456	422	429

Module stability

The stability of modules was assessed to see how recurrent the modules were across different clustering runs (Supp. Fig. 2b/c). Using the optimal clustering as a reference, for each of the 100 FastUnfold clustering, per reference module:

1. Find the maximum overlapping module.
2. Store the number of overlapping genes along with significance (*p*-value) of the overlap and increment sums for the overlapping genes.

The stability % per gene is simply the overlap sum (i.e. across 100 clustering runs what % of maximum overlapping modules is the gene found in). The stability values per reference module was calculated as median overlap across the 100 clustering runs.

Network visualization

See Supp. Fig.1 part 7

The optimal EPG3 matrix from BRCA/CRC was converted into a list of edges and nodes and uploaded into the Gephi package (version 0.9.2) [41]. Modules were colored so that were possible significantly overlapping modules between BRCA and CRC shared colors. Degree and Betweenness Centrality were calculated, and the latter used to adjust node sizes. The network layout was generated using the ForceAtlas2 approach, and interactive HTML5 web visualizations exported using the sigma.js library (https://github.com/oxfordinternetinstitute/gephi-plugins/tree/sigmaexporter-plugin).

Network meta-data

See Supp. Fig.1 part 8

A number of additional features were calculated for the network genes across the data sets used to generate the correlation network. For each gene the median percentile expression was calculated across all data sets, its dispersion across data-sets calculated as the median absolute deviation (MAD) and its dispersion within data-sets (i.e. across patients) calculated as the median quantile coefficient of dispersion (QCOD).

The Survival library for R was used to analyze right-censored survival data for the data sets where this was available (n=8 for BRCA, n=4 for CRC). Within each data set the expression of each gene (as z-score) was used as a continuous variable in a Cox Proportional Hazards model. Across data sets a meta-analysis was conducted by fitting a fixed-effect model (R metafor package) to the hazard ratios, weighted by data set size.

rma(yi=lnHazardRatio,sei=standardErr,weights=dataSetSize,weighted=TRUE,method="FE") [42].

Module overlaps

See Supp. Fig.1 part 9

The overlap of the modules between cancers at the gene and signature level was assessed using a hypergeometric test and the overlap visualized as a python matplotlib heatmap of -$\log_{10}$ *p*-values (Fig. 3c/d), and with the overlap number and module size displayed (Supp. Fig. 11c/d). The signatures were pre-filtered to *p*-value <0.001 and ≥ 5 and ≤ 1000 genes.

Application to TCGA data

The modules derived from the GEO 'training data' were used to analyze unseen expression and mutation data from The Cancer Genome Atlas (TCGA).

Module Expression Values

See Supp. Fig.1 part 10

To assign module enrichment/depletion at the patient level a summary score was created for each module for each patient.

Within each data set, which vary in available genes, the first step was to select the 25 most representative genes per module:

1. For every gene a connectivity score was calculated by summing its correlations within its module.
2. This was then weighted using expression and dispersion information

   $$\text{ModCon} = \text{connectivity}^2 \cdot \text{percentileExpression} \cdot \text{VarWithin} \cdot (100 - \text{VarAcross})/100$$

   Where VarWithin is the dispersion of a gene expression within data sets measured as the median quantile coefficient of dispersion (max range 0—1), VarAcross is the dispersion of gene expression across data sets measured as the median absolute deviation of percentile expression (max range 0—100). This rewards genes that have high connectivity and are variant across patients but invariant across data sets.
3. Genes were ranked by ModCon and the top 25 selected.

These 25 genes were then converted to a Module Expression Value (MEV):

1. Per gene, standardize (z-score) the quantile normalized $\log_2$ expression data.
2. Per sample (patient) sum the 25 z-scores to give a MEV.

Heatmap visualizations

See Supp. Fig.1 part 11

The MEV were used to create heatmap visualizations of each module at the patient level within the BRCA and CRC TCGA data sets. Using the Broad GENE-E package (https://software.broadinstitute.org/GENE-E/) the MEV were hierarchically clustered (Pearson correlations and average linkage) and displayed along with available meta data (Supp. Fig. 15/16).

Mutation correlation analysis

See Supp. Fig.1 part 12

The relationship of mutations and modules was calculated using the MuTect2 simple nucleotide variation (SNV) mutation data and the MEV. The SNV data was filtered to retain mutations present in > 5 or > 10% of patients in BRCA and CRC respectively. Spearman's rank correlations were calculated between all pairs of mutated gene and module. These were converted to z-scores to convey the ± correlation along with its significance. A matrix was output containing the z-scores for all gene/modules ≥ 1 positive significant (*p*-value <0.05) correlation (i.e. a gene need only be significant in one module to be included). This matrix was then hierarchically clustered (Pearson correlations and average linkage) using GENE-E (Fig. 6/7 and Supp. Fig.17).

For BRCA the 140 GATA3 mutations were split into 3 groups based on mutation position: GATA3_Pos1 (Chr10: 8058419—8064131; n=10), GATA3_Pos2 (Chr10: 8069470—8069596; n=57) and GATA3_Pos3 (Chr10: 8073734—8074229; n=73).

Statistical analyses

Gene signature data and enrichment analysis

A data set of 17,211 gene signatures was created by merging signatures downloaded from http://lymphochip.nih.gov/signaturedb/ (SignatureDB), http://www.broadinstitute.org/gsea/msigdb/index.jsp MSigDB V6.1 (MSigDB C1–C7 and H; excluding C5. With MIPS signatures from version 3.1 and PID signatures from version 4 added back), http://compbio.dfci.harvard.edu/genesigdb/ Gene Signature Database V4 (GeneSigDB), UniProt keywords (parsed XML from http://www.uniprot.org/downloads), and fifteen papers [6,36,43–58]. A gene ontology gene set was created using an in-house python script. This parses a gene association file (http://geneontology.org/page/download-go-annotations) to link genes with ontology terms and then uses the ontology structure (.obo file; http://purl.obolibrary.org/obo/go.obo) to propagate these terms up to the root. The resultant gene set contained 22,271 terms. The gene-ontology and gene-signatures sets were merged to give a final signature set of 39,482 terms.

Enrichment of gene lists for signatures was assessed using a hypergeometric test, in which the draw is the gene list genes, the successes are the signature genes, and the population is the genes present on the platform.

Correlation of modules

The relationship of the modules was analyzed by calculating the Spearman's rank correlation for all module (as MEV) pairs within each data set. These were then merged across data sets by calculating the median correlation and *p*-values. A final matrix generated by setting all correlations with a *p*-value > 0.05 to 0. Within GENE-E the 'training data' was hierarchically clustered (Pearson correlations and average linkage) and the TCGA data displayed in the same order without hierarchical clustering (Supp. Fig.14).

Data processing

All analyses were undertaken on MARC1, part of the High Performance Computing and Leeds Institute for Data Analytics (LIDA) facilities at the University of Leeds, UK.

Data and software availability

Interactive networks and all meta-data are available at http://pgcna.gets-it.net/.

PGCNA python scripts are available at https://bitbucket.org/mcare/pythonscripts-pgcna/src. The PGCNA python program has been implemented to store data using the HDF5 data format. This allows a very small memory footprint (<1GB) even when analyzing very large data-sets. For example, running a 20,000 gene data-set (3GB correlation data-file) with 1000 clusterings takes ~20 minutes, however, even an 180,000 node data-set (240GB correlation data-file) can be run in <1GB or RAM, taking ~13 hours to run (including 1000 FastUnfold clusterings). This large data-set is reduced from 16 billion edges to 540,000 (43MB), which is possible to render using Gephi V0.9.2.

References

1. Langfelder, P. & Horvath, S. WGCNA: an R package for weighted correlation network analysis. *BMC Bioinformatics* 9, 559 (2008).

2. Ren, X., Wang, Y., Zhang, X.-S. & Jin, Q. iPcc: a novel feature extraction method for accurate disease class discovery and prediction. *Nucleic Acids Res.* 41, e143 (2013).

3. Alstott, J., Bullmore, E. & Plenz, D. Powerlaw: a Python package for analysis of heavy-tailed distributions. *PLoS One* 9, e85777 (2014).

4. Blondel, V. D., Guillaume, J.-L., Lambiotte, R. & Lefebvre, E. Fast unfolding of communities in large networks. *J. Stat. Mech. Theory Exp.* 2008, P10008 (2008).

5. Xin, J. *et al.* High-performance web services for querying gene and variant annotation. *Genome Biol.* 17, 91 (2016).

6. Monti, S. *et al.* Molecular profiling of diffuse large B-cell lymphoma identifies robust subtypes including one characterized by host inflammatory response. *Blood* 105, 1851–61 (2005).

7. Ritchie, M. E. *et al.* limma powers differential expression analyses for RNA-sequencing and microarray studies. *Nucleic Acids Res.* 43, e47 (2015).

8. Barrett, T. *et al.* NCBI GEO: archive for functional genomics data sets--10 years on. *Nucleic Acids Res.* 39, D1005-10 (2011).

9. Bos, P. D. *et al.* Genes that mediate breast cancer metastasis to the brain. *Nature* 459, 1005–9 (2009).

10. Roepman, P. *et al.* A gene expression profile for detection of sufficient tumour cells in breast tumour tissue: microarray diagnosis eligibility. *BMC Med. Genomics* 2, 52 (2009).

11. Hatzis, C. *et al.* A genomic predictor of response and survival following taxane-anthracycline chemotherapy for invasive breast cancer. *JAMA* 305, 1873–81 (2011).

12. Jönsson, G. *et al.* The retinoblastoma gene undergoes rearrangements in BRCA1-deficient basal-like breast cancer. *Cancer Res.* 72, 4028–36 (2012).

13. de Cremoux, P. *et al.* Importance of pre-analytical steps for transcriptome and RT-qPCR analyses in the context of the phase II randomised multicentre trial REMAGUS02 of neoadjuvant chemotherapy in breast cancer patients. *BMC Cancer* 11, 215 (2011).

14. Sotiriou, C. *et al.* Gene expression profiling in breast cancer: understanding the molecular basis of histologic grade to improve prognosis. *J. Natl. Cancer Inst.* 98, 262–72 (2006).

15. Servant, N. *et al.* Search for a gene expression signature of breast cancer local recurrence in young women. *Clin. Cancer Res.* 18, 1704–15 (2012).

16. Sabatier, R. *et al.* Down-regulation of ECRG4, a candidate tumor suppressor gene, in human breast cancer. *PLoS One* 6, e27656 (2011).

17. Miller, L. D. *et al.* An expression signature for p53 status in human breast cancer predicts mutation status, transcriptional effects, and patient survival. *Proc. Natl. Acad. Sci. U. S. A.* 102, 13550–5 (2005).

18. Wang, D.-Y., Done, S. J., Mc Cready, D. R. & Leong, W. L. Validation of the prognostic gene portfolio, ClinicoMolecular Triad Classification, using an independent prospective breast cancer cohort and external patient populations. *Breast Cancer Res.* 16, R71 (2014).

19. Karn, T. *et al.* Data-driven derivation of cutoffs from a pool of 3,030 Affymetrix arrays to stratify distinct clinical types of breast cancer. *Breast Cancer Res. Treat.* 120, 567–79 (2010).

20. Cunha, S. I. *et al.* Endothelial ALK1 Is a Therapeutic Target to Block Metastatic Dissemination of Breast Cancer. *Cancer Res.* 75, 2445–56 (2015).

21. Popovici, V. *et al.* Effect of training-sample size and classification difficulty on the accuracy of genomic predictors. *Breast Cancer Res.* 12, R5 (2010).

22. Ivshina, A. V *et al.* Genetic reclassification of histologic grade delineates new clinical subtypes of breast cancer. *Cancer Res.* 66, 10292–301 (2006).

23. Tan, T. Z. *et al.* Epithelial-mesenchymal transition spectrum quantification and its efficacy in deciphering survival and drug responses of cancer patients. *EMBO Mol. Med.* 6, 1279–93 (2014).

24. Tabchy, A. *et al.* Evaluation of a 30-gene paclitaxel, fluorouracil, doxorubicin, and cyclophosphamide chemotherapy response predictor in a multicenter randomized trial in breast cancer. *Clin. Cancer Res.* 16, 5351–61 (2010).

25. Kao, K.-J., Chang, K.-M., Hsu, H.-C. & Huang, A. T. Correlation of microarray-based

breast cancer molecular subtypes and clinical outcomes: implications for treatment optimization. *BMC Cancer* 11, 143 (2011).

26. Sabatier, R. *et al.* A gene expression signature identifies two prognostic subgroups of basal breast cancer. *Breast Cancer Res. Treat.* 126, 407–20 (2011).

27. Iwamoto, T. *et al.* Gene pathways associated with prognosis and chemotherapy sensitivity in molecular subtypes of breast cancer. *J. Natl. Cancer Inst.* 103, 264–72 (2011).

28. Buffa, F. M. *et al.* microRNA-associated progression pathways and potential therapeutic targets identified by integrated mRNA and microRNA expression profiling in breast cancer. *Cancer Res.* 71, 5635–45 (2011).

29. Liu, R.-Z. *et al.* Association of FABP5 expression with poor survival in triple-negative breast cancer: implication for retinoic acid therapy. *Am. J. Pathol.* 178, 997–1008 (2011).

30. Heikkinen, T. *et al.* Variants on the promoter region of PTEN affect breast cancer progression and patient survival. *Breast Cancer Res.* 13, R130 (2011).

31. Smith, J. J. *et al.* Experimentally derived metastasis gene expression profile predicts recurrence and death in patients with colon cancer. *Gastroenterology* 138, 958–68 (2010).

32. Vilar, E. *et al.* MRE11 deficiency increases sensitivity to poly(ADP-ribose) polymerase inhibition in microsatellite unstable colorectal cancers. *Cancer Res.* 71, 2632–42 (2011).

33. Loboda, A. *et al.* EMT is the dominant program in human colon cancer. *BMC Med. Genomics* 4, 9 (2011).

34. Laibe, S. *et al.* A seven-gene signature aggregates a subgroup of stage II colon cancers with stage III. *OMICS* 16, 560–5 (2012).

35. Tripathi, M. K. *et al.* Nuclear factor of activated T-cell activity is associated with metastatic capacity in colon cancer. *Cancer Res.* 74, 6947–57 (2014).

36. Marisa, L. *et al.* Gene expression classification of colon cancer into molecular subtypes: characterization, validation, and prognostic value. *PLoS Med.* 10, e1001453 (2013).

37. Roepman, P. *et al.* Colorectal cancer intrinsic subtypes predict chemotherapy benefit, deficient mismatch repair and epithelial-to-mesenchymal transition. *Int. J. cancer* 134, 552–62 (2014).

38. Solé, X. *et al.* Discovery and validation of new potential biomarkers for early detection of colon cancer. *PLoS One* 9, e106748 (2014).

39. Kaiser, S. *et al.* Transcriptional recapitulation and subversion of embryonic colon development by mouse colon tumor models and human colon cancer. *Genome Biol.* 8, R131 (2007).

40. Sheffer, M. *et al.* Association of survival and disease progression with chromosomal instability: a genomic exploration of colorectal cancer. *Proc. Natl. Acad. Sci. U. S. A.* 106, 7131–6 (2009).

41. Bastian, M., Heymann, S. & Jacomy, M. Gephi : An Open Source Software for Exploring and Manipulating Networks. *ICWSM* 8, 361–362 (2009).

42. Viechtbauer, W. Conducting Meta-Analyses in R with the metafor Package. *J. Stat. Softw.* 36, 7–10 (2010).

43. Sadanandam, A. *et al.* A colorectal cancer classification system that associates cellular phenotype and responses to therapy. *Nat. Med.* 19, 619–25 (2013).

44. De Sousa E Melo, F. *et al.* Poor-prognosis colon cancer is defined by a molecularly distinct subtype and develops from serrated precursor lesions. *Nat. Med.* 19, 614–8 (2013).

45. Masiero, M. *et al.* A core human primary tumor angiogenesis signature identifies the endothelial orphan receptor ELTD1 as a key regulator of angiogenesis. *Cancer Cell* 24, 229–41 (2013).

46. Bindea, G. *et al.* Spatiotemporal dynamics of intratumoral immune cells reveal the immune landscape in human cancer. *Immunity* 39, 782–795 (2013).

47. Huang, X. *et al.* Activation of the STAT3 signaling pathway is associated with poor survival in diffuse large B-cell lymphoma treated with R-CHOP. *J. Clin. Oncol.* 31, 4520–8 (2013).

48. Li, S. *et al.* Molecular signatures of antibody responses derived from a systems biology study of five human vaccines. *Nat. Immunol.* 15, 195–204 (2014).

49. Chiche, L. *et al.* Modular Transcriptional Repertoire Analyses of Adults With Systemic Lupus Erythematosus Reveal Distinct Type I and Type II Interferon Signatures. *Arthritis Rheumatol. (Hoboken, N.J.)* 66, 1583–95 (2014).

50. Newman, A. M. *et al.* Robust enumeration of cell subsets from tissue expression profiles. *Nat. Methods* 12, 1–10 (2015).

51. Subramanian, A. *et al.* Gene set enrichment analysis: a knowledge-based approach for interpreting genome-wide expression profiles. *Proc. Natl. Acad. Sci. U. S. A.* 102, 15545–50 (2005).

52. Culhane, A. C. *et al.* GeneSigDB--a curated database of gene expression signatures. *Nucleic Acids Res.* 38, D716-25 (2010).

53. Shaffer, A. L. *et al.* A library of gene expression signatures to illuminate normal and pathological lymphoid biology. *Immunol. Rev.* 210, 67–85 (2006).

54. Rosenwald, A. *et al.* Molecular diagnosis of primary mediastinal B cell lymphoma identifies a clinically favorable subgroup of diffuse large B cell lymphoma related to Hodgkin lymphoma. *J. Exp. Med.* 198, 851–862 (2003).

55. Compagno, M. *et al.* Mutations of multiple genes cause deregulation of NF-kappaB in diffuse large B-cell lymphoma. (Supplemental). *Nature* 459, 717–21 (2009).

56. Berry, M. P. R. *et al.* An interferon-inducible neutrophil-driven blood transcriptional signature in human tuberculosis. *Nature* 466, 973–977 (2010).

57. Ngo, V. N. *et al.* Oncogenically active MYD88 mutations in human lymphoma. *Nature* 470, 115–9 (2011).

58. Kidani, Y. *et al.* Sterol regulatory element-binding proteins are essential for the metabolic programming of effector T cells and adaptive immunity. *Nat. Immunol.* 14, 489–99 (2013).
